# Convergent Assembly of Homo‐ and Heterotypic Ubiquitin Chains from Functionalized, Expressed Monomers via Thiol‐Ene Chemistry

**DOI:** 10.1002/anie.202502638

**Published:** 2025-04-07

**Authors:** Moritz Urschbach, Susanne Huhmann, Dominik Appel, Luca Ferrari, Dominik Vogl, Sascha Martens, Christian F. W. Becker

**Affiliations:** ^1^ Faculty of Chemistry Institute of Biological Chemistry University of Vienna Währinger Straße 38 Vienna 1090 Austria; ^2^ Max Perutz Labs Vienna Biocenter Campus (VBC) Dr.‐Bohr‐Gasse 9 Vienna 1030 Austria; ^3^ Max Perutz Labs University of Vienna Dr.‐Bohr‐Gasse Vienna 1030 Austria; ^4^ Vienna Doctoral School in Chemistry (DoSChem) University of Vienna Währinger Str. 42 Vienna 1090 Austria

**Keywords:** Bioorganic chemistry, Protein modifications, Protein semisynthesis, Tau, Ubiquitin

## Abstract

Nature constructs ubiquitin tags with high spatiotemporal precision to execute defined functions that critically rely on the exact molecular composition of the ubiquitin chain. Deciphering the complex ubiquitin code is of paramount interest in biology and requires flexible access to homogeneous ubiquitin tags. As enzymatic approaches suffer from inherent drawbacks such as hardly controllable chain length or connectivity and substrate‐specificity, we apply a combination of expression and chemical tools to assemble ubiquitin chains. Our strategy includes expression of ubiquitin–intein fusion constructs to obtain large quantities of defined ubiquitin monomers with C‐terminal modifications such as hydrazides and propargylamides. Linkages between ubiquitins are generated via photoinitiated thiol‐ene click (TEC) chemistry, resulting in a nearly native isopeptide bond. We demonstrate the generation of homo‐ and heterotypic ubiquitin oligomers with K27, 29, 48, and 63 linkages up to a K48‐linked tetramer. The presented toolbox allows selective installation of ubiquitin on target peptides and proteins with reactive cysteine residues as demonstrated for segments of the microtubule‐associated protein tau. Such segments can be implemented into protein semisyntheses as shown here for ubiquitylated full‐length Tau4. The presented work combines minimal synthetic effort with high fidelity linkage chemistry, paving the way toward homogeneously ubiquitylated proteins.

## Graphical abstract

Generating functionalized ubiquitin building blocks via expression in *E. coli* paves the way to different di‐, tri‐, and tetra‐ubiquitin chains. Light‐induced thiol‐ene click reactions, combined with a masking and protection strategy, are harnessed to establish controlled ubiquitin chain assembly and to allow the semisynthesis of site‐selectively ubiquitylated Tau4.

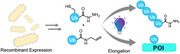

## Introduction

**Figure 1:**
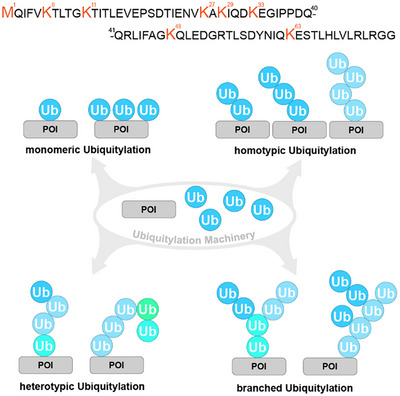
Diversity of ubiquitylation motifs. Top: primary sequence of ubiquitin with highlighted residues that allow for isopeptide bond formation. Schematic representation of various ubiquitylation patterns originating from monomeric units. Different ubiquitin colors represent varying connection sites. Proteins of interest (POI) are shown in grey.

Post‐translational modifications (PTMs) are key modulators of protein physicochemical properties and structure, thereby affecting function and localization. Although most PTMs are small with respect to molar mass, ubiquitylation relies on the attachment of 76 aa protein entities (ubiquitin) to a protein substrate.^[^ ^1^ ^]^ Another rare feature amongst PTMs displayed by ubiquitylation is the fact that besides single or multiple monomeric modifications, distinct oligomeric structures drastically increase the degree of structural and functional complexity, which is otherwise only observed for carbohydrates.^[^ ^2^ ^]^ In the case of ubiquitin, oligomerization is achieved via the formation of an isopeptide bond between a lysine side chain or the N‐terminus and the C‐terminal carboxylate of a second ubiquitin. The presence of seven lysine residues throughout the protein gives rise to a plethora of possible chain variants ranging from derivatives connected at the same position (homotypic) over chain types with varying connection sites (heterotypic) to branched variants (Figure 1).

Accordingly, ubiquitylation is involved in the control of a multitude of cellular processes such as protein homeostasis by proteasomal degradation and autophagy, DNA damage response, or cell division.^[^ ^1^, ^2^ ^]^ The functional study of specific ubiquitylation modifications is therefore of high interest in many research areas. However, the great structural complexity of ubiquitylation mostly prohibits isolation of homogeneously ubiquitylated proteins of interest, generating a high demand for innovative (bio)chemical strategies for controlled ubiquitylation.

Harnessing the natural enzymatic machinery has been successfully applied to obtain homotypic ubiquitin chains.^[^ ^3^, ^4^ ^]^ However, it is difficult to control chain length, the techniques are not applicable to all linkage sites due to limited availability of suitable enzymes, heterotypic or branched isomers are barely accessible, and enzyme's substrate specificity complicates the ubiquitylation of peptides or proteins.

By the incorporation of cysteine mutations^[^ ^5^ ^]^ or protected lysine residues by genetic code expansion,^[^ ^6^ ^]^ researchers were able to control attachment sites in substrate proteins as well as chain length in enzyme‐driven assembly strategies. On the other hand, the development of efficient synthetic and semisynthetic strategies toward homogeneous ubiquitin modifications has flourished recently.^[^ ^7^, ^8^ ^]^ Methods to selectively construct ubiquitin chains with native isopeptide bonds mostly rely on fully synthetic strategies combining solid‐phase peptide synthesis (SPPS) with native chemical ligation (NCL).^[^ ^9^, ^10^ ^]^ In these approaches, site‐selectivity is achieved by the use of protecting groups and/or temporarily installed auxiliaries. This enabled the synthesis of up to tetra‐ubiquitylated α‐synuclein,^[^ ^11^ ^]^ cyclin B1,^[^ ^12^ ^]^ or α‐globin.^[^ ^13^, ^14^ ^]^ However, as these strategies are rather labor intensive and the introduction of *δ*‐mercaptolysine can be challenging,^[^ ^8^ ^]^ attempts to facilitate the attachment to target proteins have been made by introducing nonnative linkages.^[^ ^15^, ^16^ ^]^ Such strategies additionally offer the opportunity to modulate chemical and enzymatic stability of ubiquitin linkages as has been demonstrated by reduction‐labile disulfide connections^[^ ^15^ ^]^ or oxime bonds stable against deubiquitylating enzymes (DUBs).^[^ ^16^ ^]^ The flexibility of fully synthetic approaches also provides access to branched chain derivatives of the native isopeptide bond by NCL.^[^ ^10^, ^17^ ^]^ Chemical strategies can also complement enzymatic routes, providing enhanced control over chain elongation.^[^ ^18^, ^19^ ^]^

As an alternative, protein semisynthesis^[^ ^20^ ^]^ has emerged as a powerful technique that combines the advantages of protein expression with the flexibility of chemical ligation procedures. This combinatorial approach provides access to larger proteinaceous structures, which can also be applied to the construction of ubiquitin chains and ubiquitylated proteins of interest.

To this end, expression of ubiquitin with subsequent global and transient protection of primary amines has been used for controlled assembly of native isopeptide‐linked di‐ubiquitin^[^ ^21^ ^]^ or branched tri‐ubiquitin chains.^[^ ^22^ ^]^ Other strategies investigated semisynthetic access to non‐native ubiquitin connections to tackle selectivity challenges in vitro and in cells. The Lang group designed ubiquitin building blocks with two C‐terminal mutations that are accessible to Sortase‐mediated ligation, enabling the construction of polymeric ubiquitin architectures^[^ ^23^ ^]^ as well as the attachment to proteins of interest (POIs) in cells.^[^ ^24^ ^]^ Genetic code expansion was also shown as a viable route to build ubiquitin chains via non‐native linkages,^[^ ^25^ ^]^ for example, by the introduction of azide^[^ ^26^, ^27^ ^]^ or hydroxylamine^[^ ^28^ ^]^ modified amino acids. These building blocks allow chemoselective formation of ubiquitin chains with alkyne or aldehyde‐tagged ubiquitin monomers, respectively. Cysteine‐aminoethylation assisted chemical ubiquitylation (CAACU) employing an alkylation‐ligation strategy with cysteine mutants was used to prepare up to linear tri‐ubiquitin chains.^[^ ^29^, ^30^, ^31^ ^]^

Another chemical connection approach was pursued by the Strieter group, exploiting cysteine mutants for radical‐initiated thiol‐ene chemistry (TEC) to selectively construct di‐ubiquitin chains as well as branched tri‐ubiquitin chains.^[^ ^32^ ^]^ By using a bifunctional building block, this chemistry can also deliver larger homotypic oligomers via unselective polymerization.^[^ ^33^ ^]^ The resulting homothialysine linkage was shown to be recognized and processed by DUBs at a similar level as native isopeptide‐linked ubiquitin.^[^ ^34^ ^]^

**Figure 2:**
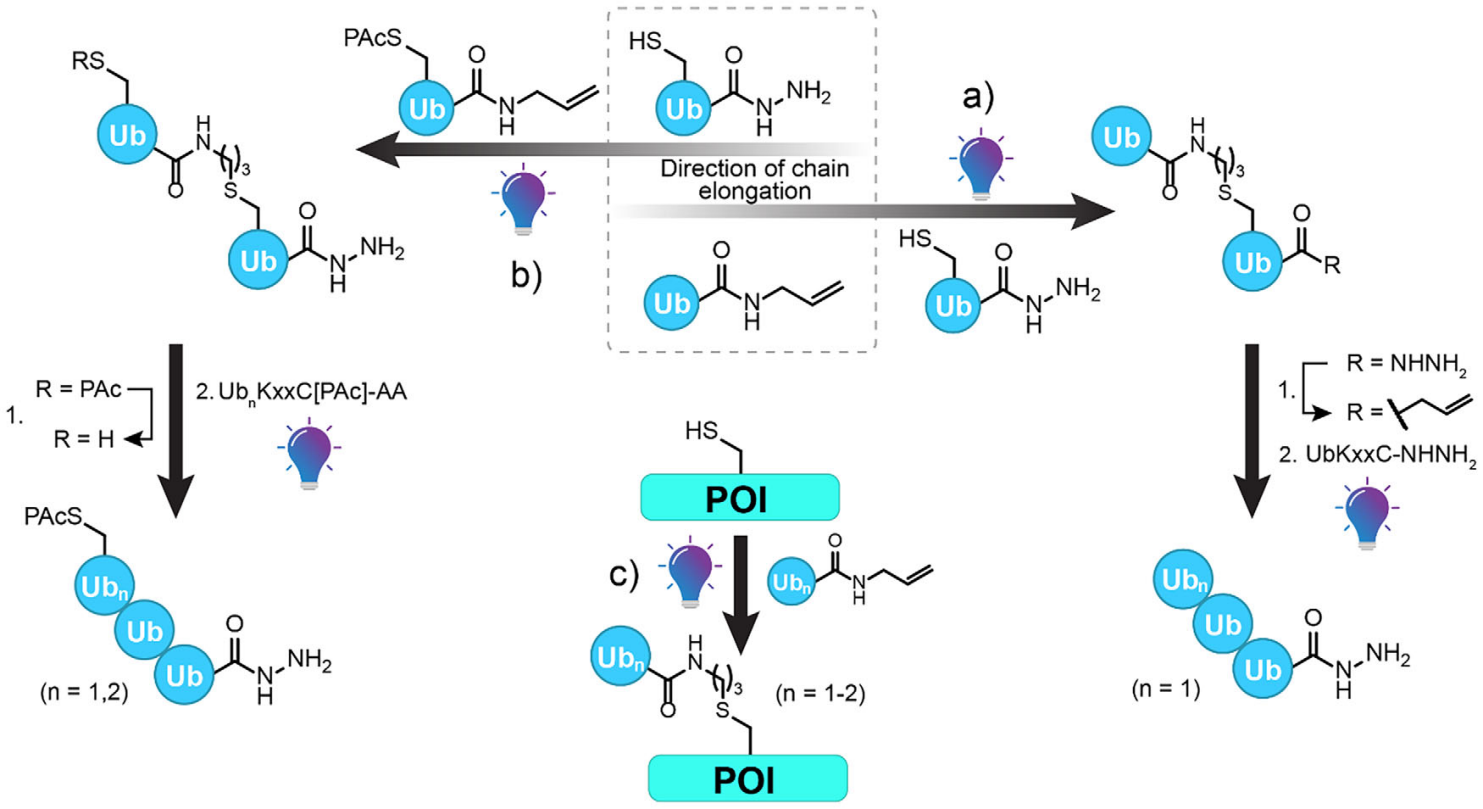
Semisynthetic strategy toward defined ubiquitin chains enabled by photoinitiated TEC. Hydrazide‐masked Ub(KxxC) C‐termini allow chain growth in the C‐terminal direction (Path a), whereas the use of PAc‐protected Ub(KxxC) allylamides allow chain elongation in the N‐terminal direction (Path b). The same chemical tools were used to transfer ubiquitin to a POI (Path c). Light bulb symbolizes irradiation at 365 nm.

To expand the scope of semisynthetic approaches toward controlled assembly of all kinds of ubiquitin oligomers and ubiquitylated POIs, we developed a flexible strategy that allows for iterative elongation of the ubiquitin chain with recombinantly produced ubiquitin buildings blocks and, at the same time, for the transfer to POI, all based on thiol‐ene chemistry. This can be achieved by combining photoinitiated TEC with a transient protection strategy for cysteines to modularly construct fully defined linear ubiquitin chains from monomeric building blocks (Figure 2). Depending on the recombinantly produced and protected/functionalized ubiquitin building blocks, chain elongation can be driven in two directions, either via the cysteine mutation or the C‐terminus.

To this end, we start with an initial wild‐type ubiquitin carrying a C‐terminal allylamide and employ Ub(KxxC) mutants (xx = 11, 27, 29, 48, 63) with a C‐terminal hydrazide to grow the chain. Ub(K6C) and Ub(K33C) mutants have not been produced here as K6, K27, K33 are reported to be the least abundant connections found in cells.^[^ ^35^ ^]^ However, as Strieter and coworkers reported K27 as particularly difficult linkage site, due to limited steric accessibility in the folded state,^[^ ^32^ ^]^ we decided to include Ub(K27C) to demonstrate feasibility of this approach even for this challenging site.

After the first elongation step via TEC, the C‐terminal hydrazide is converted into the corresponding allylamide to allow further C‐terminal elongation (Figure 2, Path a). On the other hand, protecting cysteine with phenacyl (PAc) directly enables the use of Ub(KxxC) allylamides to grow the chain in the opposite direction. Removal of the protection group after TEC permits another chain elongation step (Figure 2, Path b). Attachment of Ub chains to POIs with one or more cysteine residues is easily possible using the same chemistry (Figure 2, Path c). The use of recombinantly produced ubiquitin‐hydrazide building blocks in both strategies provides unprecedented flexibility in terms of linkage strategies and for late‐stage modification of POIs.

## Results and Discussion

**Figure 3:**
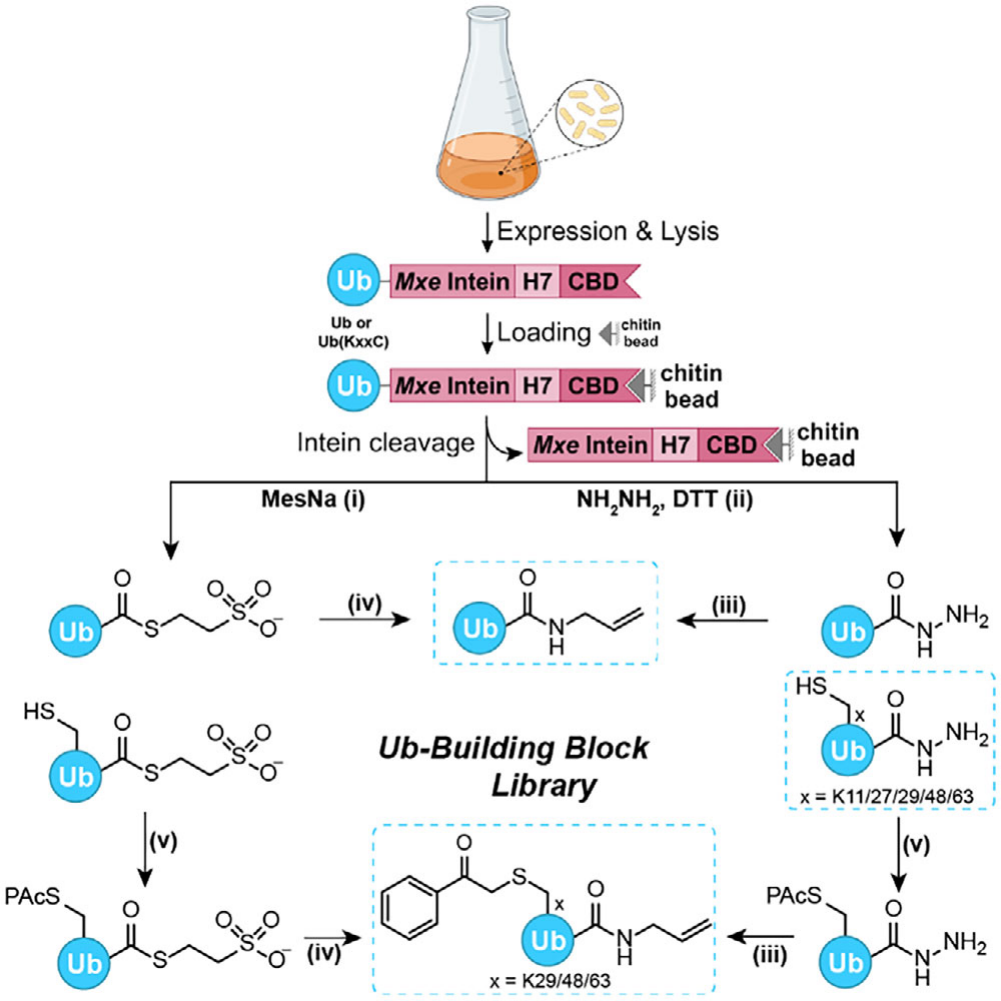
Generation of versatile monomeric ubiquitin building blocks by recombinant expression. 15–20 mg L^−1^ (*E. coli* at OD∼2) isolated product of UbWT‐NHNH_2_ and Ub(KxxC)‐NHNH_2_. Conditions for modification: i) MesNa (500 mM), PBS, pH 5.5, 48 h, 37 °C; ii) NHNH_2_ (2.5%), DTT (50 mM), TBS, pH 9.2, rt, 72 h; iii) 1. NaNO_2_, 200 mM NaP_i_, 6 M Gdn·HCl, pH 3, −15 °C, 20 min, 2. allylamine (500 mM), H_2_O, pH 9, 0 °C, 2 h; iv) allylamine (500 mM), H_2_O, pH 9, 0 °C, 2 h; (v) phenacyl bromide (2.5 equiv), 200 mM NaP_i_, 6 M Gdn·HCl, pH 7.2, rt, 2 h. H7, His_7_‐tag, CBD, chitin‐binding domain.

Efficient generation of monomeric ubiquitin building blocks in a multimilligram scale is essential for the presented modular strategy. As atom economy and costs for large scale synthesis of full‐length ubiquitin building blocks by SPPS are prohibitive, we employed recombinant expression in *E. coli* to produce C‐terminally modified ubiquitin building blocks with a series of Lys to Cys mutations (Figure 3). To this end, fusion constructs of wild‐type or ubiquitin mutants with Mxe GyrA intein as well as a chitin‐binding domain (CBD) were generated. All Ub(KxxC) mutants described here with xx = 11, 27, 29, 48, 63 were quickly accessible from the wild‐type encoding plasmid by site‐directed mutagenesis protocols. Expression for 2–3 h at 37 °C delivered up to 90 mg of fusion construct per liter of culture medium, which was subsequently subjected to affinity purification on chitin beads. Ubiquitin hydrazides or MesNa thioesters were generated as C‐terminal active functional groups, directly accessible from the fusion construct. Ubiquitin hydrazides (Ub‐NHNH_2_ or Ub(KxxC)‐NHNH_2_) were generated by direct hydrazinolysis^[^ ^36^, ^37^ ^]^ of the fusion construct on chitin beads using 2% hydrazine and 100 mM dithiothreitol (DTT). The ubiquitin thioesters (Ub‐SR or Ub(KxxC)‐SR) on the other hand were accessed by thiolysis^[^ ^38^ ^]^ with 500 mM sodium mercaptoethanesulfonate (MesNa). The intein cleavage reaction proceeds slowly, taking up to 3 d at room temperature or 37 °C. Of note, the hydrazinolysis reaction yields minor amounts of side product with +15 Da in mass (Figure S6), which is suspected to result from the conversion of an additional side chain amide into a hydrazide. This undesired side reaction can be contained by carefully adjusting the hydrazine concentration. All mutants were finally purified by preparative RP‐HPLC on a C4 stationary phase and obtained in yields ranging from 15 to 20 mg L^−1^ of expression medium, besides Ub(K27C)‐NHNH_2_, which was obtained in lower yields (∼5 mg L^−1^) (for analytical data see Supporting Information).

Although Ub(KxxC)‐NHNH_2_ variants were already suitable as starting material for TEC reactions, ubiquitin allylamide (Ub‐AA) was obtained either by direct aminolysis of 1.5 mg mL^−1^ Ub‐SR in 500 mM allylamine solution at pH 10.5 for 2 h or upon oxidative activation of Ub‐NHNH_2_ with 10 equiv NaNO_2_ at −15 °C for 20 min at pH 3 as acyl azide,^[^ ^39^ ^]^ followed by aminolysis with 500 mM allylamine at pH 9 and 0 °C for 2 h. Both routes provided Ub‐AA on average in 65% isolated yield (Figure S14), whereas C‐terminally hydrolyzed ubiquitin was only obtained as a very minor byproduct (less than 5%).

To vary the direction of ubiquitin chain elongation, thiol‐protected Ub[KxxC(PAc)]‐AA derivatives were generated from precursors either carrying C‐terminal thioester or hydrazide modifications (Ub(KxxC)‐SR and Ub(KxxC)‐NHNH_2_). In both cases, chemoselective introduction of the PAc group was achieved by reaction with 2.5 equiv phenacyl bromide in 200 mM sodium phosphate (NaP_i_), 6 M guanidinium chloride (Gdn·HCl) at pH 7.1 within 2 h,^[^ ^40^ ^]^ whereas the hydrazide nucleophile is not affected (Figure S24). Subsequently, the Ub[KxxC(PAc)]‐SR derivatives could be converted to the corresponding allylamide Ub[KxxC(PAc)]‐AA in one‐pot by addition of allylamine, whereas the Ub hydrazide Ub[KxxC(PAc)]‐NHNH_2_ was concentrated to 5 mM, adjusted to pH 3 before oxidative activation by NaNO_2_, followed by aminolysis with 500 mM allylamine and final purification by RP‐HPLC. The PAc‐protected allylamide building block was generated for the K29C, K48C, and K63C mutant (Figures S21,S25,S28). As ubiquitin C‐terminal thioesters and hydrazides are generally available for reactions with alternative nucleophiles (such as propargylamine) or electrophiles (such as aldehydes), respectively, this strategy provides the opportunity to vary the type of linkage chemistry if for example a nonhydrolyzable connection (e.g., a triazole^[^ ^41^ ^]^) is preferred. To this end, ubiquitin propargylamide was generated from our Ub variants as an exemplary compound (Figure S15).

With the library of monomeric ubiquitin building blocks in hand, we moved on to establish a robust and broadly applicable photoinitiated TEC for Ub chain construction. Based on previous work by the Strieter group,^[^ ^32^, ^42^ ^]^ lithium phenyl‐2,4,6‐trimethylbenzoylphosphinate (LAP) was chosen as water‐soluble photoinitiator that undergoes homolytic cleavage upon irradiation at 365 nm. Our investigations confirmed LAP to be the most suitable among various tested thermal and photoinitiators (Table S1 and Figures S7–S11). Initial experiments using 1 mM wildtype ubiquitin allylamide and Ub(K63C)‐NHNH_2_ with 0.5 mM LAP in 250 mM NaOAc buffer pH 5.1^[^ ^32^ ^]^ yielded only minor amounts of di‐ubiquitin after 1 h of irradiation with a 365 nm curing lamp placed on top of the reaction mixture in an Eppendorf tube. However, as the phosphorous radical resulting from photocleavage of LAP is quite reactive toward olefins,^[^ ^42^ ^]^ ubiquitin phosphinate^[^ ^32^ ^]^ was obtained as major product besides unreacted starting material. The reaction conditions were optimized by increasing the concentration of the reactants to 6 mM of ubiquitin allylamide and 1.25 equiv of the thiol component. To provide sufficient solubility, denaturing conditions were chosen by using an aqueous buffer with 250 mM NaOAc at pH 5.5 containing 6 M Gdn⋅HCl. The denaturant should also help to improve the accessibility of linkage positions in Ub that are not directly exposed on its surface in the folded state. Applying 1.5 mM TCEP also proved to be beneficial by preventing oxidation of the linkage site and efficient mixing of the solution was observed to be a critical parameter as was working at temperatures of −10 to 0 °C with decreased viscosity. The selective TEC chemistry is well tolerated by all other groups present in Ub such as lysine and methionine side chains.

**Figure 4:**
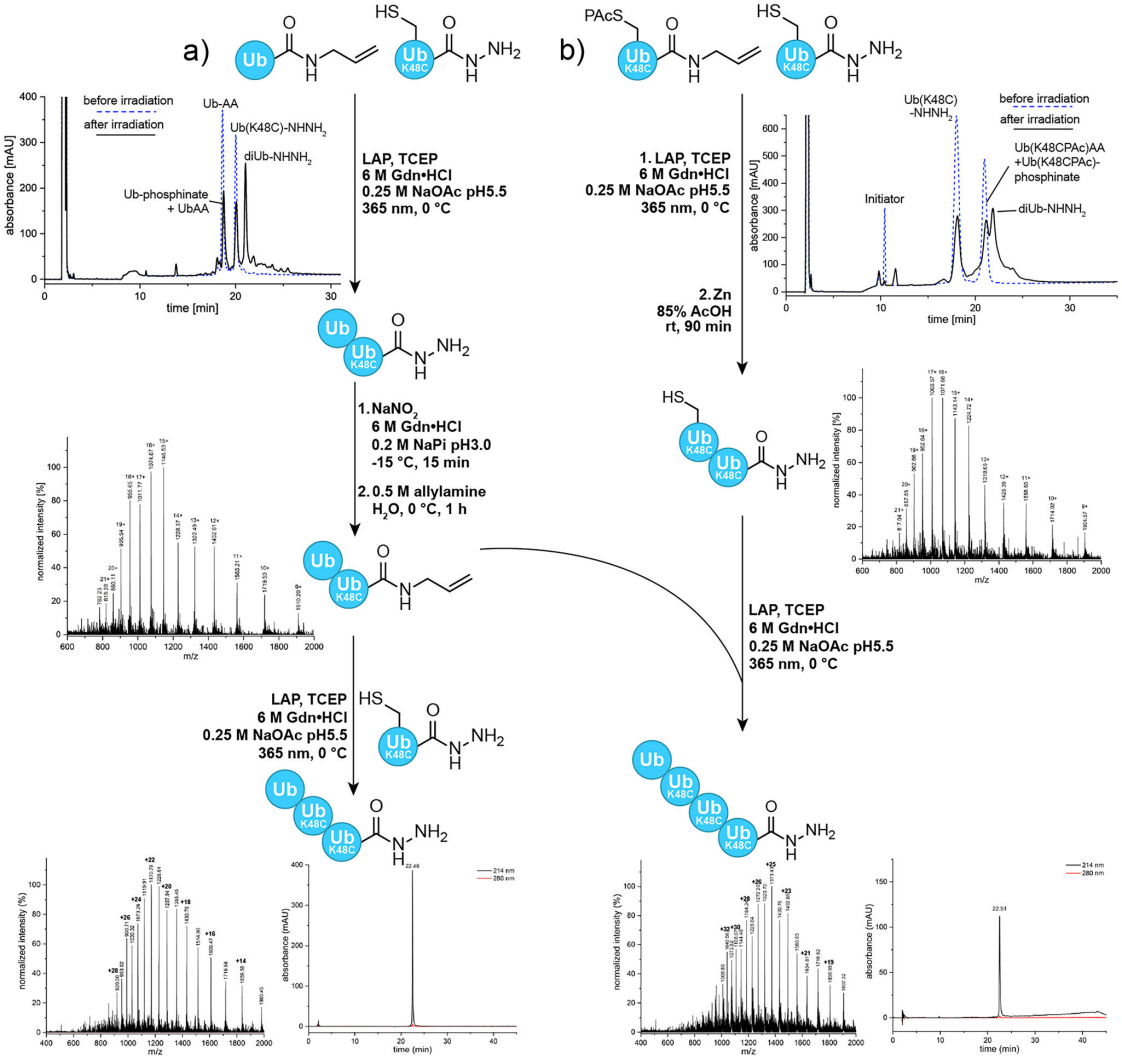
Construction of defined Ub(K48C) chains by consecutive photoinitiated TEC reactions. Synthesis strategy a) describes the consecutive C‐terminal elongation toward triUb(48/48)‐NHNH_2_ starting with a terminal wildtype UbAA. Selected analytical data graphs from top to bottom: analytical RP‐HPLC of the reaction mixture before (dashed blue line) and after (solid line) irradiation shows conversion to diUb(48)‐NHNH_2_; ESIMS (pos.) of diUb(48)‐AA [obs.: 17 182 Da, calc.: 17 184 Da]; ESIMS (pos.) of triUb(48/48)‐NHNH_2_ [obs.: 27 738 Da, calc.: 27 737 Da], and analytical RP‐HPLC (C4) of triUb(48/48)‐NHNH_2_ are displayed next to the structures. Synthesis strategy b) describes the consecutive elongation into the opposite direction starting from Ub(K48C)‐NHNH_2_ to build tetraUb(48/48/48)‐NHNH_2_. Selected analytical data graphs from top to bottom: analytical RP‐HPLC of the reaction mixture before (dashed blue line) and after (solid line) irradiation shows conversion to diUb(48/48(PAc))‐NHNH_2_: ESIMS (pos.) of diUb(48/48)‐NHNH_2_ [obs.: 17 182 Da, calc.: 17 184 Da], ESIMS (pos.) of tetraUb(48/48/48)‐NHNH_2_ [obs.: 34 316 Da, calc.: 34 315 Da], and analytical RP‐HPLC (C4) of tetraUb(48/48/48)‐NHNH_2_ are displayed next to the structures.

Under these conditions, the reaction was very fast and maximum conversion was achieved after 1 min of irradiation only. Figure 4a shows an optimized reaction scheme for the generation of triUb(48/48)‐NHNH_2_. The analytical RP‐HPLC of the crude TEC reaction mixture during synthesis of diUb(48)‐NHNH_2_ shows the product as major species next to excess of Ub(K48C)‐NHNH_2_ as well as the previously described phosphinate side product. Purification of the reaction mixture via RP‐HPLC on a C4 stationary phase at 60 °C delivered up to 30% of diUb(48)‐NHNH_2_ as isolated yield. It was important to either directly purify or freeze the reaction mixture after irradiation as the mesityl aldehyde derivative generated upon H‐abstraction by the initiator acyl radical fragment tends to react with the hydrazide moiety resulting in acyl hydrazones. The reaction could be scaled up to >3 µmol of Ub‐AA; however, scaling the purification conditions becomes more challenging. DiUb derivatives were synthesized with K27, K29, K48, and K63 linkages in overall similar yields of 15%–30% (Table S2). Interestingly, we did not encounter challenges for K27 and K29 linkages that have been previously reported to be difficult to generate under nondenaturing conditions.^[^ ^32^ ^]^ In contrast, K11‐linked di‐ubiquitin was not obtained with our approach. We could only isolate minor amounts of a yet unidentified side product with a mass difference of +335 Da. The decreased reactivity may be attributed to an increased hydrophobicity in the local environment compared to the other cysteine mutants (Figure 1, sequence), resulting in a lower degree of polarization.

For further chain elongation, diUb(48)‐NHNH_2_ was converted into the corresponding allylamide diUb(48)‐AA quantitatively as described for the mono‐ubiquitin building blocks (Figure 4a). However, purification by C4 RP‐HPLC resulted in significant product loss, prompting us to isolate di‐ubiquitin allylamide by ultrafiltration rather than RP‐HPLC in sufficient purity (>96%, Figure S32). The iterative synthesis strategy allowed the reaction of diUb(48)‐AA with another molecule of Ub(K48C)‐NHNH_2_ under similar conditions as described above. The diUb building block is sufficiently soluble under denaturing conditions to reach 6 mM concentration after sonication and freeze–thaw cycles. Irradiation for 3 × 15 s in the presence of 4 mM LAP and 2 mM TCEP resulted in the generation of homotypic linear triUb(48/48)‐NHNH_2_ that could be isolated after semipreparative RP‐HPLC in 16% yield (Figure 4a, bottom left). The same strategy, by reaction with Ub(K63C)‐NHNH_2_, was applied to produce heterotypic triUb(48/63)‐NHNH_2_ in 9% yield.

**Figure 5:**
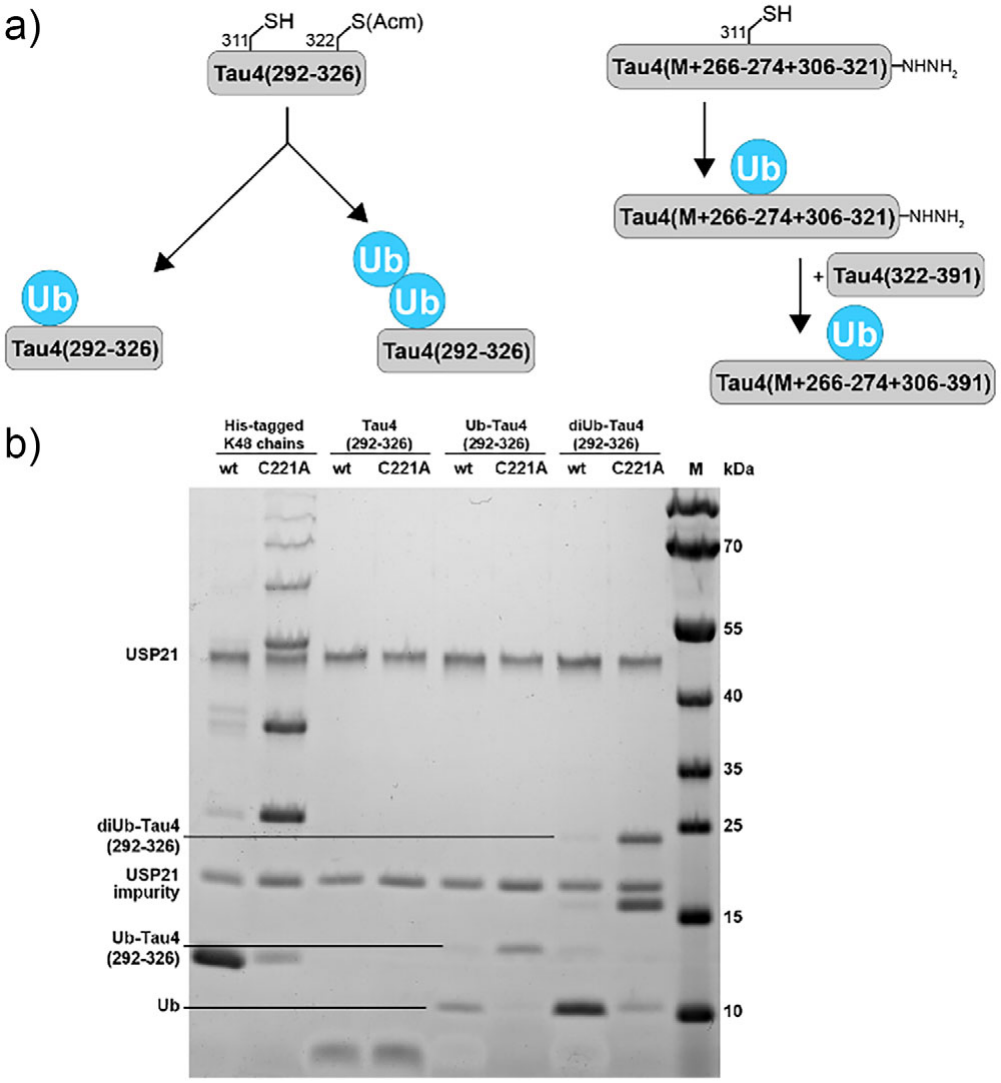
Schematic representation of a) ubiquitylated Tau4 peptides and b) deubiquitylation assay with USP21. SDS‐PAGE of reaction mixtures incubated 60 min with functional (wt) USP21 and nonfunctional (C221A) USP21 mutant. Lane 1 + 2: control with His‐tagged K48 chain mixture; lane 3 + 4: unmodified Tau4(292–326); lane 5 + 6: Ub‐ Tau4(292–326); and lane 7 + 8: diUb(wt‐48)‐Tau4(292–326).

Besides elongation of the growing ubiquitin chain at the C‐terminus, addition of Ub(KxxC[PAc])‐AA building blocks to a C‐terminal Ub(KxxC)‐NHNH_2_ allows for extension on the side chain (Figure 4b). Generation of PAc‐protected diUb(48(PAc)/48)‐NHNH_2_ was possible using similar reaction conditions as previously established for diUb(48)‐NHNH_2_. The irradiation intensity had to be diminished to 45 mW cm^−2^ as initial experiments showed partial cleavage of the PAc group during irradiation, which is a known feature of ketone‐based groups that can form suitable radical leaving groups.^[^ ^43^ ^]^ Complete removal of the PAc group was achieved under reducing conditions by diluting the reaction mix with 50% aqueous AcOH and by addition of Zn powder. Deprotected diUb(48/48)‐NHNH_2_ was finally isolated by RP‐HPLC in 17% yield. Overall, isolated yields obtained via synthesis Route b (Figure 4) were generally lower than via Route a. The method was also applied to generate diUb building blocks from different Ub(KxxC) mutants that can provide access to heterotypic triUb chains. To this end, the regioisomers diUb(29/63)‐NHNH_2_ and diUb(48/63)‐NHNH_2_ were produced (Figures S36,S37). The reaction toward a heterotypic triUb was finally demonstrated by capping the corresponding di‐ubiquitin building blocks with Ub‐AA via TEC reaction to deliver triUb(48/63)‐NHNH_2_ or triUb(29/63)‐NHNH_2_ (Figures S40,S41). Combining both synthesis strategies opens a convergent approach toward tetraUb by using both allylamide modified diUb(xx)‐AA as well as diUb building blocks with a free cysteine side chain. Such a convergent strategy was applied to obtain linear homotypic tetraUb(wt/48/48/48)‐NHNH_2_ (Figure 4b, bottom right). The isolated yield of the final assembly reaction was 6%, clearly indicating challenges with designing the TEC reaction conditions with such large building blocks (each ∼17 kDa) and with separating di‐ and tetraUb chains efficiently via RP‐HPLC. Based on these challenges longer chains will be even more difficult to access and we envision our flexible, convergent assembly routes for homo‐ and heterotypic linear Ub chains to be suitable for up to tetramers with linkages via K 27, 29, 48, and 63. Such chains should be sufficient to study the effects of different Ub linkage types and chain lengths on target proteins by providing direct access to homogeneously modified proteins. To this end, we have implemented the addition of Ub into the semi‐synthesis of full‐length 2N4R Tau (also termed TauF or Tau4) protein. The investigation of PTMs in the microtubule‐associated protein Tau is of particular interest as they play a key role in the development of neurodegenerative diseases.^[^ ^44^, ^45^, ^46^ ^]^ Hence, the construction of site‐selectively modified Tau variants has been addressed via semisynthetic^[^ ^47^, ^48^, ^49^, ^50^ ^]^ and by a combination of recombinant with enzymatic and bioorthogonal methods.^[^ ^51^ ^]^ Furthermore, disulfide conjugation was shown to be a suitable tool for selective late‐stage modification of Tau motifs when handling under reductive conditions is not necessary.^[^ ^52^, ^53^ ^]^ Mono‐ and poly‐ubiquitylation of Tau was observed on several residues, including K311 and K317,^[^ ^54^, ^55^ ^]^ which are among the most distinct PTMs in samples derived from Alzheimer's patients and absent in samples derived from healthy persons.^[^ ^56^ ^]^ Thus, we aimed to generate derivatives of the Tau4 isoform ubiquitylated at residue 311. To achieve this goal, we started with peptide segments as model systems to demonstrate that besides flexible Ub chain assembly, the TEC approach enables chemoselective modification of target peptides and proteins carrying a single free cysteine side chain with Ub (chains) (Figure 5a).

As first example, a 26mer Tau‐derived peptide (M + aa 266–274+306–321; Tau2N3R numbering: M+266–360), harboring a K311C mutation, was mono‐ubiquitylated using similar conditions as those used for diUb synthesis. The desired product was isolated in 35% yield (Figure S47). The reaction yield can be significantly increased by using a larger excess of the thiol‐containing Tau peptide (this was probed in a range from 7.5 to 30 mM, Figure S43). Therefore, the ubiquitylation method seems to be especially suitable for substrates with medium to high solubility. With Ub‐Tau4(M+266–274+306–321) in hand, we could further demonstrate that a peptide fragment ubiquitylated via TEC can serve as starting material in a fragment condensation. By oxidative activation of the hydrazide and 4‐mercaptophenylacetic acid (MPAA)‐mediated ligation with Tau4(322–391), the ubiquitylated 96mer fragment Ub‐Tau4(M+266–274+306–391) was constructed (Figure S48).

In order to extend this approach, we modified another Tau‐derived peptide (aa 292–326) with a K311C mutation and a native Cys residue that required protection with acetamidomethyl (Acm) to allow site‐selective attachment of ubiquitin to position 311 under similar conditions as described above (Figure S44). After a desalting step, the Acm group could be removed using AgOAc in 85% AcOH^[^ ^57^ ^]^ before a final RP‐HPLC purification yielded 21% of monoubiquitylated Ub‐Tau4(292–326) (Figure S45). Furthermore, di‐ubiquitylation of the same peptide Tau4(292–326) was demonstrated (Figure S46). The homothialysine linkage in di‐ubiquitin motifs was shown to lead to similar average structures as well as similar reactivity profiles in DUB assays compared to natively linked diubiquitin.^[^ ^34^ ^]^ To additionally confirm the applicability of the homothialysine linkage as mimic for ubiquitin‐peptide/protein connection, we performed DUB cleavage assays using mono‐ and di‐ubiquitylated Tau4(292–326) peptides as substrate (Figure 5). SDS‐PAGE analysis of the reaction mixtures confirmed that treatment with Ubiquitin carboxyl‐terminal hydrolase 21 (USP21)^[^ ^58^ ^]^ lead to cleavage of the ubiquitin from the substrate peptide.

**Figure 6:**
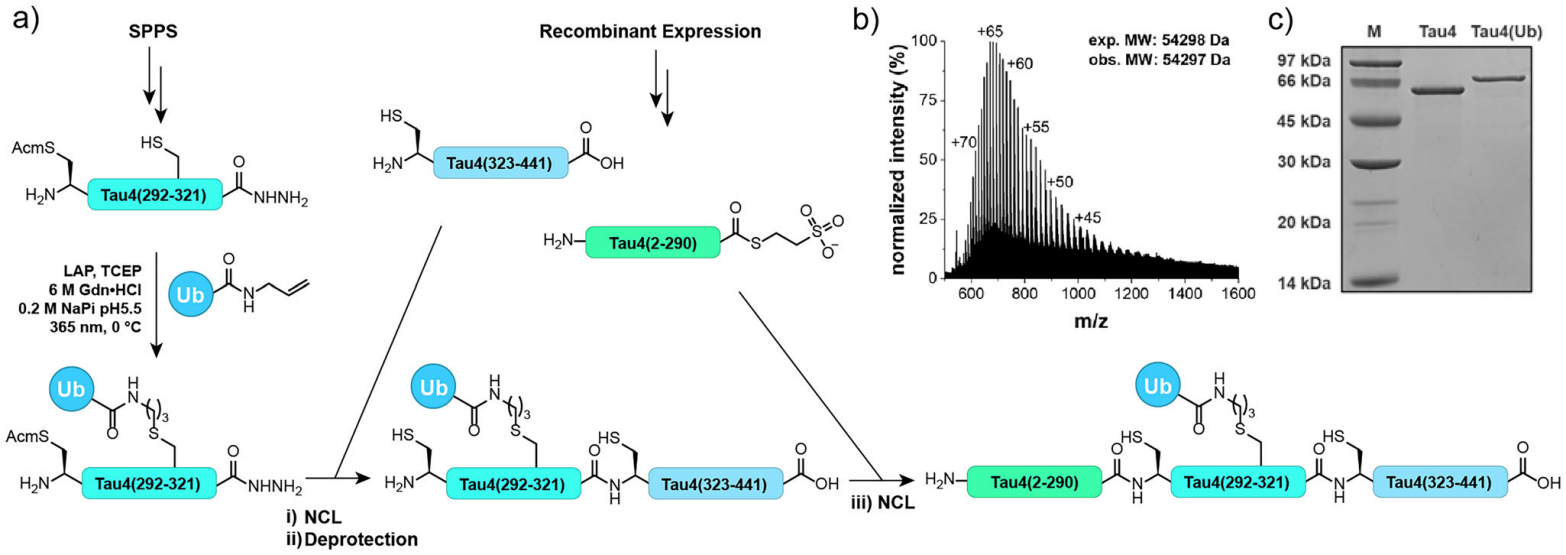
Semisynthesis of ubiquitylated Tau4(2–441). a) Synthesis scheme: i) 1. NaNO_2_, 6 M Gdn·HCl, 200 mM NaP_i_ buffer, pH 3, −15 °C, 20 min, 2. 300 mM MPAA, pH6.8, rt, 5 min, 3. TauC(322–441), pH6.8, 30 °C, 3 h; ii) AgOAc (1 mg per100 ul, 85% AcOH, 30 °C, 1 h; iii) 200 mM MPAA, 100 mM TCEP, 6 M Gdn·HCl, 200 mM NaP_i_ buffer, pH 7.1, 37 °C, 3 h. b) ESIMS of Ub‐Tau(2–441) (exp. M.W.: 54 298 Da; obs. M.W.: 54 297 Da). c) SDS‐PAGE of Ub‐Tau(2–441), Coomassie stain.

Selective protection strategies for Cys residues pave the way toward more flexible ubiquitylation strategies (in synthetic peptides) and provide access to larger proteins via chemoselective ligation methods.^[^ ^59^ ^]^ Relying on a previously reported semisynthetic strategy,^[^ ^49^ ^]^ the full‐length Tau 2N4R isoform ubiquitylated at position 311 was constructed from three segments (Figure 6). The synthetic middle fragment Tau4(291–321)‐NHNH_2_ with a K311C mutation for ubiquitylation by TEC, also contains one native Cys291 that is utilized for NCL with an N‐terminal recombinant segment (Tau4(2–290)). The two recombinant segments Tau4(2–290)‐MesNa and C‐terminal segment Tau4(322–441) were generated by expression in *E. coli* (for details see Ref. [49]). Ub‐Tau4(291–321)‐NHNH_2_ was prepared by reaction with an excess (1.25 equiv) of UbAA in the presence of 4 mM LAP to yield 23% after RP‐HPLC isolation. The C‐terminal hydrazide of Ub‐Tau4(291–321)‐NHNH_2_ was converted into the MPAA thioester^[^ ^60^ ^]^ and ligation with Tau4(322–441) was carried out in situ using final concentrations of 3 mM of thioester and 2 mM of the Tau4(322–441) under denaturing conditions resulting in ∼90% conversion after 90 min. Ub‐Tau4(291–441) was isolated in 30% yield after RP‐HPLC purification. Removal of the Acm group from Cys291 was achieved by treatment with AgOAc in aqueous acetic acid^[^ ^57^ ^]^ to yield 47% of unprotected Ub‐Tau4(291–441). In the final step, Tau4(2–290)‐MesNa thioester (4.0 mM) was ligated to Ub‐Tau4(291–441) (0.75 mM) using 200 mM of MPAA additive in 6 M Gdn⋅HCl at pH 7.1. The reaction proceeded to 95% conversion within 3 h and was purified by RP‐HPLC to yield 41% of Ub‐Tau4(2–441) (Figure 6).

With monoubiquitylated Tau in hand, we analyzed its aggregation behavior. Aggregation of monomeric Tau4 is a critical hallmark in neurodegenerative diseases such as Alzheimer's disease, which is significantly affected by PTMs displayed on the protein.^[^ ^45^ ^]^ To gain insights into the influence of a ubiquitin motif on the aggregation of full‐length Tau, we analyzed Ub‐Tau4(2–441) in comparison to recombinant unmodified Tau4(2–441) by an in vitro thioflavin T (ThT)‐based fibrillization assay (Figure S59). Upon addition of octadecyl sulfate (ODS, 45 µM) to a 2 µM solution of Tau4 protein, the aggregation was monitored by increasing ThT fluorescence. For Ub‐Tau4(2–441), no increase in fluorescence intensity was detected within 24 h, whereas the unmodified control Tau4(2–441) started to form fibrils after <100 min and reached a plateau after ∼12 h, which is in excellent agreement with previously observed aggregation behavior of Tau.^[^ ^61^ ^]^ Fibril formation for Tau4 was further confirmed by SEM analysis showing fibrillar morphology for Tau4(2–441), whereas no fibrils were observed for Ub‐Tau4(2–441) (Figure S59). These results suggest that aggregation of Tau4(2–441) is suppressed by the presence of a single ubiquitin modification at position 311 in the microtubule binding domain. In contrast to this finding, a version of the microtubule‐binding four repeat domain (4RD) ubiquitylated at K311 analyzed in recent studies showed fibrillar aggregation behavior in ThT assays and TEM, although with an increased lag‐phase and reduced growth rate compared to unmodified 4RD.^[^ ^53^, ^62^ ^]^ This suggests that ubiquitylation may induce different effects when analyzed in a full length protein setting compared to protein segments.

## Conclusion

The facile generation of sequence‐defined ubiquitylation patterns is a key prerequisite toward understanding the complex biology of the ubiquitin system as well as the study of functional implications of these PTM. To this end, we contributed by establishing a semisynthetic strategy based on consecutive photoinitiated TECs.^[^ ^32^ ^]^ The approach is highly modular relying on recombinantly generated monomeric ubiquitin building blocks. Overall, we were able to produce homo‐ and heterotypic tri‐ubiquitin chains as well as homotypic tetra‐ubiquitin chains. The linkage chemistry was finally demonstrated to be suitable for the regioselective modification of Tau‐derived peptides, which were ultimately harnessed to construct a site‐selectively ubiquitylated Tau4 protein by NCL. The latter was shown to inhibit fibrillization in comparison to unmodified Tau4 within the timeframe of the assay, similar to reports on other ubiquitylated, aggregation‐prone proteins such as α‐synuclein.^[^ ^11^ ^]^

## Conflict of Interests

The authors declare no conflict of interest.

## Supporting information



Supporting information

## Data Availability

The data that support the findings of this study are available in the Supporting Information of this article.
