# Supplementary material for: Convergent Assembly of Homo‐ and Heterotypic Ubiquitin Chains from Functionalized, Expressed Monomers via Thiol‐Ene Chemistry
Source: Angew Chem Int Ed Engl. 2025 Apr 7;64(21):e202502638. doi: 10.1002/anie.202502638 (PMC12087842; doi:10.1002/anie.202502638)
Supplement: Supplementary file 1 — Supporting information [file ANIE-64-e202502638-s001.pdf]

Table of Contents

Materials and methods 1
Generation of Monoubiquitin building blocks 4
Experimental procedures 4
Characterization of mono-ubiquitin building blocks 13
Generation of oligo-ubiquitin chains 30
Characterization of di-ubiquitin motifs 30
Characterization of tri-ubiquitin motifs 40
Charactzerization of tetra-ubiquitin 43
Ubiquitylated tau peptides 44
Synthesis and Characterization of Ub-Tau4(2-441) 51
Amino acid and gene sequences 58

Materials and methods

Reagents

H_2O was purified by a Milli-Q®Integral Water Purification System. Acetonitrile (HPLC gradient grade) and Methanol were obtained from Sigma-Aldrich. MPAA and TCEP were obtained from Sigma-Aldrich or TCI. Allylamine was purchased from Sigma-Aldrich. Allylamine hydrochloride was purchased from Fluorochem. Lithium phenyl(2,4,6-trimethylbenzoyl)phosphinate was purchased from Sigma-Aldrich or BLDPharm (a new bottle was used after 12 month). Guanidinium chloride, Na_2HPO_4, and NaH_2PO_4 were purchased from Sigma-Aldrich. Yeast extract, tryptone and NaCl were obtained from AppliChem. Chemicals and enzymes for cloning were obtained from Agilent Technologies, Promega or New England Biolabs. The PCR purification was obtained from Qiagen, the Gene JET Plasmid MiniprepKit was purchased from Thermo Fisher Scientific and sequencing was performed by Eurofins Genomics AT. Chitin beads were purchased from New England Biolabs.

HPLC and mass spectrometry:

Analytical HPLC analyses were performed either on a Thermo Scientific Vanquish HPLC system or a Dionex Ultimate 3000 HPLC system. Samples were analyzed either on a Macherey-Nagel Nucleodur 300-5 C4ec 4.6x150 mm column or a Waters XBridge Protein BEH C4, 300-3.5, 4.6x150 mm. H_2O modified with 0.1% TFA (A) as well as ACN modified with 0.08% TFA (B) were

used as eluents in gradient elution profiles. Typical analyses were performed using the following gradient elution profiles at a flow rate of 1 mL/min:

- 5%–65%B in 30 min.
- 5%–40%B in 40 min.
- 5%–25%B in 3 min, 25%–40%B in 40 min.

Chromatograms were recorded at 214 nm and 280 nm.

Preparative and semi-preparative HPLC purifications were performed either on a binary Varian ProStar system, a quaternary Waters Prep 150 LC system or a quaternary Shimadzu Prominence system. Semi-preparative scale HPLC purifications were conducted on a Kromasil 300-10-C4 10x250 mm column, a Waters XBridge Protein BEH C4 10×250 mm column, a Macherey-Nagel Nucleodur 300-10 C4 10×250 mm column, a Kromasil 300-10-C18 21.2x250 mm column, Kromasil 300-10-C18 10x250 mm column. H_2O modified with 0.1% TFA (A) as well as ACN modified with 0.08% TFA (B) were used as eluents in gradient elution profiles that are described in the individual synthesis procedure sections. Collected fractions were analyzed by mass spectrometry, product containing fractions were pooled and subjected to lyophilization.

Mass spectra were recorded on a Waters Auto Purification HPLC/MS system equipped with a 3100-mass detector or a Waters Arc LC coupled with a with a SQD2 mass detector using electrospray ionization (ESI) in positive ion mode. High-resolution mass spectra were recorded either on a QExactive orbitrap mass spectrometer, a Bruker maxis or timsTOF flex mass spectrometer.

SDS-PAGE:

SDS-PAGES were performed using self-casted 15% acrylamide gels under reducing conditions. Samples were loaded in after 1:1 mixing with 2x SDS-loading buffer (0.5 M Tris-HCl, 6% (w/v) SDS, 35% (v/v) glycerol, 3.5% (v/v) β-mercaptoethanol and 0.05% (w/v) bromophenol blue, pH 6.8). A low molecular weight marker kit from GE Healthcare was used as molecular weight reference. The gels were stained with a Coomassie brilliant blue R-250 solution (0.1% (w/v) Coomassie R250, 10% (v/v) AcOH and 45% (v/v) MeOH in H_2O a and imaged using a Bio-Rad ChemiDoc MP imaging system.

ThT Assay:

The setup for the Thioflavin T (ThT) aggregation assays was based on previously described protocols by us and others.[1-3] Following stock solutions were prepared: 5x aggregation buffer (50 mM HEPES.KOH, 500 mM NaCl, pH 7.4, 5 mM DTT (added freshly)), 40 mM HEPES.KOH (pH 7.5), 2 mM ODS (octadodecyl sulfate) in 50% (v/v) aqueous isopropanol and 100 µM Thioflavin T (ThT) in ddH2O. All buffers and solvents were prepared, filtered (syringe filter pore size: ≤ 0.22 µm) and either kept at 4 °C for short time storage or kept at -20 °C for long time storage. For the aggregation assays lyophilized aliquots of the Tau protein variants were freshly dissolved in

appropriate volumes of ddH2O to yield ~1 mg/mL. Exact protein concentrations were determined using a Pierce™ BCA Protein Assay Kit (Thermo Fisher, USA). Fibril formation assays were performed at a final concentration of 2 µM for each Tau2N4R variant in ThT aggregation buffer. The buffer is composed of 80 µL 5x aggregation buffer, 18.2 µL of HEPES.KOH, the appropriate volume determined for each Tau2N4R variant and filled up with ddH2O to a final volume of 390 µL. Afterwards, the aggregation was induced by adding 10 µL of ODS (or 10 µL of aqueous isopropanol for non-induced controls) and 44 µL of the ThT stock solution was added. The ThT assay was performed in 96-well microplates from Greiner Bio-One (black, non-binding, chimney-well). Each well contained a glass bead (Merck, Germany) for improved mixing. The microplate was closed with adhesive foil to avoid loss of sample volume due to evaporation during the experiment. The aggregation process was monitored via ThT fluorescence (excitation = 444 nm; emission = 490 nm) at 37 °C with continuous shaking on a microplate-reader (BioTek Synergy Mx). Fluorescence emission values were recorded every 5 min for 24 h. Fluorescence emission values at 30 min were subtracted from the mean of the individual technical triplicate and set as a starting point for the aggregation kinetics. Each experiment was performed in triplicate and repeated at least one time.

SEM

Samples for scanning electron microscopy (SEM) were taken immediately after the end of the aggregation assay. For SEM 10 µL of the ThT assay solution was directly applied to a carbon supported copper grid (Plano GmbH, Germany), incubated for 3 min, 1x whisk-washed with 10 µL of ddH2O and air-dried. The grids were fixed on the sample holder, sputter coated with gold nanoparticles under high vacuum (Bal-Tec SCD 005) and analyzed via SEM (Zeiss SEM Supra 55 VP).

Generation of Monoubiquitin building blocks

Experimental procedures

Recombinant Expression:

Recombinant expression of the ubiquitin building block was performed as Ub(wt or KxxC)-Mxe GyrA intein-chitin binding domain fusion protein. Protein expression was carried out in the *E. coli* BL21(DE3) Gold strain (Agilent) using 2YT medium (16 g/L Trypton, 10 g/L Yeast extract, 5 g/L NaCl) supplemented with 100 µg/mL ampicillin at 37 °C. Overnight cultures were diluted to OD600 = 0.2, grown until OD600 reached 0.7, and protein overexpression was induced with 1 mM IPTG. After 2–3 h the culture was centrifuged for 20 minutes at 10000 g, and the cell pellets were resuspended in TBS buffer (50 mM Tris, 150 mM NaCl, pH 8) and lysed using an ultrasound cell disruptor. The lysate was centrifuged for 40 minutes at 30000 g. Each 40 mL of supernatant (SN) was incubated with 5 mL TBS-equilibrated chitin resin (NEB) for 2 hours at room temperature. The resin was washed twice with 40 mL TBS and subsequently incubated with 30–40 mL cleavage solution (a) for protein hydrazides: 2.5–5% hydrazine in TBS, 50 mM DTT, 48 h, rt; (b) for protein MesNa-thioesters: 500 mM MesNa in PBS pH 5.5, 48 h, 37 °C. The supernatant was purified by RP-HPLC (Kromasil 300-10-C4, 21.2×250 mm; 60 °C, 10 mL/min, 5%B–25%B in 10 min, 25%B–40%B in 40 min). Product containing fractions were pooled and lyophilized. Representative SDS-PAGES of the workflow are shown in Figures S2–5. Characterization of products is shown in Figures S 12, 13, 16-20, 22, 23, 26, 26.

Generation of C-terminal allylamides:

a) From Ub-thioester:

The starting material was dissolved in H_2O+0.1%TFA (better solubility under acidic conditions) at a concentration of 1.5 mg/mL and cooled at 0 °C. Allylamine or allylamine hydrochloride were added to a concentration of 500 mM. In case of the free base, no adjustment of the pH was necessary. When using allylamine hydrochloride, the pH was adjusted to 9.5 by the addition of 1 M NaOH. The reaction was stirred for 1-2 h and subsequently purified by preparative RP-HPLC (Kromasil 300-10-C4, 21.2×250 mm; 60 °C, 10 mL/min, 5%B–25%B in 10 min, 25%B–40%B in 40 min). Product containing fractions were pooled and lyophilized.

b) From Ub-hydrazide:

The hydrazide was dissolved in 6 M Gdn-HCl, 200 mM Sodium Phosphate Buffer (pH 3.0) (~ 5 mM) and cooled to -15 °C for 5 min. An azide stock solution (0.5 M $NaNO_2$ in 6 M Gdn-HCl, 200 mM Sodium Phosphate Buffer, pH 3.0) was prepared and cooled to -15 °C for 5 min. An aliquot of the $NaNO_2$ stock (10 eq.) was added to the reaction mixture which was stirred for 20 min at -15 °C. The solution was diluted to 1.5 mg/mL protein and allylamine or allylamine hydrochloride were added. In case of the free base, no adjustment of the pH was necessary. When using allylamine hydrochloride, the pH was adjusted to 9.5 by the addition of 1 M NaOH.

The reaction was stirred for 1–2 h and subsequently purified by preparative RP-HPLC (Kromasil 300-10-C4, 21.2×250 mm; 60 °C, 10 mL/min, 5%B–25%B in 10 min, 25%B–40%B in 40 min). Product containing fractions were pooled and lyophilized. Characterization of products is shown in Figure S14

PAc-Protection of Ub(KxxC)-MesNa or Ub(KxxC)-$NHNH_2$:[35]

Ub(KxxC)-MesNa or Ub(KxxC)-$NHNH_2$ was dissolved in previously degassed 6 M Gdn·HCl, 0.4 M sodium phosphate buffer, pH 7.15 at a concentration of ~0.5 mM. A stock solution of phenacylbromide (PAcBr, 0.1M in DMF) was prepared and an aliquot (2.5 eq.) was added to the reaction mixture. The reaction was stirred for 1–2 h at room temperature and monitored by LC-MS. PAc-protected Ub-thioesters were directly converted to the corresponding allylamide (see below). PAc-protected Ub-hydrazides were isolated by preparative RP-HPLC (Kromasil 300-10-C4, 21.2×250 mm; 60 °C, 10 mL/min, 5%B–25%B in 10 min, 25%B–40%B in 40 min). Product containing fractions were pooled and lyophilized. Characterization of products is shown in Figure S24.

Synthesis of Ub(KxxC(PAc))-allylamide:

a) From Ub(KxxC(PAc))-MesNa:

The crude reaction mixture of the protection reaction was diluted with H_2O to a protein concentration of 1.5 mg/mL and cooled to 0 °C. Allylamine or allylamine hydrochloride were added to a concentration of 500 mM. In case of the free base, no adjustment of the pH was necessary. When using allylamine hydrochloride, the pH was adjusted to 9.5 by the addition of 1 M NaOH. The reaction was stirred for 1–2 h and subsequently purified by preparative RP-HPLC (Kromasil 300-10-C4, 21.2×250 mm; 60 °C, 10 mL/min, 5%B–25%B in 10 min, 25%B–40%B in 40 min). Product containing fractions were pooled and lyophilized.

b) From Ub-hydrazide:

The hydrazide was dissolved in 6 M Gdn-HCl, 200 mM Sodium Phosphate Buffer (pH 3.0) (~ 5 mM) and cooled to -15 °C for 5 min. An azide stock solution (0.5 M $NaNO_2$ in 6 M Gdn-HCl, 200 mM Sodium Phosphate Buffer, pH 3.0) was prepared and cooled to -15 °C for 5 min. An aliquot of the $NaNO_2$ stock (10 eq.) was added to the reaction mixture which was stirred for 20 min at -15 °C. The solution was diluted to 1.5 mg/mL protein and allylamine or allylamine hydrochloride were added. In case of the free base, no adjustment of the pH was necessary. When using allylamine hydrochloride, the pH was adjusted to 9.5 by the addition of 1 M NaOH. The reaction was stirred for 1-2 h and subsequently purified by preparative RP-HPLC (Kromasil 300-10-C4, 21.2×250 mm; 60 °C, 10 mL/min, 5%B–25%B in 10 min, 25%B–40%B in 40 min). Product containing fractions were pooled and lyophilized.

Characterization of Ub(KxxC(PAc))-AA is shown in Figures S21, 25, 28.

Thiol-ene reaction

All buffers and solutions were degassed by purging with argon. The Ub(KxxC)-$NHNH_2$ (usually slightly less soluble than wildtype) was dissolved in 250 mM NaOAc buffer with 6 M Gdn-HCl (pH 5.5) to give a 7.5 mM solution in an 1.5 mL Eppendorf tube (for the production of di-ubiquitin, usually 4.0 mg are used). Subsequently, the ubiquitin-allylamide derivative (6 mM) was added (for the production of di-ubiquitin, usually 3.2 mg are used). The sample was centrifuged to the bottom of the vessel and equipped with a stirring bar. Ultrasonication and freeze-thaw cycles can be applied in case of incomplete dissolution. An aliquot of a TCEP stock solution in H_2O (50 mM) was added to obtain a concentration of 2 mM. The reaction mixture was incubated on ice for 5 min. Subsequently LAP (from 75 mM stock solution in H_2O) was added to obtain a concentration of 4 mM. The open Eppendorf tube was placed under an Ar- or N_2-shower and A high power LED (ThorLabs CS2010, 365 nm) was placed above (see Figure S1) and the reaction mixture was irradiated under heavy stirring at 120 mW/cm^2 for 3–5 × 15 s with 15 s break in between. The reaction mixture was diluted with 250 mM NaOAc buffer with 6 M Gdn-HCl and directly frozen until purification to prevent side reactions with initiator byproducts.

The product was isolated by semi-preparative RP-HPLC (Kromasil 300-10-C4, 10×250 mm; 60 °C, 5 mL/min, 5%B–25%B in 10 min, 25%B–40%B in 40 min). Product containing fractions were pooled and lyophilized.

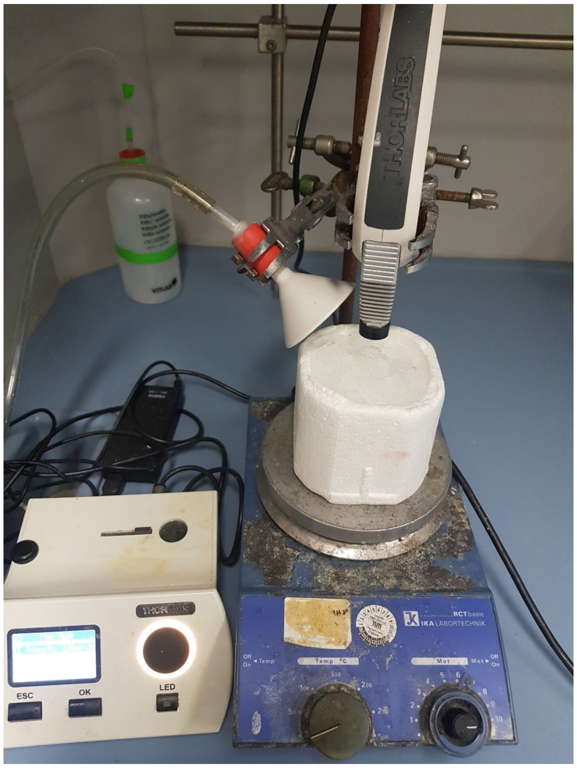

Figure S1. *Picture of the irradiation setup for thiol-ene click reactions.*

<u>**Generation of Ub building blocks by recombinant expression**</u>

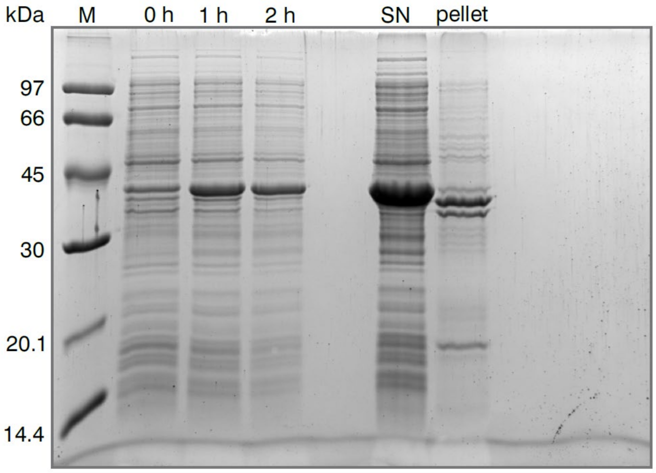

Figure S2. *Expression of Ub-Mxe-H7-CBD. SDS-PAGE from expression in BL21 DE3 Gold and lysis. Coomassie staining.* m_{calc}*(Ub(wt)-Mxe-H7-CBD) = 37623 Da.*

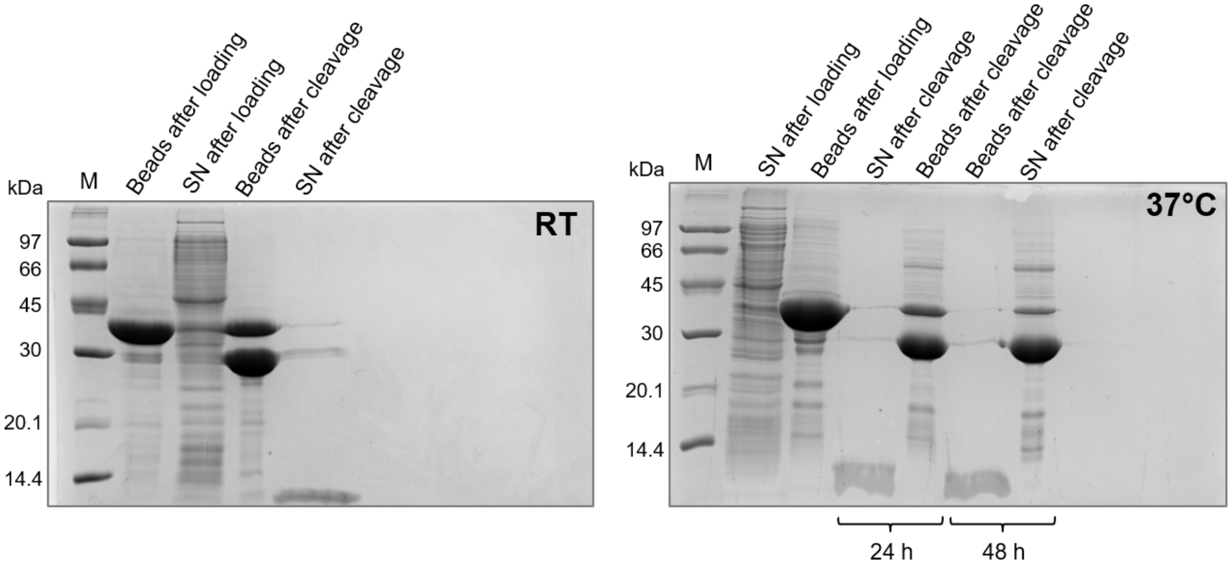

Figure S3. *Chitin beads loading and intein cleavage of Ub-Mxe-H7-CBD by MesNa (500 mM) in PBS pH 5.5 at rt (left, after 3 d) and 37 °C (right). SDS-PAGE with Coomassie staining.* m_{calc}*(Ub(wt)-Mxe-H7-CBD) = 37623 Da,* m_{calc}*(Mxe-H7-CBD) = 29076 Da,* m_{calc}*(Ub(wt)-MesNa) = 8689 Da.*

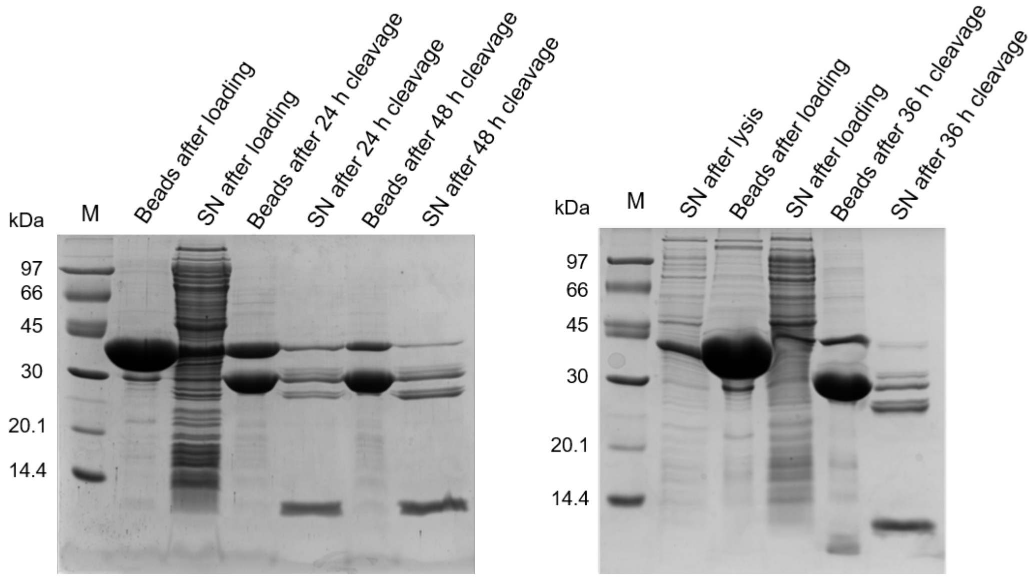

Figure S4. *Chitin beads loading and intein cleavage of Ub-Mxe-H7-CBD by hydrazine (left: 5%; right: 2.5%) in TBS pH 9.8 at room temperature. SDS-PAGE with Coomassie staining.* m_{calc}*(Ub(wt)-Mxe-H7-CBD) = 37623 Da,* m_{calc}*(Mxe-H7-CBD) = 29076 Da,* m_{calc}*(Ub(wt)-NHNH*$_2$*) = 8579 Da.*

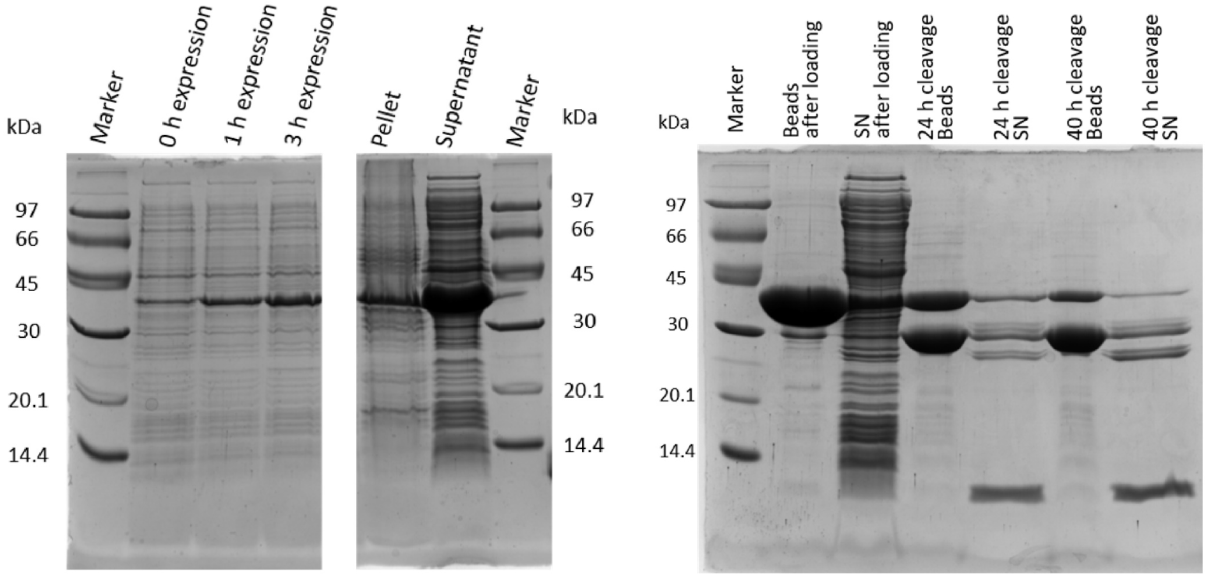

Figure S5. *left: Expression of Ub(K48C)-Mxe-H7-CBD. SDS-PAGE from expression in BL21 DE3 Gold and lysis. Coomassie staining.* m_{calc}*(Ub(K48C)-Mxe-H7-CBD) = 37598 Da. Right: Chitin beads loading and intein cleavage of Ub(K48C)-Mxe-H7-CBD by hydrazine (5%) in TBS pH 9.8 at room temperature. SDS-PAGE with Coomassie staining.* m_{calc}*(Ub(K48C)-Mxe-H7-CBD) = 37598Da,* m_{calc}*(Mxe-H7-CBD) = 29076 Da,* m_{calc}*(Ub(K48C)-NHNH*$_2$*) = 8554 Da.*

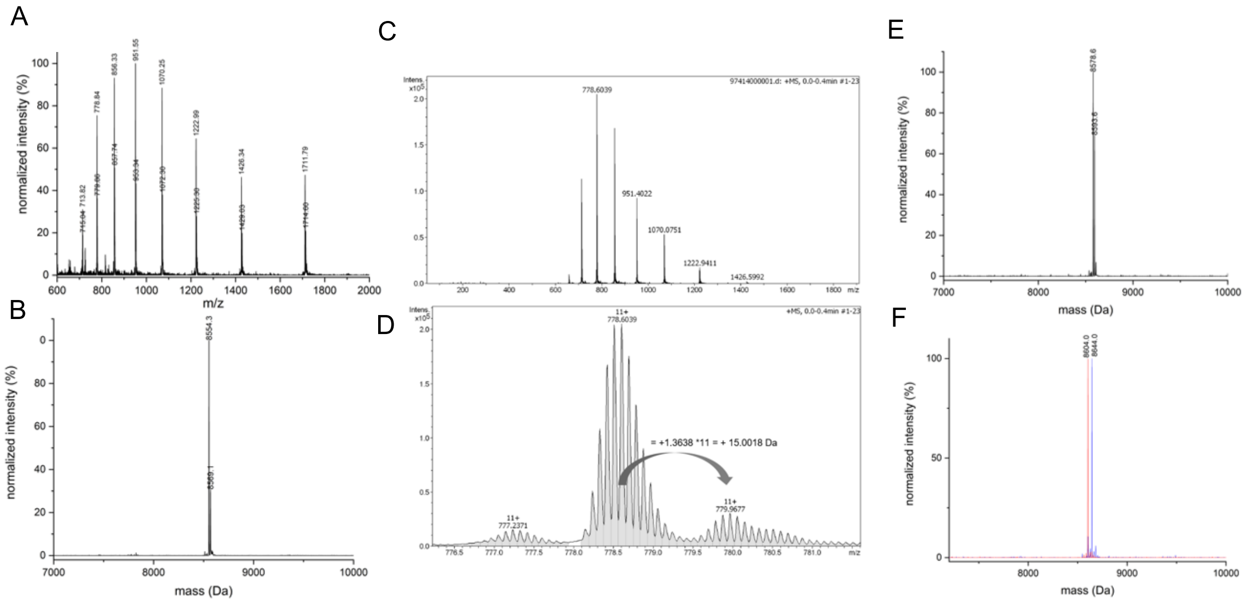

Figure S6. Dihydrazide byproduct during intein clevage with N_2H_4/DTT. *a+b) mass spectrum (ES+) and deconvoluted mass spectrum of a fraction of Ub(K48C)-$NHNH_2$ after HPLC purification; c+d) HR-MS (ES+) of purified Ub(K48C)-$NHNH_2$ and zoom-in to 11+ peak of product and sideproduct. m_{calc}(Ub(K48C)-$NHNH_2$ = 8553.87 Da; m_{calc}(Ub(K48C)-bishydrazide) = 8568.88 Da.e) deconvoluted mass spectrum (ES+) of an early eluting fraction of Ub(wt)-$NHNH_2$. m_{calc}(Ub(wt)-$NHNH_2$ = 8578.90 Da ; m_{calc}(Ub(wt)-bishydrazide) = 8593.91 Da; f) deconvoluted mass spectra (ES+) of isolated Ub(wt)-AA (red) and isolated sideproduct Ub(wt)-bisAA (blue). m_{calc}(Ub(wt)-AA) = 8603.92 Da; m_{calc}(Ub(wt)-bisAA) = 8643.98 Da.*

Table S1. *Radical initiators for the TEC reaction between Ub(wt)-allylamide and Ub(K48C)-$NHNH_2$ evaluated in this work. (see also Figures S7-11)*

Initiator	Conditions	Result
LAP<br>[Lithium phenyl-2,4,6-trimethylbenzoylphosphinate]	6 mM allylamide<br>7.5 mM thiol<br>2 mM TCEP, 4 mM initiator<br>250mM NaOAc, 6M GdnHCl pH5.5<br>0 °C, 365 nm, 3x 15 s	di-Ubiquitin as major product,<br>Ub-phosphinate as sideproduct
Irgacure2959<br>[2-Hydroxy-4′-(2-hydroxy ethoxy)-2-methylpropiophenon]	6 mM allylamide<br>7.5 mM thiol<br>2 mM TCEP, 16 mM initiator<br>250mM NaOAc, 6M GdnHCl pH5.5<br>0 °C, 365 nm, 20 min	Small amount of di-Ubiquitin formed, mainly starting material
$NaBEt_4$	6 mM allylamide<br>7.5 mM thiol<br>0.5 mM TCEP, 20 mM initiator<br>250mM NaOAc, 6M GdnHCl pH5.5<br>rt, 120 min	Small amount of Ubiquitin disulfide formed, consumption of Ub(K48C)-$NHNH_2$ to unidentified sideproducts
VA-044<br>[2,2'-Azobis[2-(2-imidazolin-2-yl)propane]dihydrochloride]	6 mM allylamide<br>7.5 mM thiol<br>2 mM TCEP, 16 mM initiator<br>250mM NaOAc, 6M GdnHCl pH5.5<br>50 °C, 140 min	Consumption of Ub(K48C)-NHNH2 by disulfide formation
V-50<br>[2,2'-Azobis(2-methylpropionamidin) dihydrochloride]	6 mM allylamide<br>7.5 mM thiol<br>2 mM TCEP, 16 mM initiator<br>250mM NaOAc, 6M GdnHCl pH5.5<br>50 °C, 140 min	No reaction

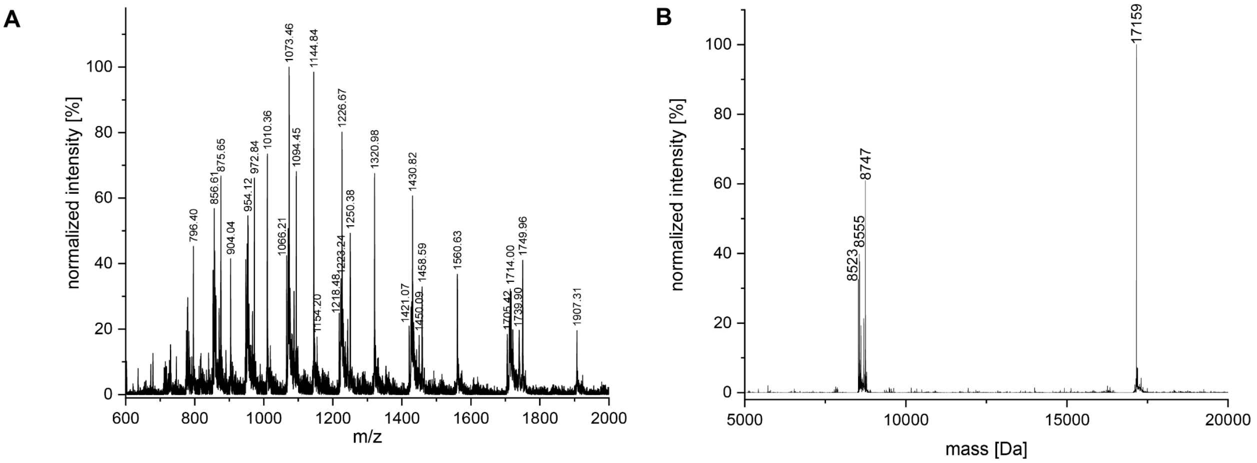

Figure S7. *Thiol-ene reaction initiated by LAP (see Table S1). Mass spectrum (A) and deconvoluted mass spectrum (B) of the crude reaction. Calculated masses: Ub(wt)-AA: 8604 Da, Ub(K48C)-NHNH$_2$: 5554 Da, diUb(48)-NHNH$_2$: 17158 Da, Desulfurized Ub(K48C)-NHNH$_2$: 8522 Da, Ub(wt)-AA-phosphinate: 8746 Da.*

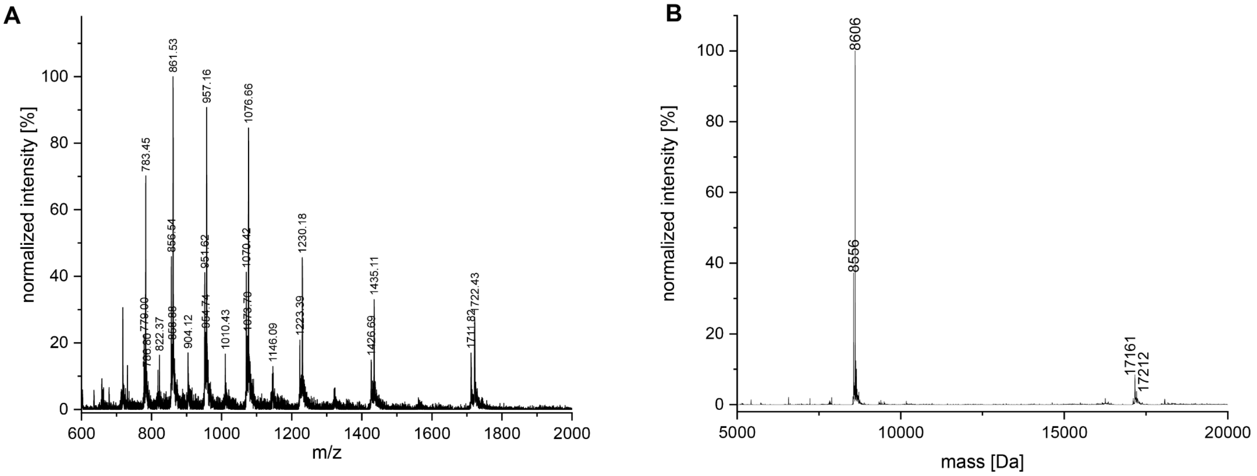

Figure S8. *Thiol-ene reaction initiated by Irgacure 2959 (see Table S1). Mass spectrum (A) and deconvoluted mass spectrum (B) of the crude reaction. Calculated masses: Ub(wt)-AA: 8604 Da, Ub(K48C)-NHNH$_2$: 5554 Da, diUb(48)-NHNH$_2$: 17158 Da. Unidentified product: 17212 Da.*

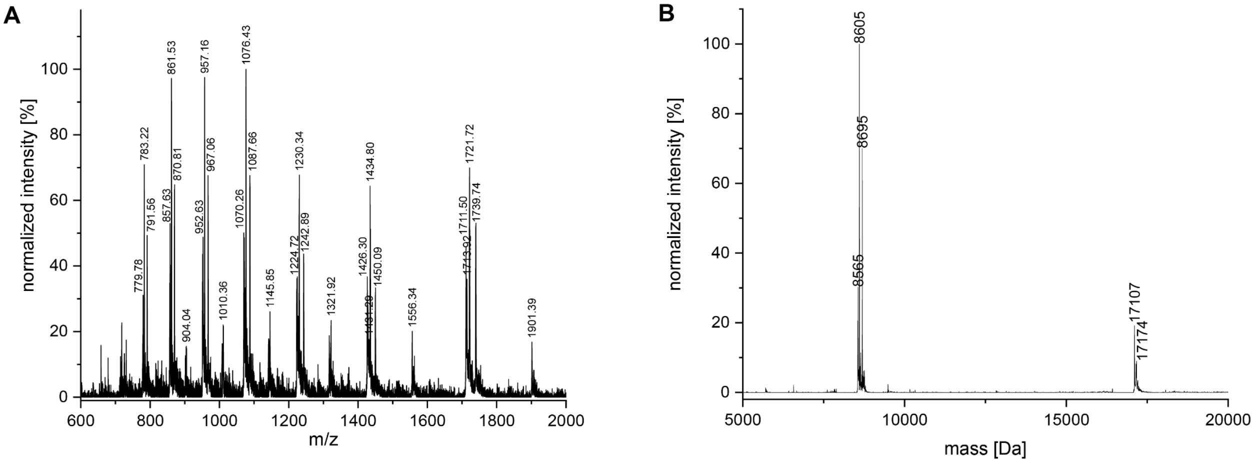

Figure S9. *Thiol-ene reaction initiated by NaBEt$_4$ (see Table S1). Mass spectrum (A) and deconvoluted mass spectrum (B) of the crude reaction. Calculated masses: Ub(wt)-AA: 8604 Da, Ub(K48C)-NHNH$_2$: 5554 Da, diUb(48)-NHNH$_2$: 17158 Da, Ub(K48C)-NHNH$_2$-disulfide: 17108 Da.*

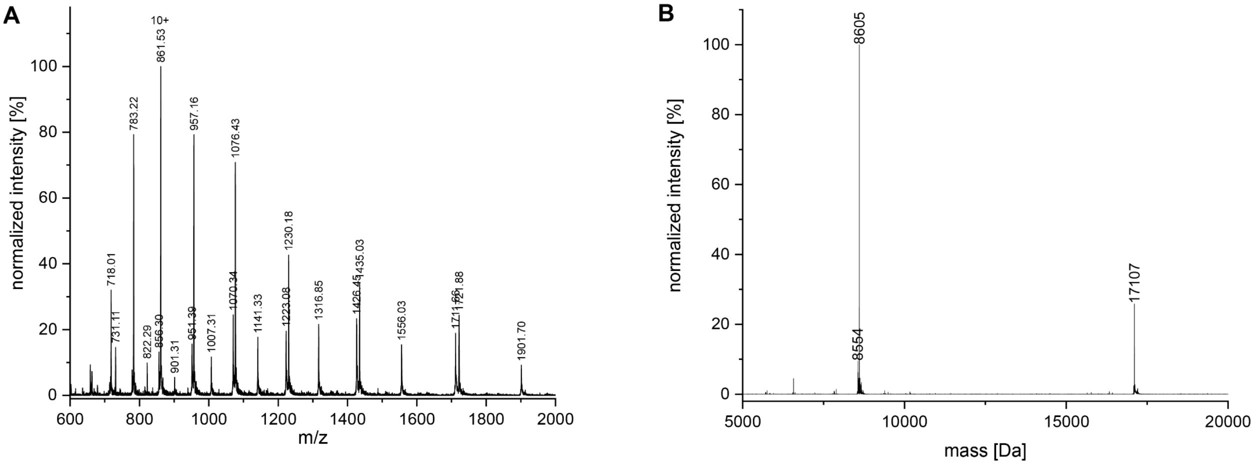

Figure S10. *Thiol-ene reaction initiated by VA-044 (see Table S1). Mass spectrum (A) and deconvoluted mass spectrum (B) of the crude reaction. Calculated masses: Ub(wt)-AA: 8604 Da, Ub(K48C)-NHNH$_2$: 5554 Da, diUb(48)-NHNH$_2$: 17158 Da, Ub(K48C)-NHNH$_2$-disulfide: 17108 Da.*

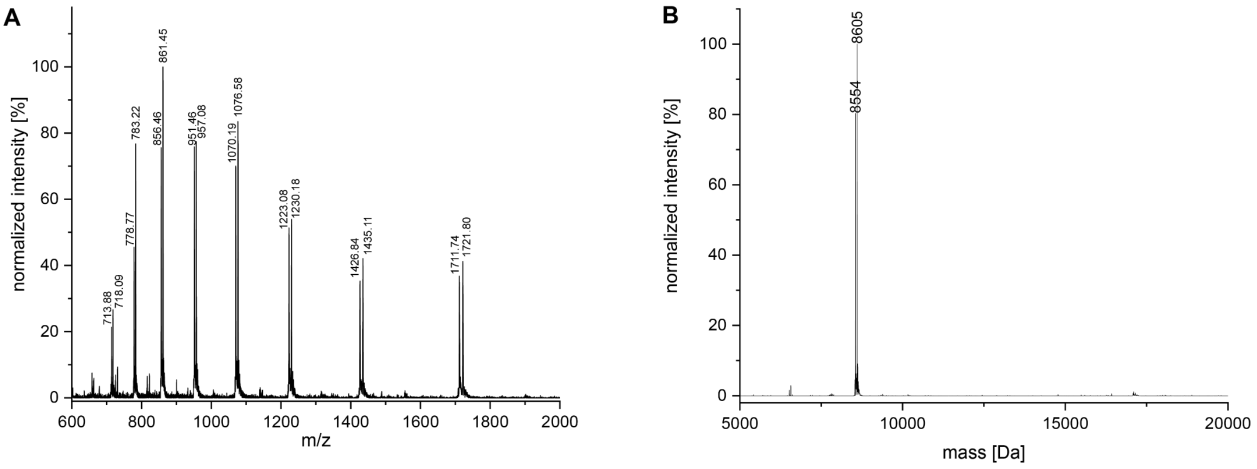

Figure S11. *Thiol-ene reaction initiated by V-50 (see Table S1). Mass spectrum (A) and deconvoluted mass spectrum (B) of the crude reaction. Calculated masses: Ub(wt)-AA: 8604 Da, Ub(K48C)-$NHNH_2$: 5554 Da, diUb(48)-$NHNH_2$: 17158 Da, Ub(K48C)-$NHNH_2$-disulfide: 17108 Da.*

Characterization of mono-ubiquitin building blocks

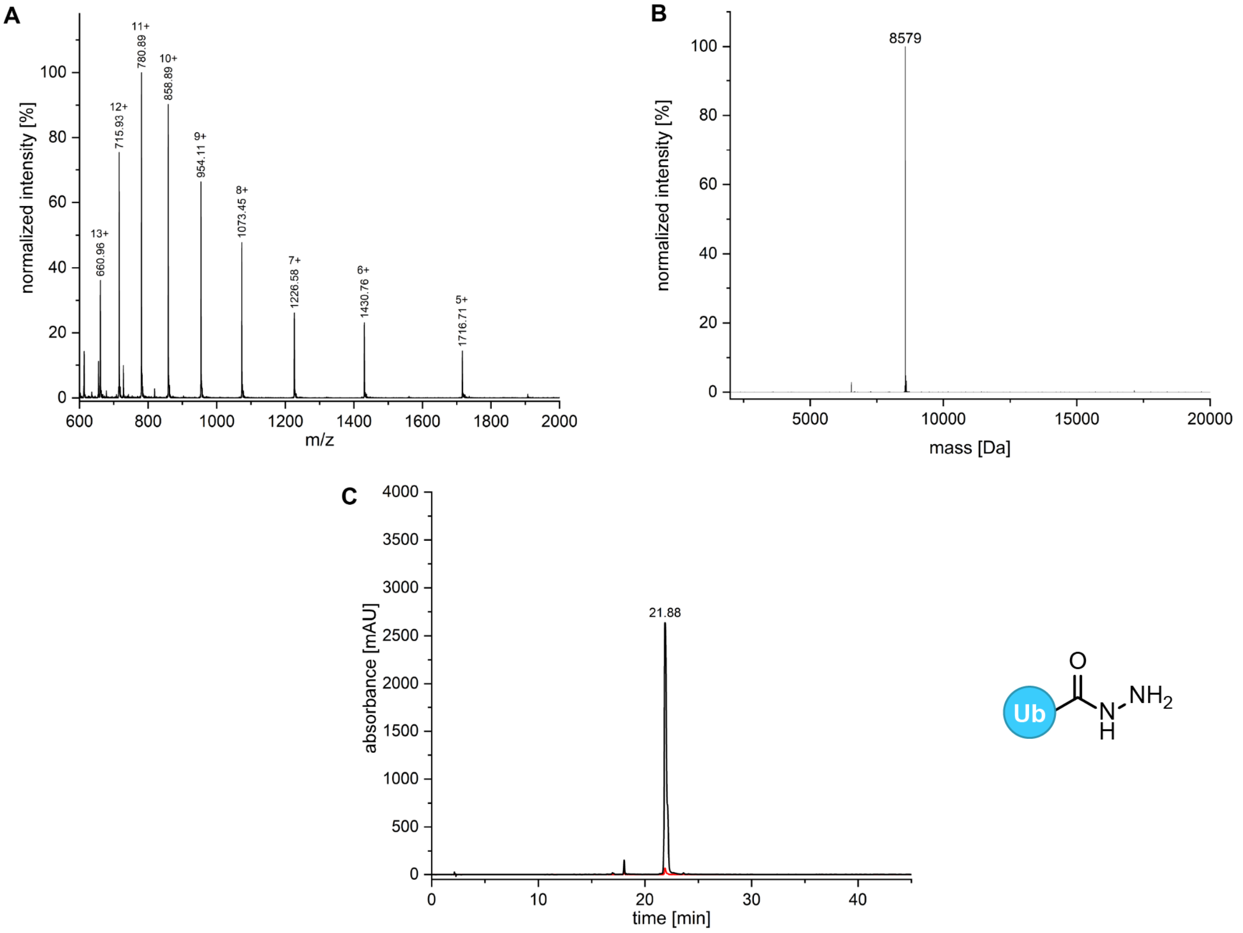

Figure S12. *Characterization of Ub(wt)-NHNH$_2$ by ESI (pos.) mass spectrometry (A) with deconvoluted mass spectrum B and analytical RP-HPLC (C; black: 214 nm, red: 280 nm). m_{calc} = 8578.90 Da.*

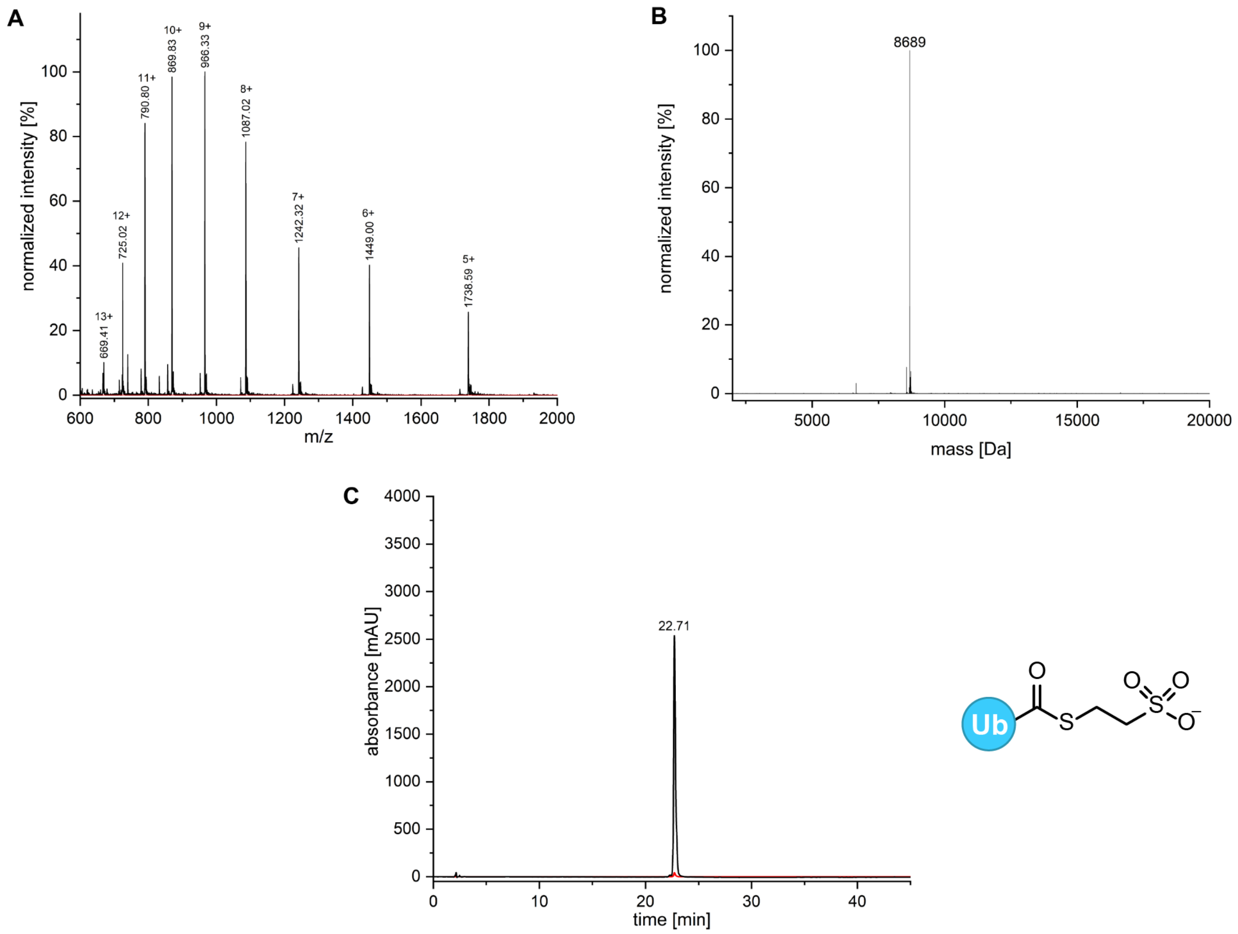

Figure S13. *Characterization of Ub(wt)-MesNa by ESI (pos.) mass spectrometry (A) with deconvoluted mass spectrum B and analytical RP-HPLC (C; black: 214 nm, red: 280 nm).* m_{calc} *= 8689.04 Da.*

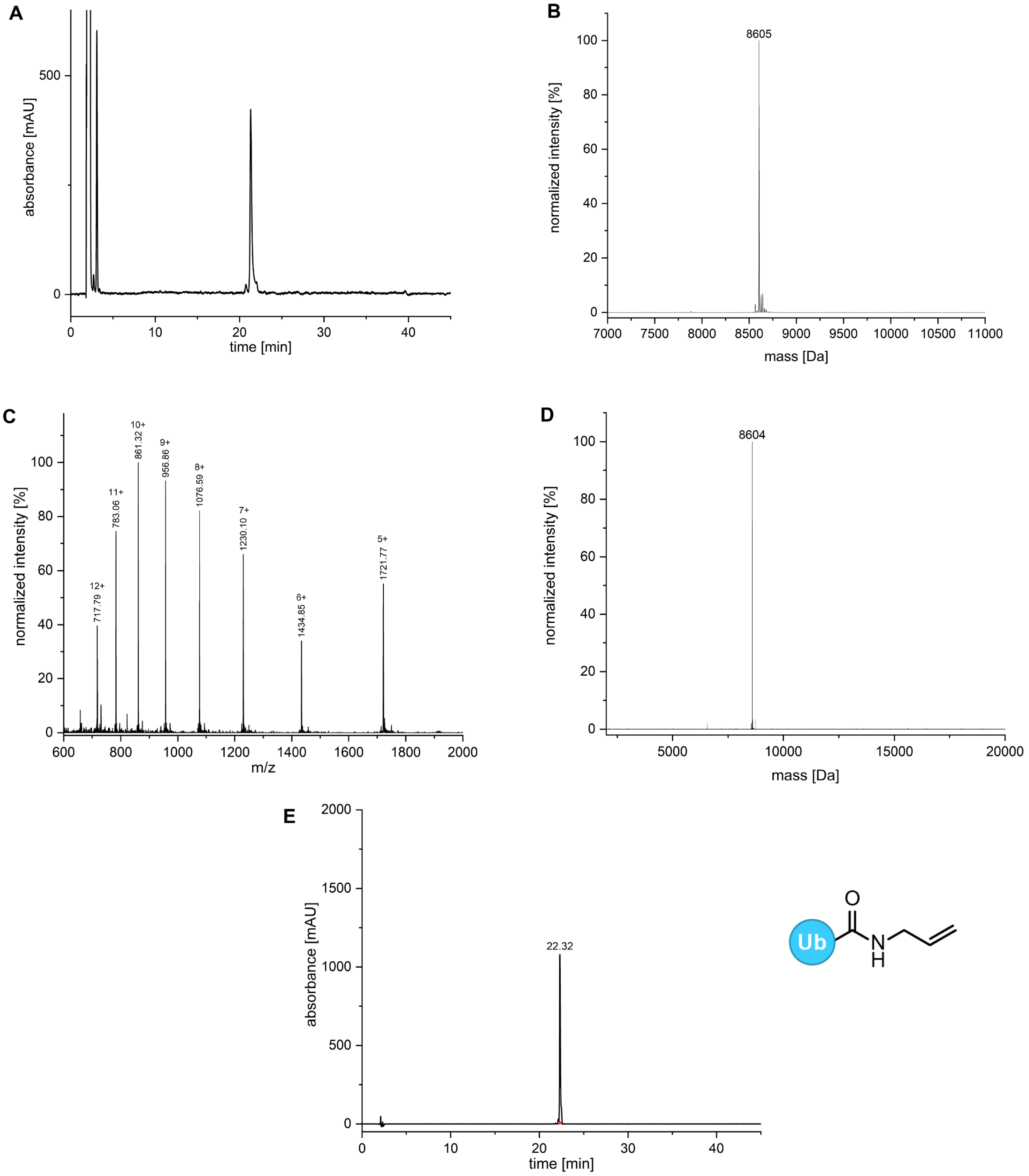

Figure S14. *Synthesis of Ub(wt)-AA. Crude reaction analysis of the aminolysis of Ub(wt)-AA after oxidative activation (A: HPLC chromatogram (214 nm) and B: deconvoluted mass spectrum). Characterization of Ub(wt)-AA by ESI (pos.) mass spectrometry (C) with deconvoluted mass spectrum D and analytical RP-HPLC (E; black: 214 nm, red: 280 nm).* m_{calc} *= 8603.92 Da.*

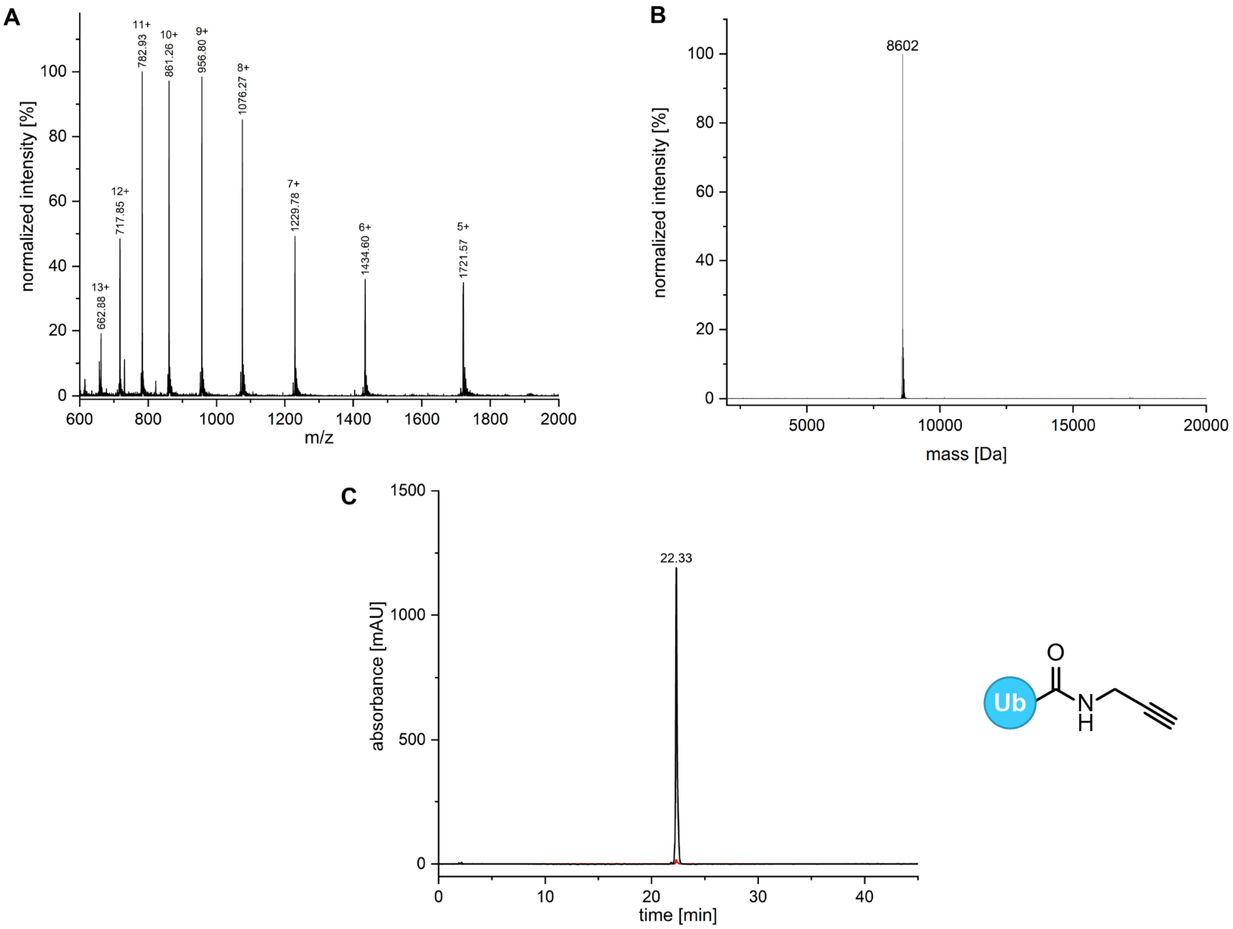

Figure S15. *Characterization of Ub(wt)-propargylamide by ESI (pos.) mass spectrometry (A) with deconvoluted mass spectrum B and analytical RP-HPLC (C; black: 214 nm, red: 280 nm). m_{calc} = 8601.94 Da.*

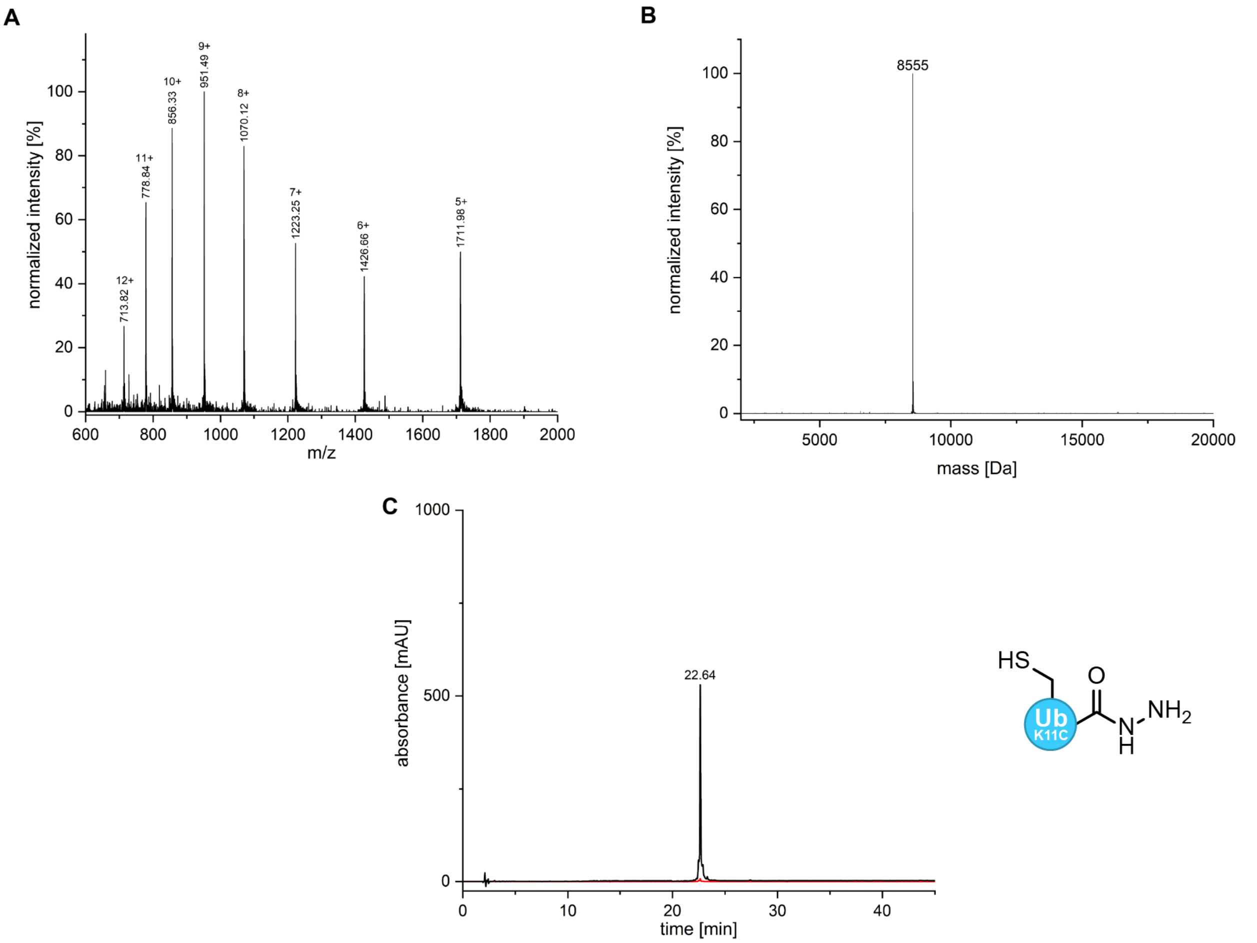

Figure S16. *Characterization of Ub(K11C)-NHNH$_2$ by ESI (pos.) mass spectrometry (A) with deconvoluted mass spectrum B and analytical RP-HPLC (C; black: 214 nm, red: 280 nm). m_{calc} = 8553.87 Da.*

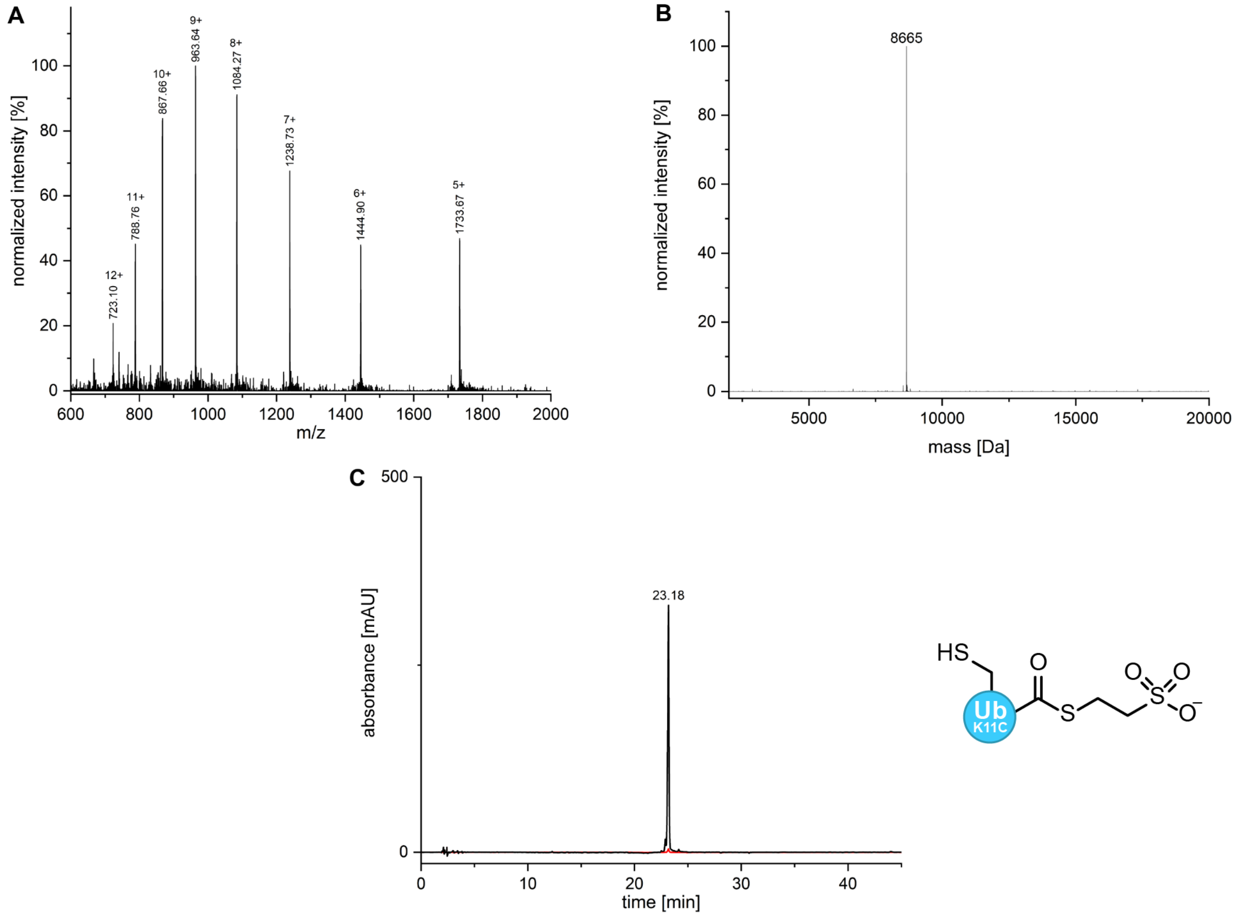

Figure S17. *Characterization of Ub(K11C)-MesNa by ESI (pos.) mass spectrometry (A) with deconvoluted mass spectrum B and analytical RP-HPLC (C; black: 214 nm, red: 280 nm). m_{calc} = 8664.01 Da.*

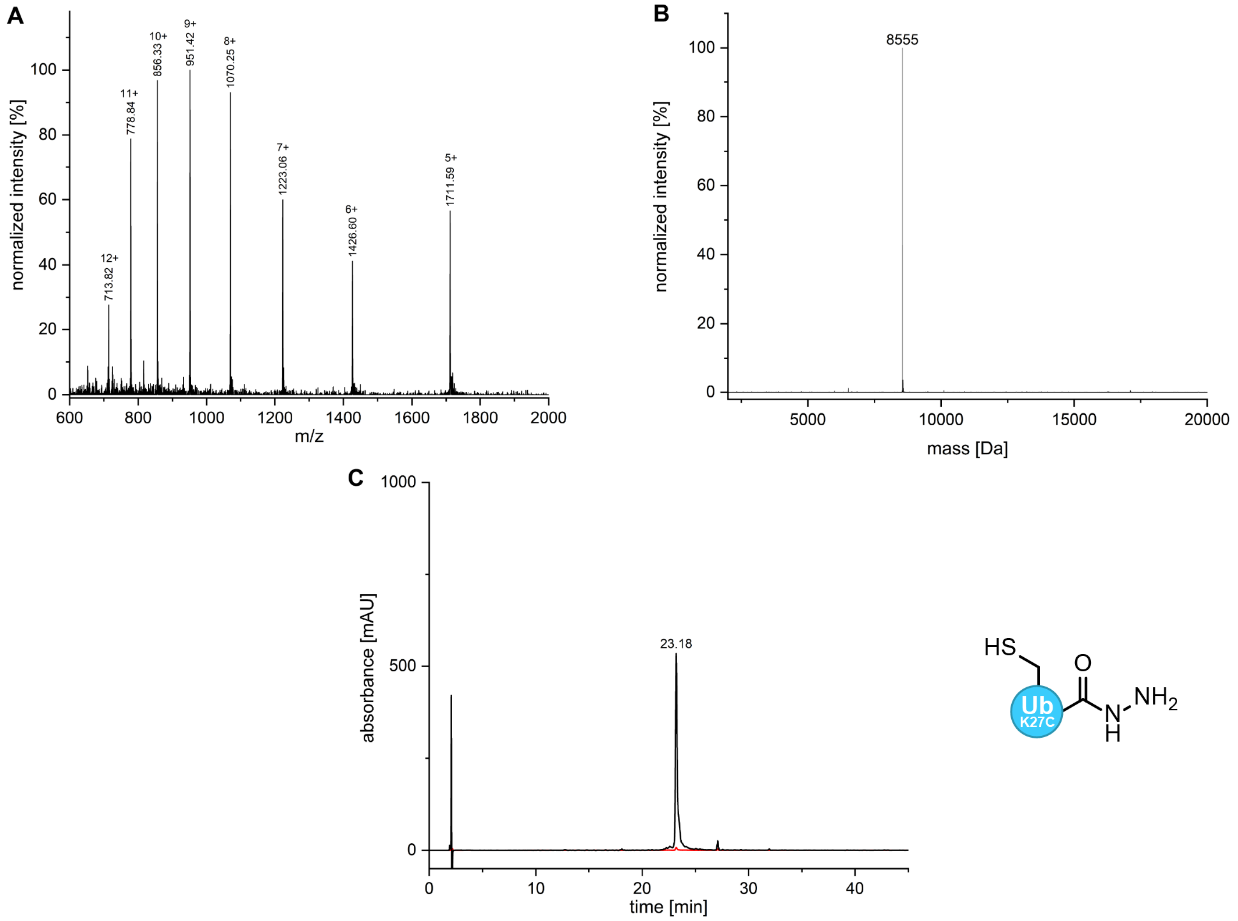

Figure S18. *Characterization of Ub(K27C)-NHNH$_2$ by ESI (pos.) mass spectrometry (A) with deconvoluted mass spectrum B and analytical RP-HPLC (C; black: 214 nm, red: 280 nm). m_{calc} = 8553.87 Da.*

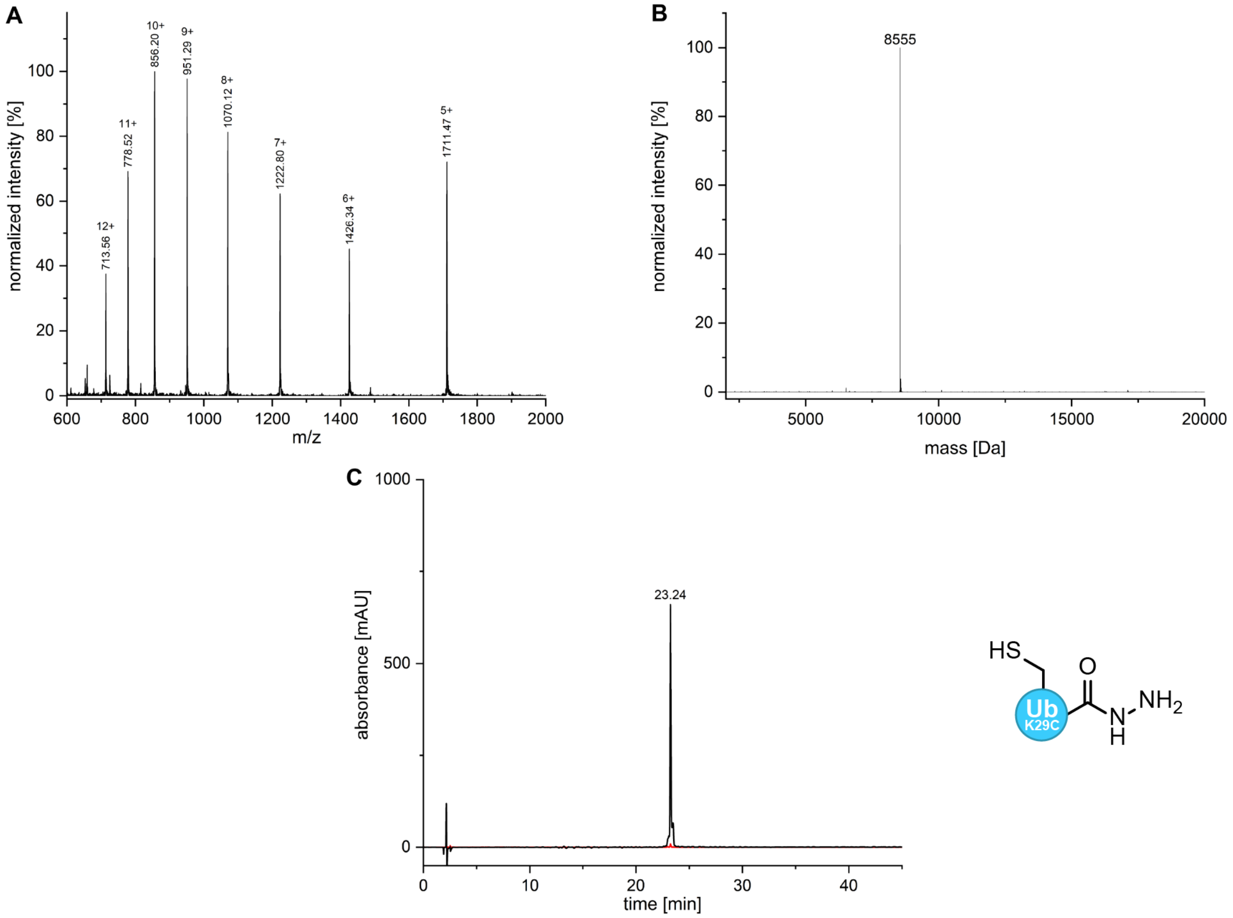

Figure S19. *Characterization of Ub(K29C)-NHNH$_2$ by ESI (pos.) mass spectrometry (A) with deconvoluted mass spectrum B and analytical RP-HPLC (C; black: 214 nm, red: 280 nm). m_{calc} = 8553.87 Da.*

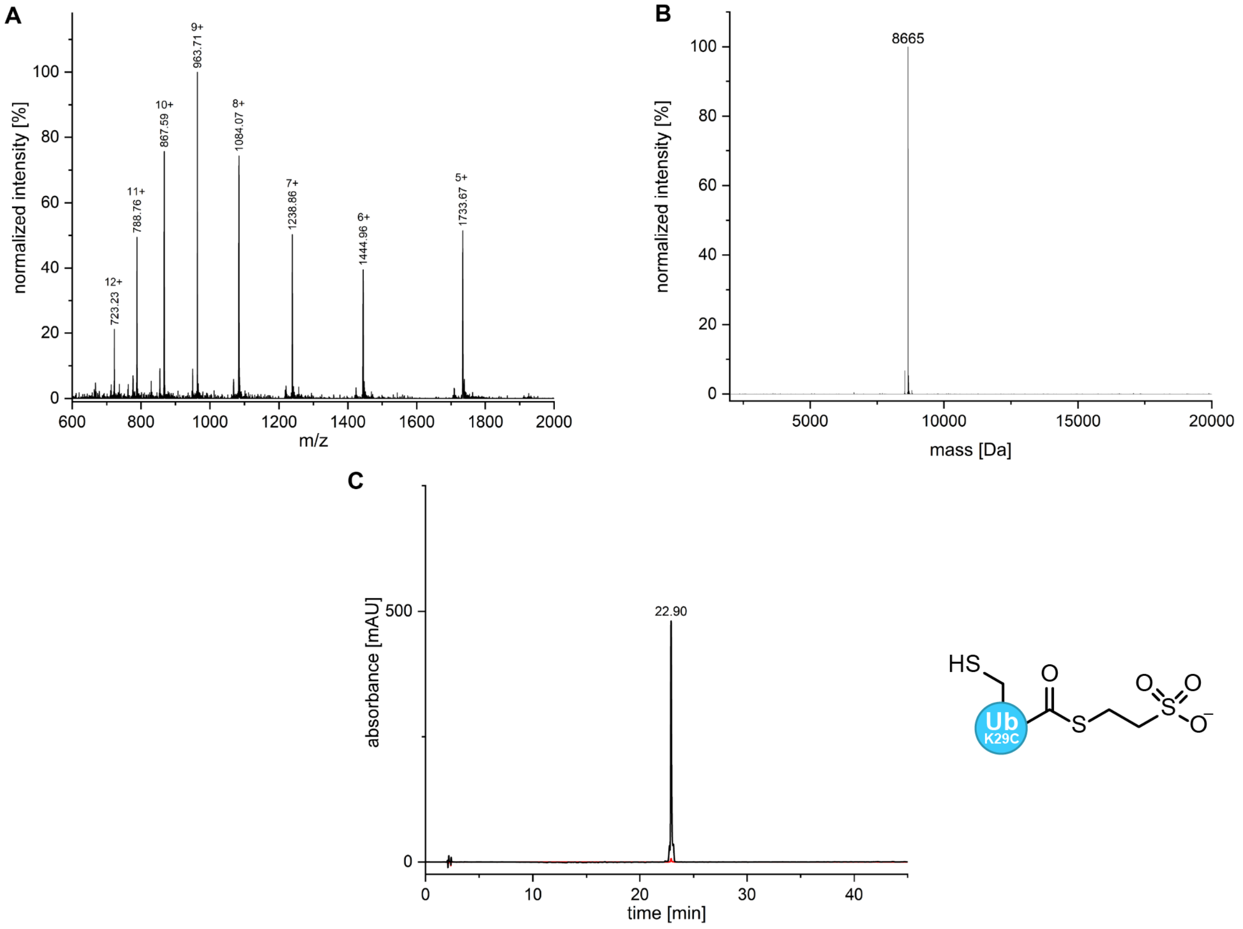

Figure S20. *Characterization of Ub(K29C)-MesNa by ESI (pos.) mass spectrometry (A) with deconvoluted mass spectrum B and analytical RP-HPLC (C; black: 214 nm, red: 280 nm).* m_{calc} *= 8664.01 Da.*

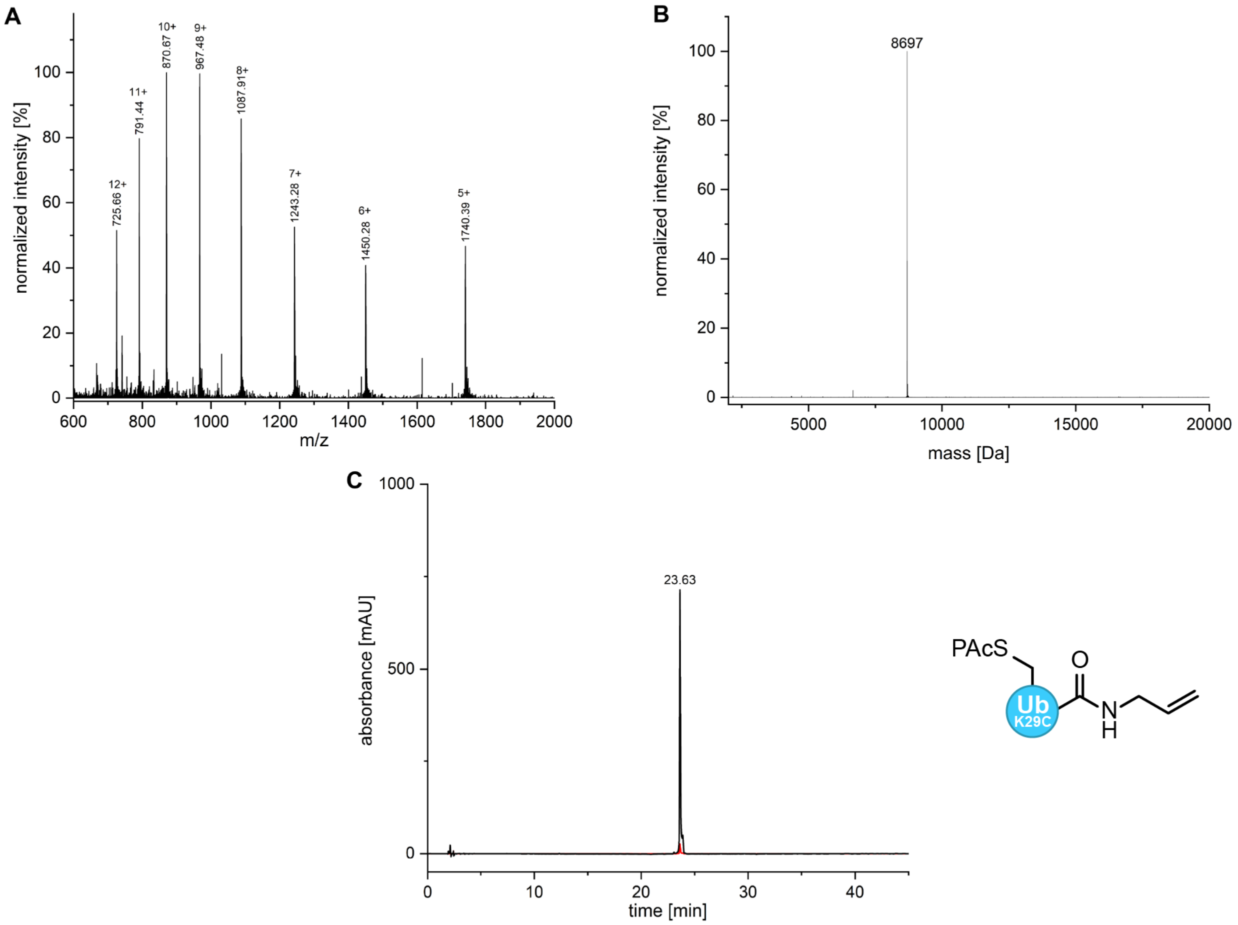

Figure S21. *Characterization of Ub(K29C(PAc))-AA by ESI (pos.) mass spectrometry (A) with deconvoluted mass spectrum B and analytical RP-HPLC (C; black: 214 nm, red: 280 nm).* m_{calc} *= 8697.06 Da.*

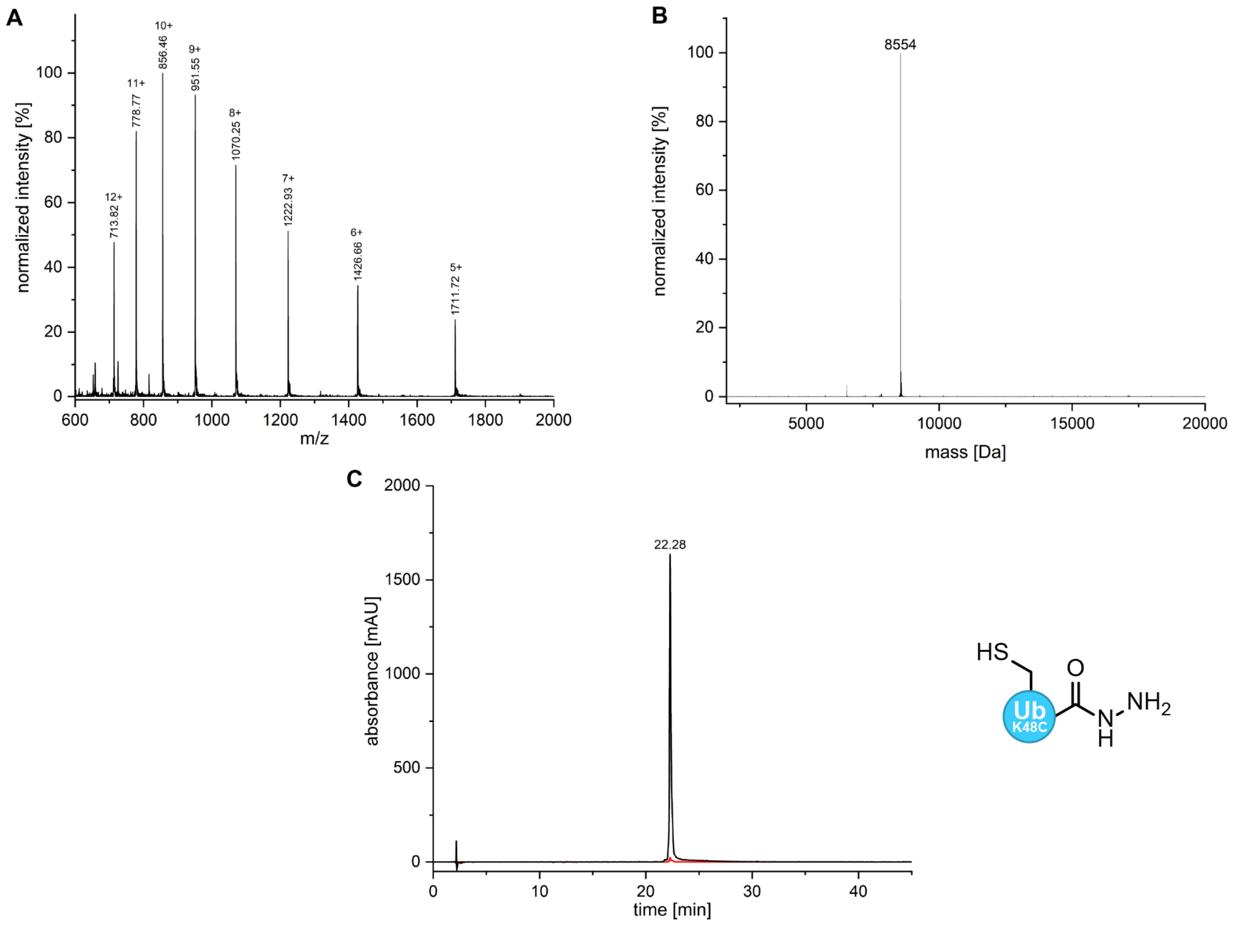

Figure S22. *Characterization of Ub(K48C)-NHNH₂ by ESI (pos.) mass spectrometry (A) with deconvoluted mass spectrum B and analytical RP-HPLC (C; black: 214 nm, red: 280 nm).* m_{calc} *= 8553.87 Da.*

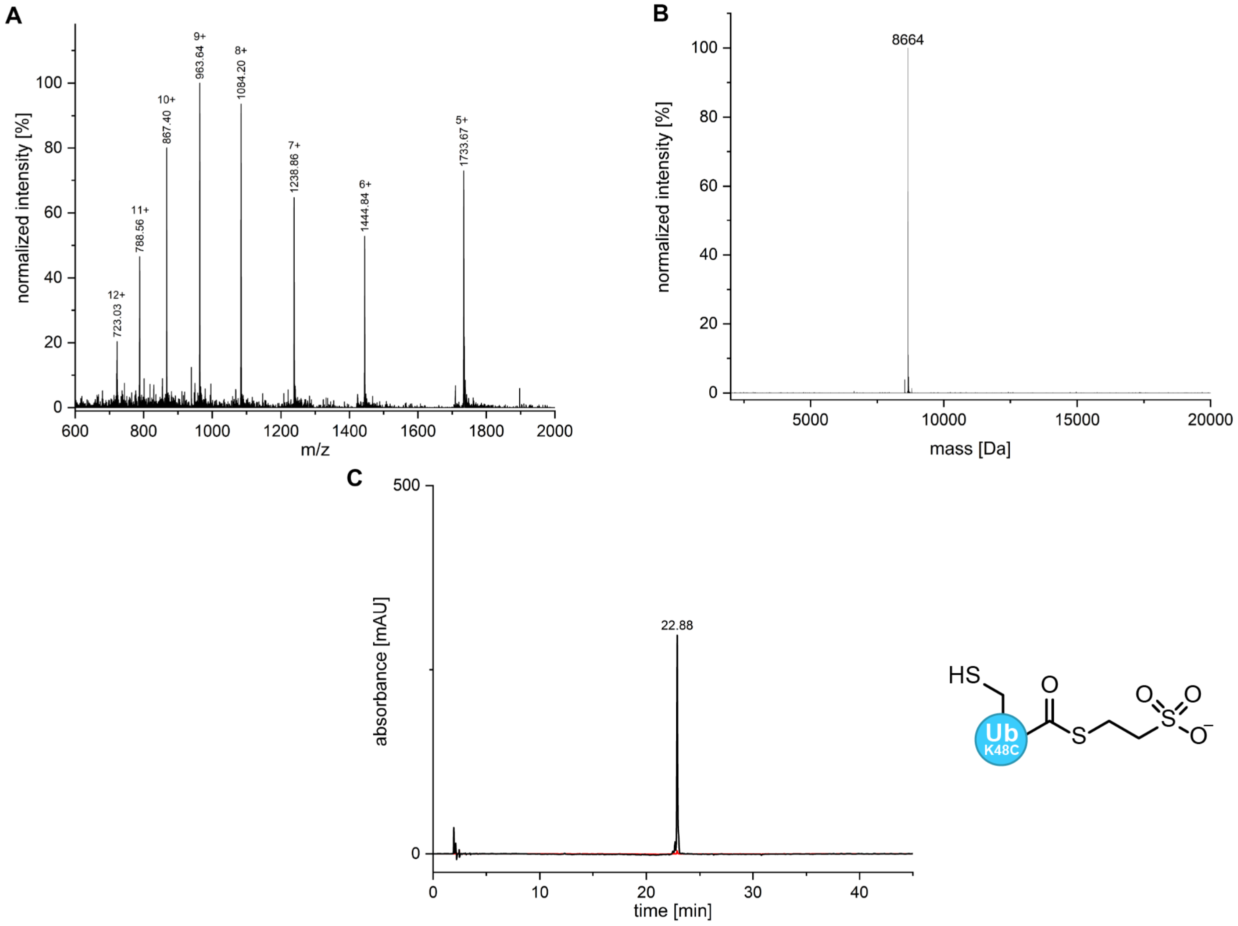

Figure S23. *Characterization of Ub(K48C)-MesNa by ESI (pos.) mass spectrometry (A) with deconvoluted mass spectrum B and analytical RP-HPLC (C; black: 214 nm, red: 280 nm).* m_{calc} *= 8664.01 Da.*

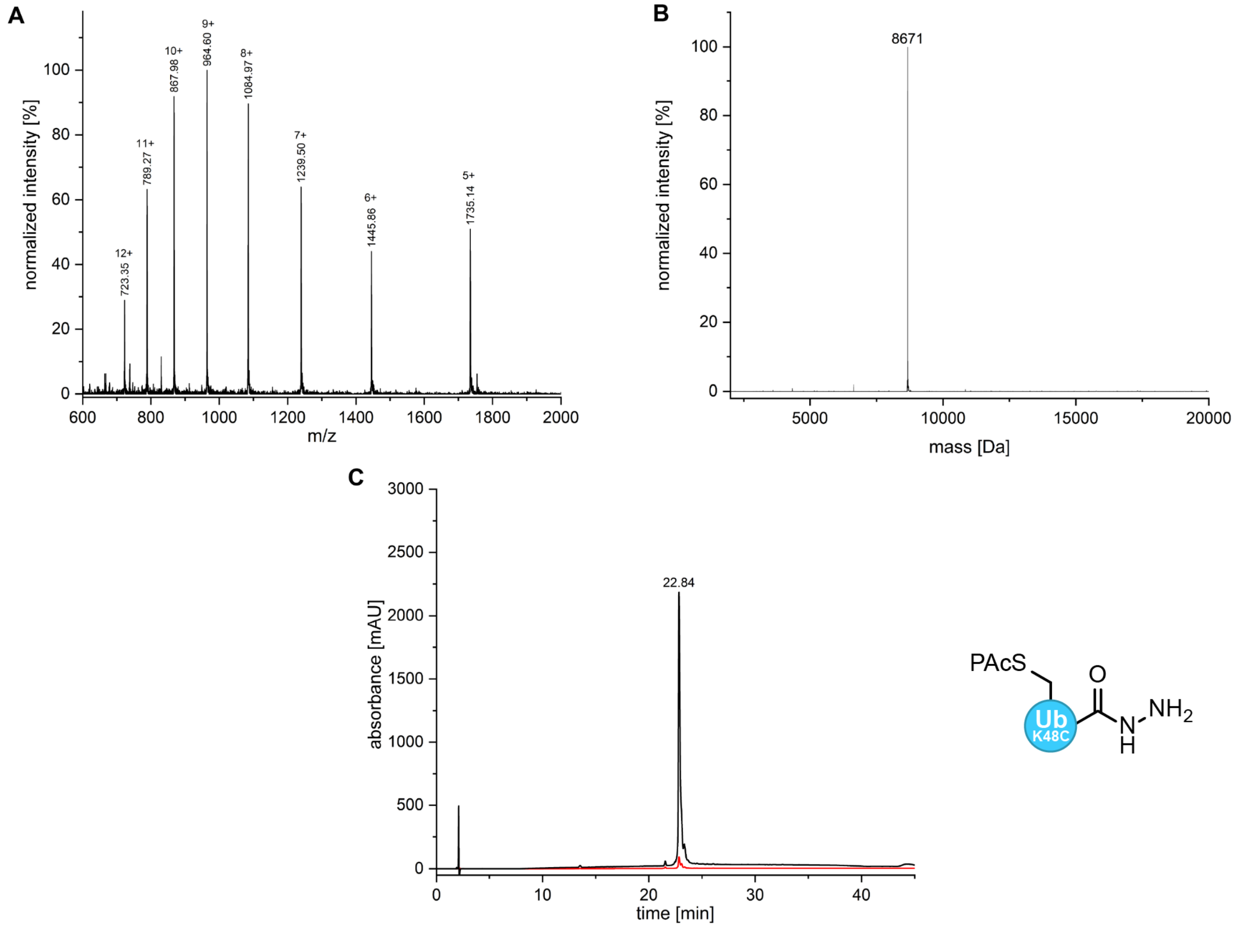

Figure S24. *Characterization of Ub(K48C(PAc))-NHNH₂ by ESI (pos.) mass spectrometry (A) with deconvoluted mass spectrum B and analytical RP-HPLC (C; black: 214 nm, red: 280 nm).* m_{calc} *= 8672.01 Da.*

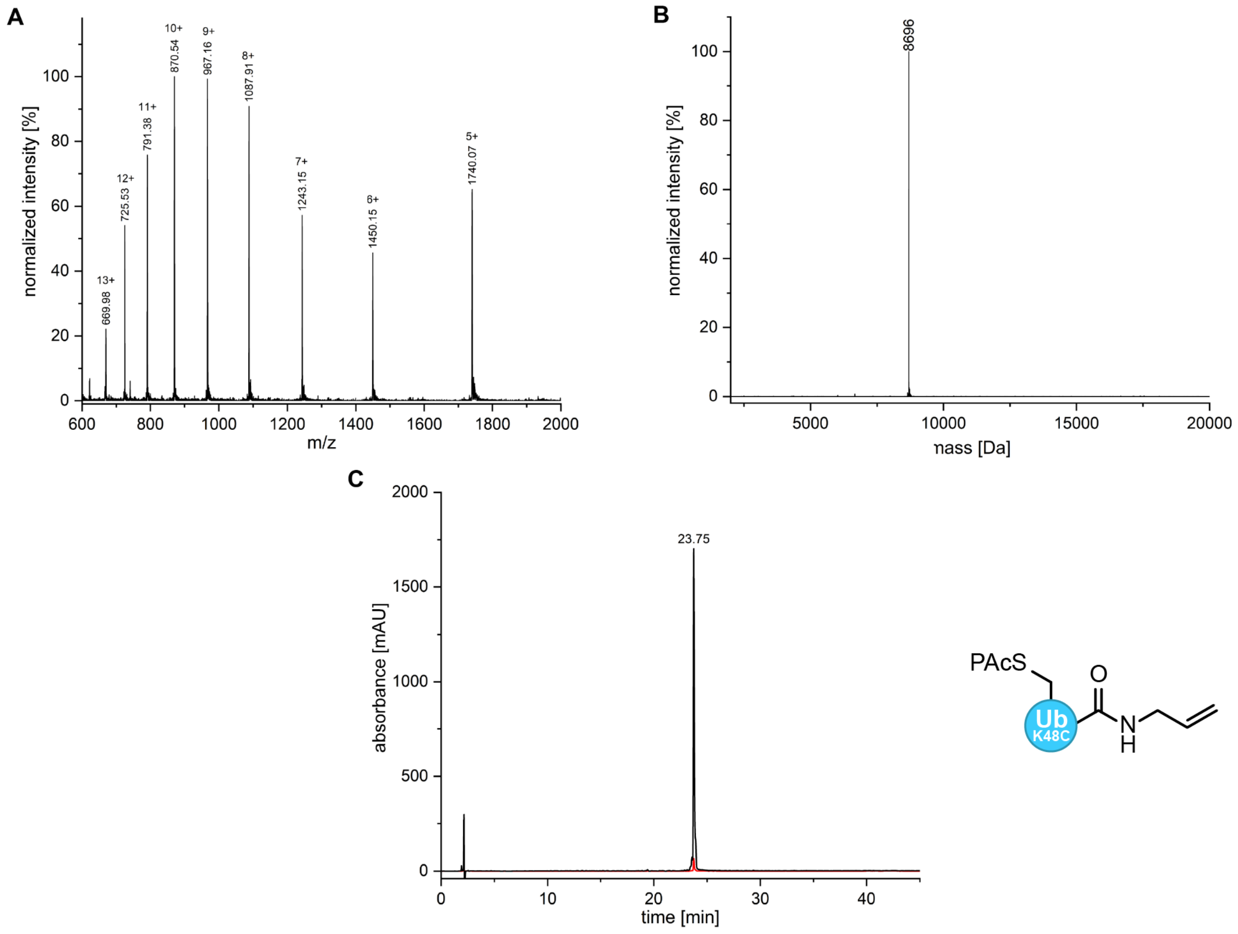

Figure S25. *Characterization of Ub(K48C(PAc))-AA by ESI (pos.) mass spectrometry (A) with deconvoluted mass spectrum B and analytical RP-HPLC (C; black: 214 nm, red: 280 nm).* m_{calc} *= 8697.06 Da.*

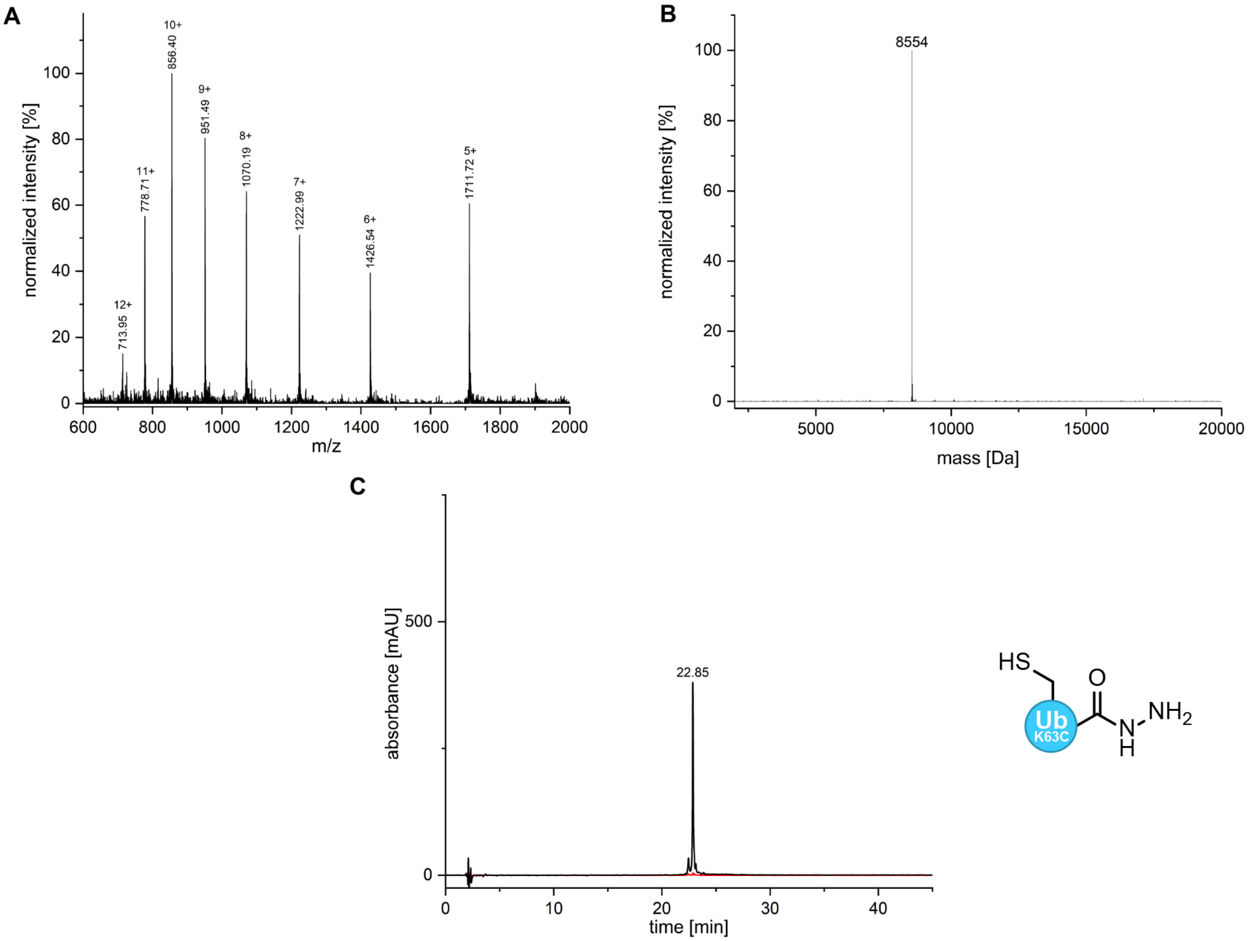

Figure S26. *Characterization of Ub(K63C)-NHNH$_2$ by ESI (pos.) mass spectrometry (A) with deconvoluted mass spectrum B and analytical RP-HPLC (C; black: 214 nm, red: 280 nm). m_{calc} = 8553.87 Da.*

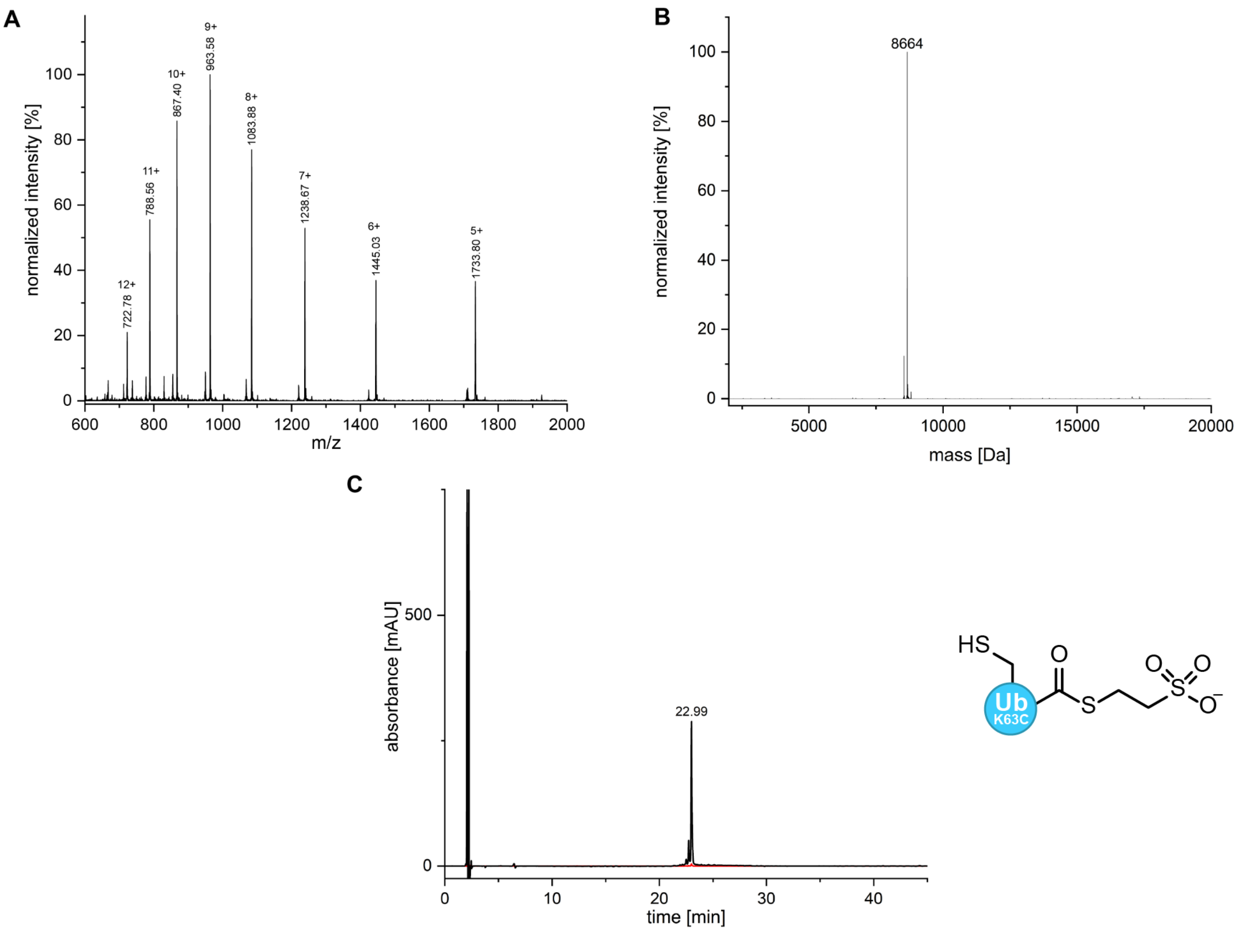

Figure S27. *Characterization of Ub(K63C)-MesNa by ESI (pos.) mass spectrometry (A) with deconvoluted mass spectrum B and analytical RP-HPLC (C; black: 214 nm, red: 280 nm).* m_{calc} *= 8664.01 Da.*

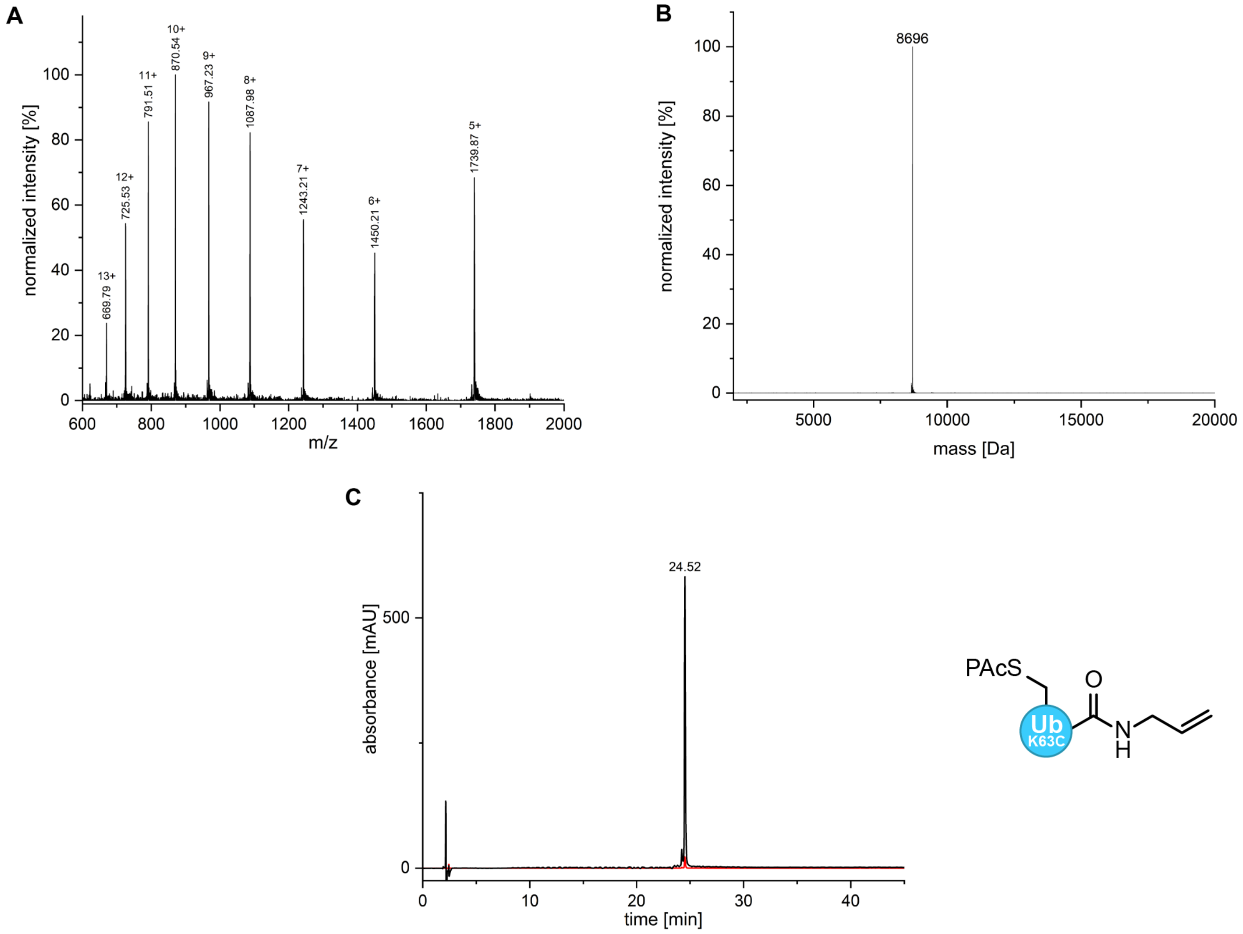

__Figure S28.__ Characterization of Ub(K63C(PAc))-AA by ESI (pos.) mass spectrometry (A) with deconvoluted mass spectrum B and analytical RP-HPLC (C; black: 214 nm, red: 280 nm). m_{calc} = 8697.06 Da.

Generation of oligo-ubiquitin chains

Characterization of di-ubiquitin motifs

Table S2. *Average isolated amounts of di-ubiquitins per reaction starting with 3.2–3.4 mg Ub(wt)-AA.*

Compound	average isolated amount/ reaction [mg]	average yield [%]
diUb(27)-$NHNH_2$	1.5	23
diUb(29)-$NHNH_2$	1.3	20
diUb(48)-$NHNH_2$	1.7	27
diUb(63)-$NHNH_2$	1.6	24

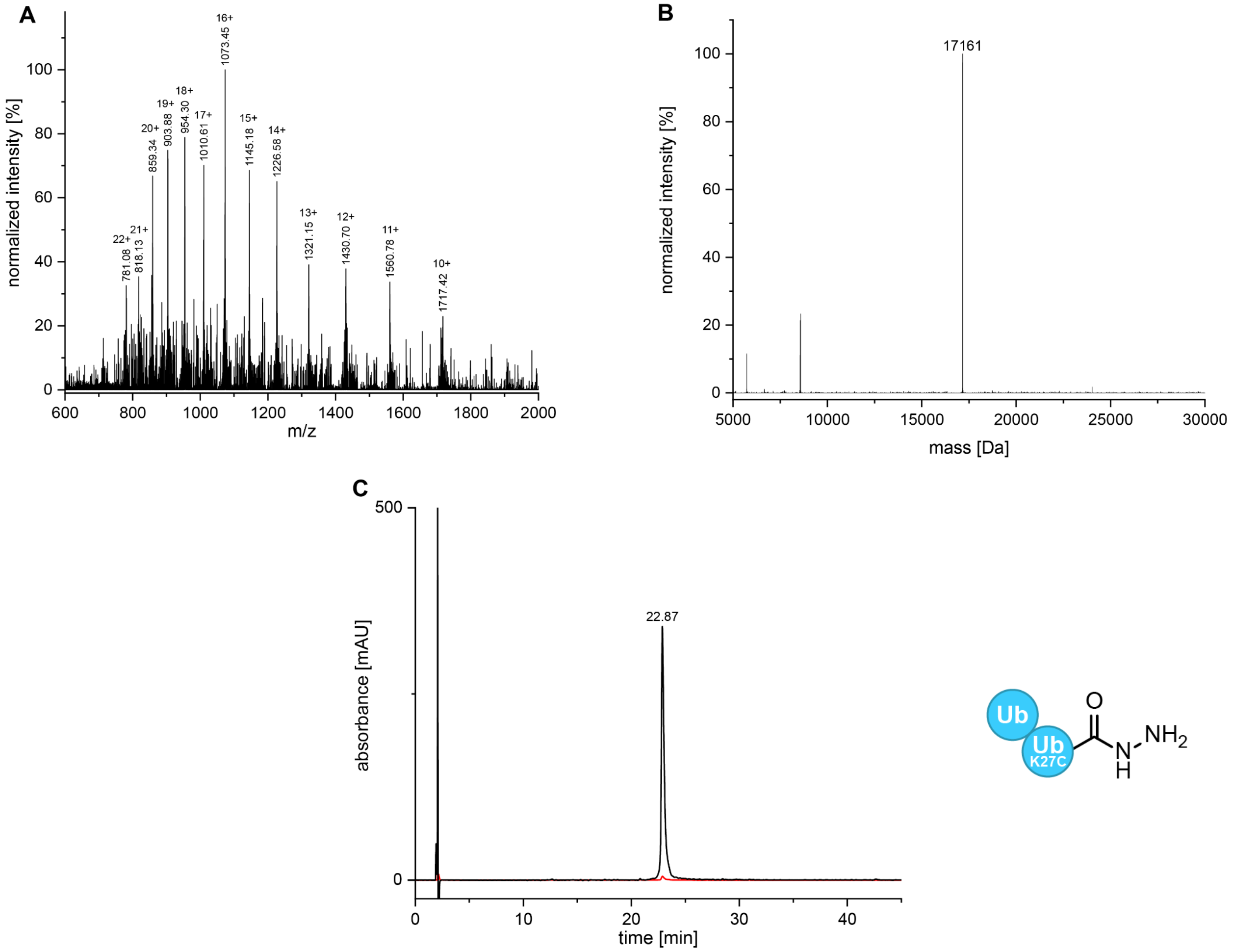

Figure S29. *Characterization of diUb(27)-$NHNH_2$ by ESI (pos.) mass spectrometry (A) with deconvoluted mass spectrum B and analytical RP-HPLC (C; black: 214 nm, red: 280 nm). m_{calc} = 17157.79 Da.*

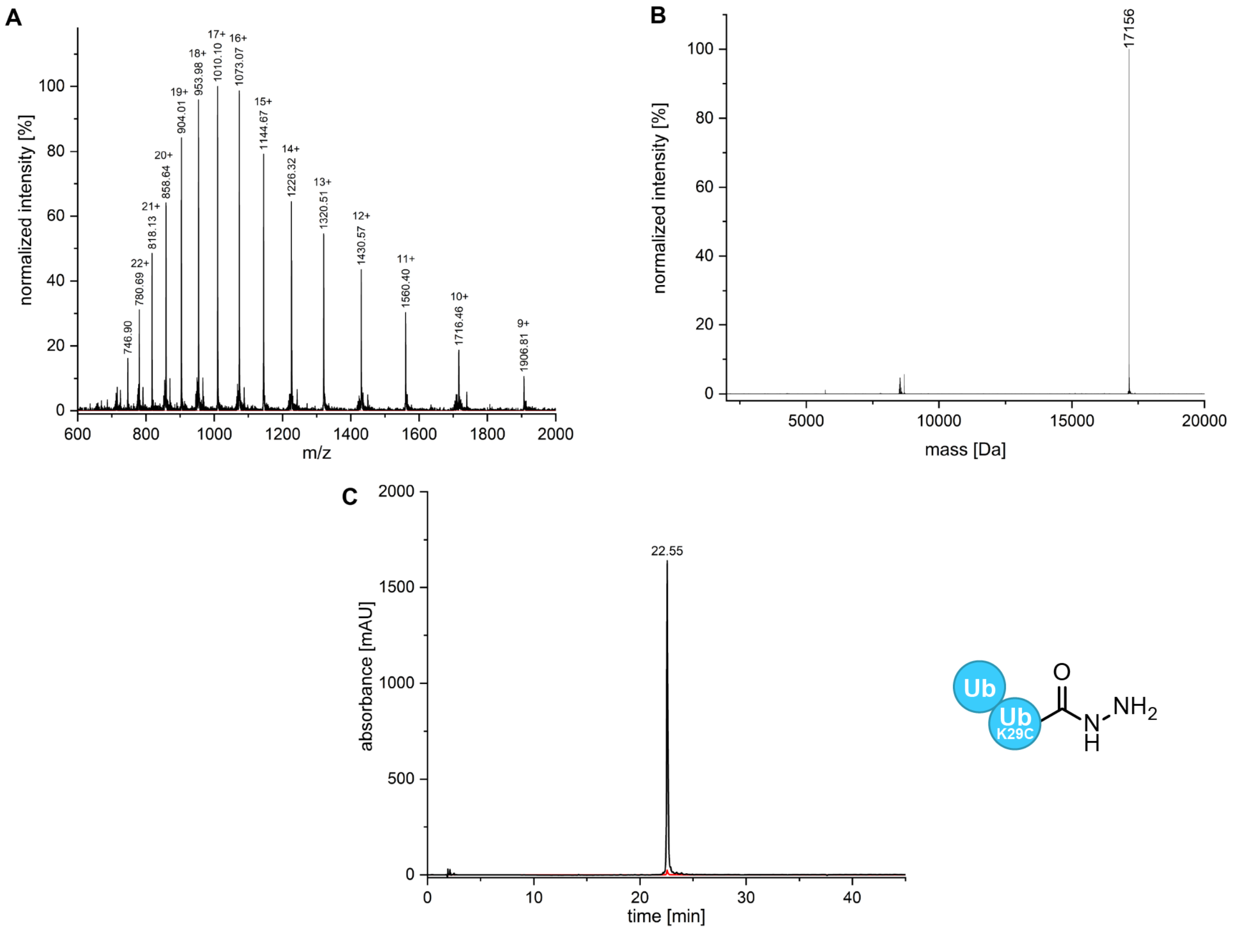

Figure S30. *Characterization of diUb(29)-NHNH$_2$ by ESI (pos.) mass spectrometry (A) with deconvoluted mass spectrum B and analytical RP-HPLC (C; black: 214 nm, red: 280 nm). m_{calc} = 17157.79 Da.*

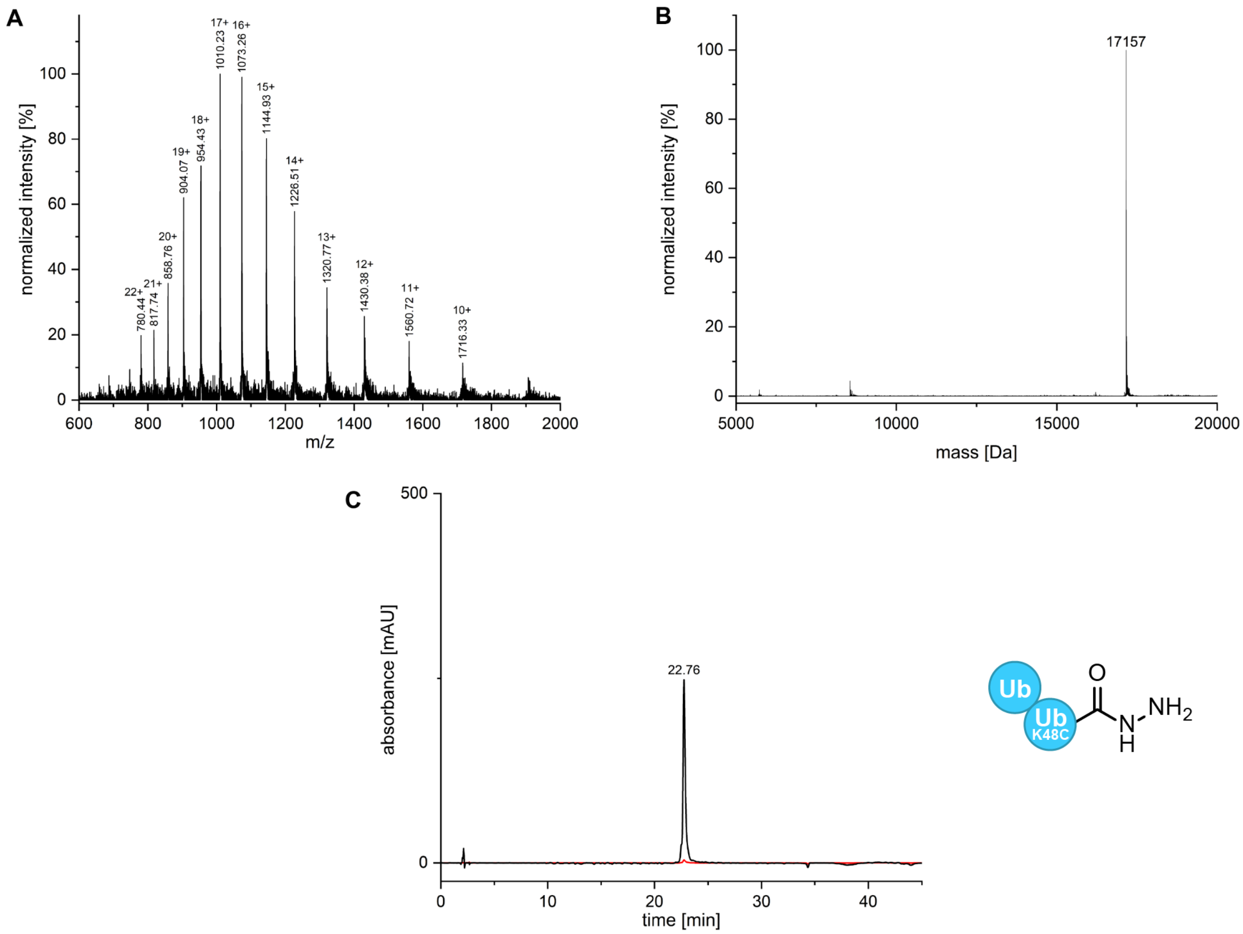

Figure S31. *Characterization of diUb(48)-NHNH$_2$ by ESI (pos.) mass spectrometry (A) with deconvoluted mass spectrum B and analytical RP-HPLC (C; black: 214 nm, red: 280 nm). m_{calc} = 17157.79 Da.*

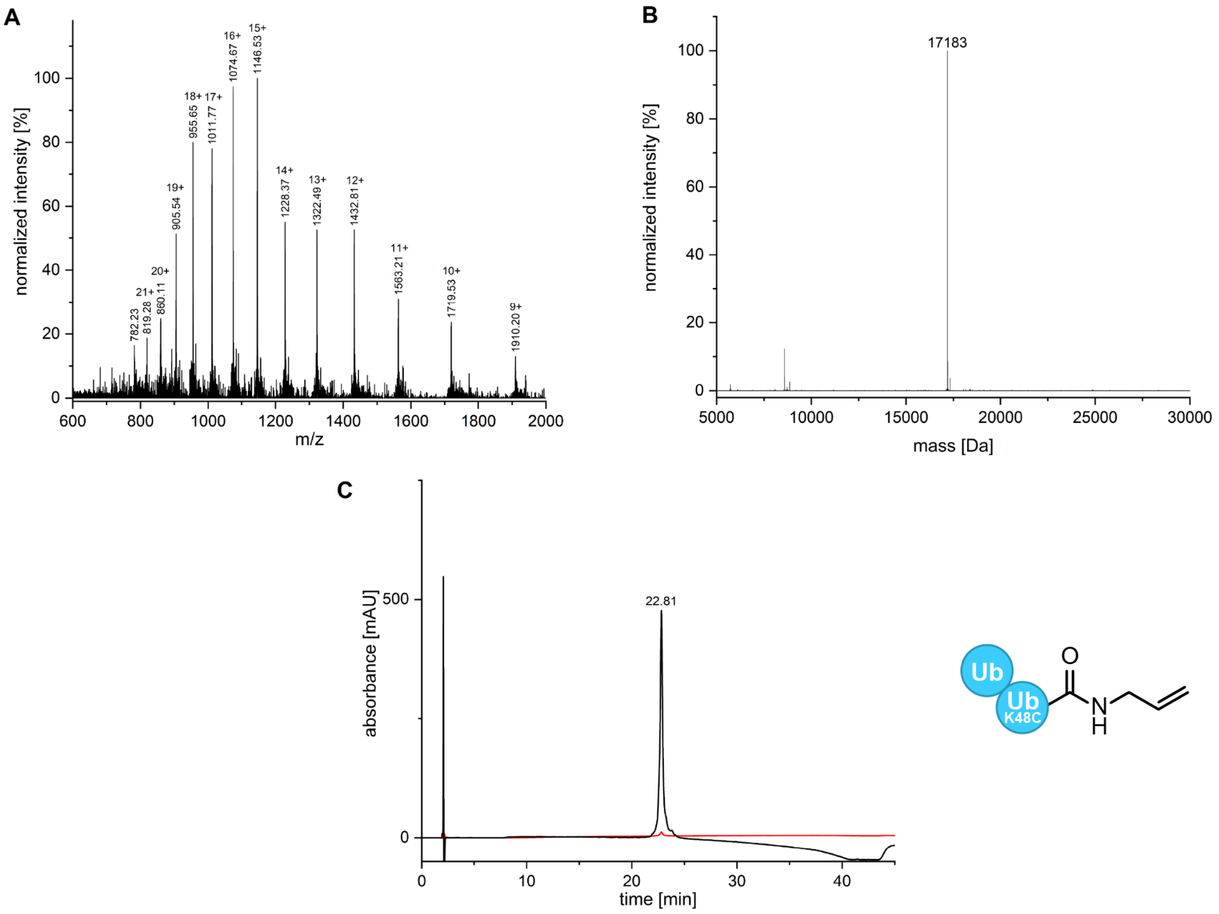

Figure S32. *Characterization of diUb(48)-AA by ESI (pos.) mass spectrometry (A) with deconvoluted mass spectrum B and analytical RP-HPLC (C; black: 214 nm, red: 280 nm).* m_{calc} *= 17182.84 Da.*

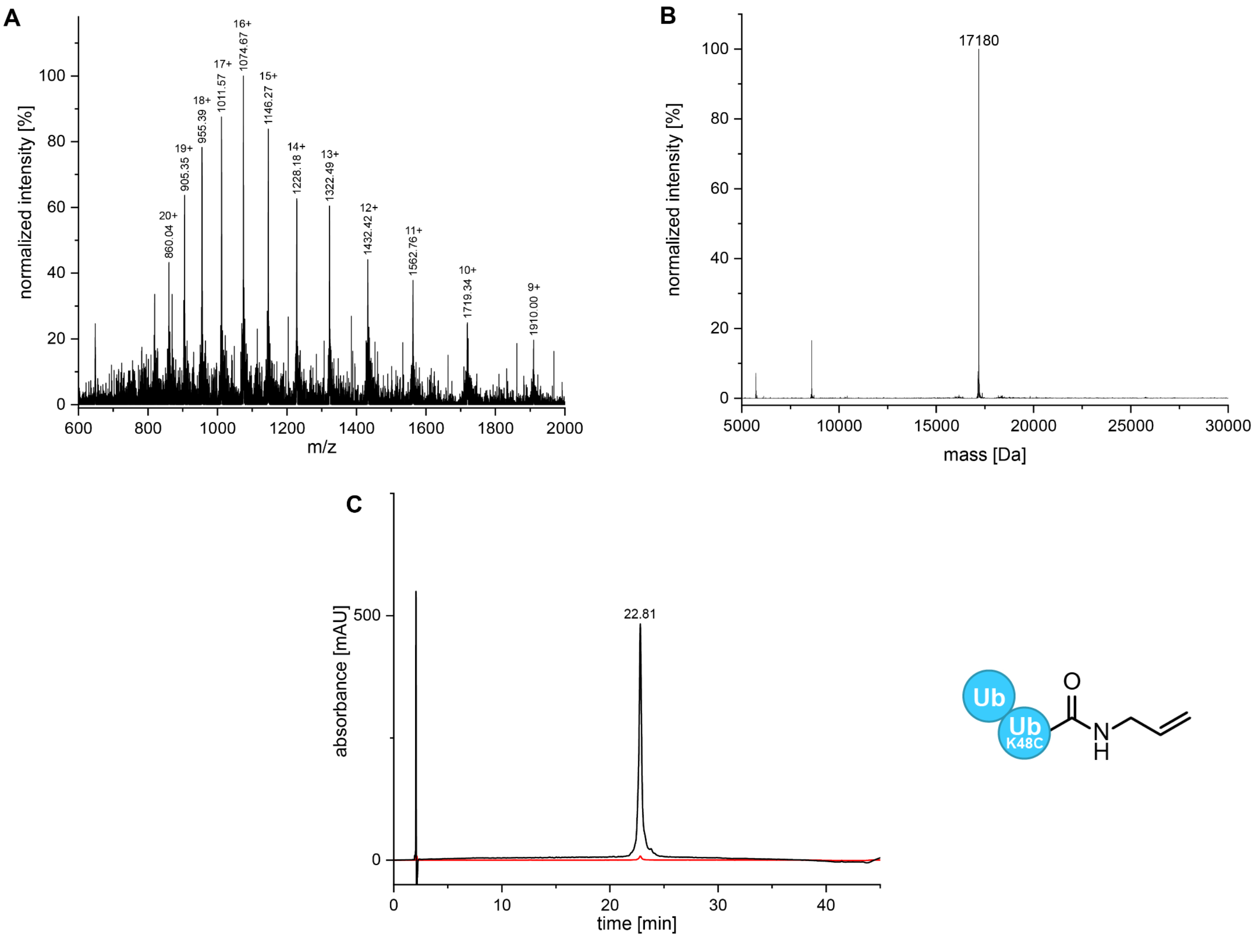

Figure S33. *Characterization of diUb(48)-AA after isolation via ultrafiltration. ESI (pos.) mass spectrum (A) with deconvoluted mass spectrum B and analytical RP-HPLC chromatogram (C; black: 214 nm, red: 280 nm).* m_{calc} *= 17182.84 Da.*

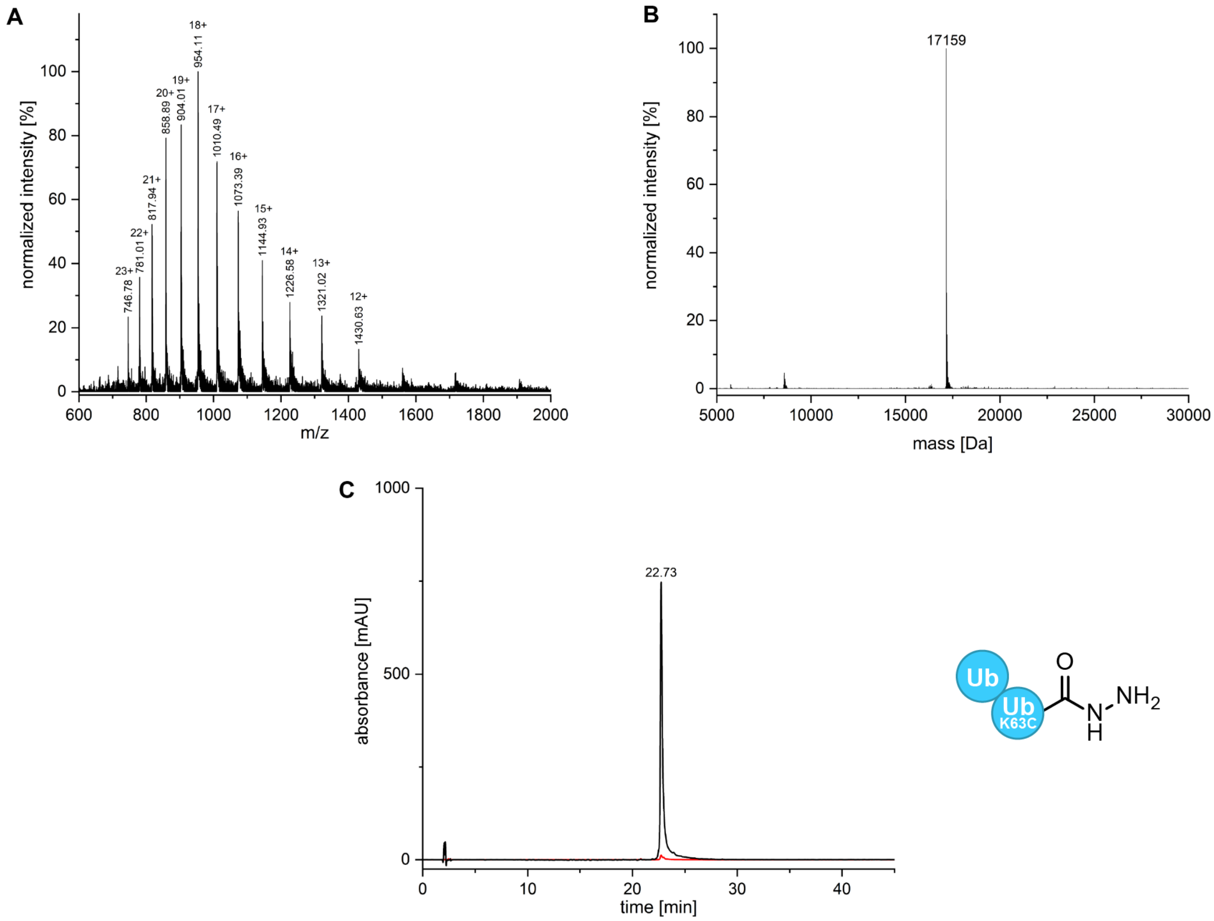

Figure S34. *Characterization of diUb(63)-NHNH$_2$ by ESI (pos.) mass spectrometry (A) with deconvoluted mass spectrum B and analytical RP-HPLC (C; black: 214 nm, red: 280 nm). m_{calc} = 17157.79 Da.*

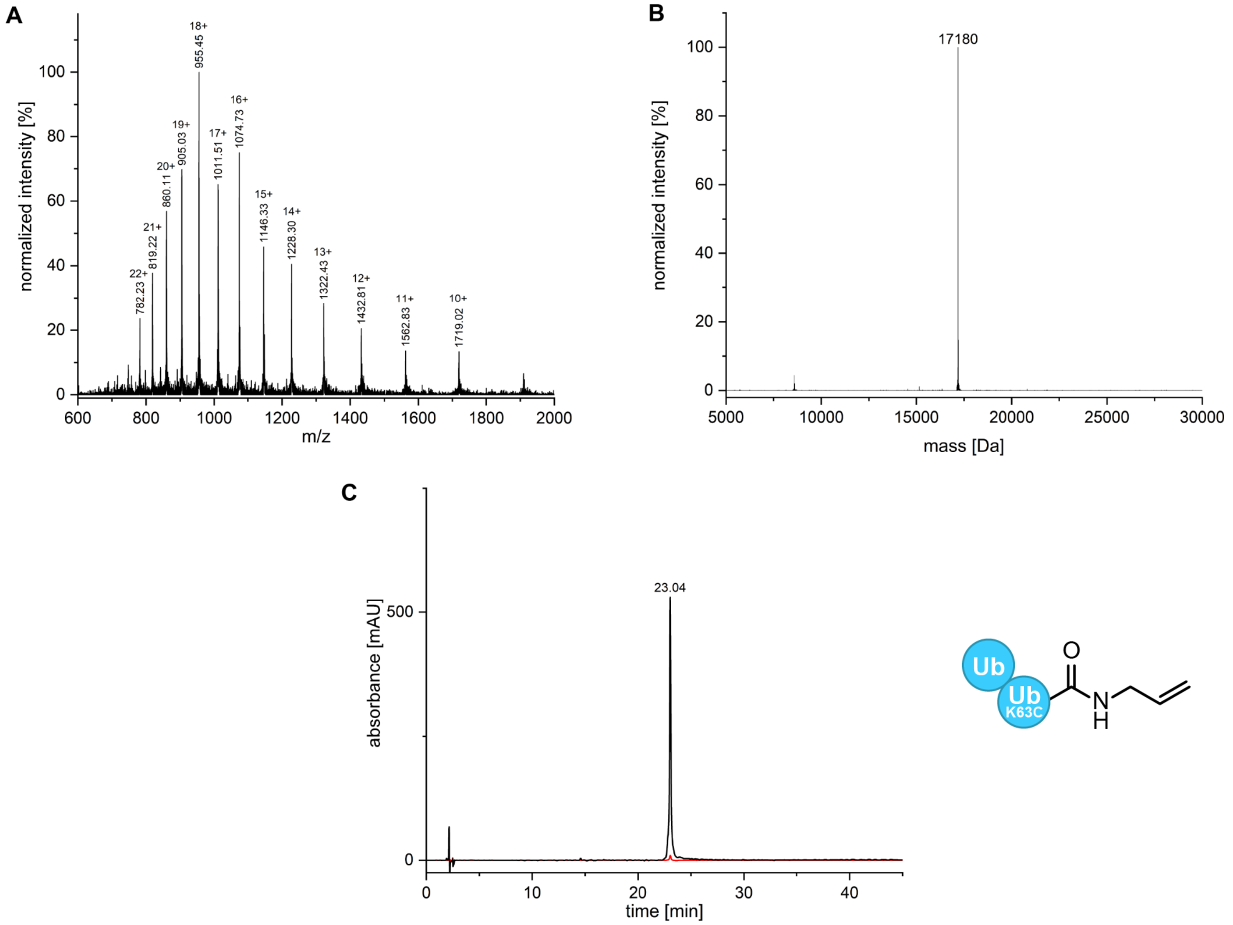

Figure S35. *Characterization of diUb(63)-AA by ESI (pos.) mass spectrometry (A) with deconvoluted mass spectrum B and analytical RP-HPLC (C; black: 214 nm, red: 280 nm).* m_{calc} *= 17182.84 Da.*

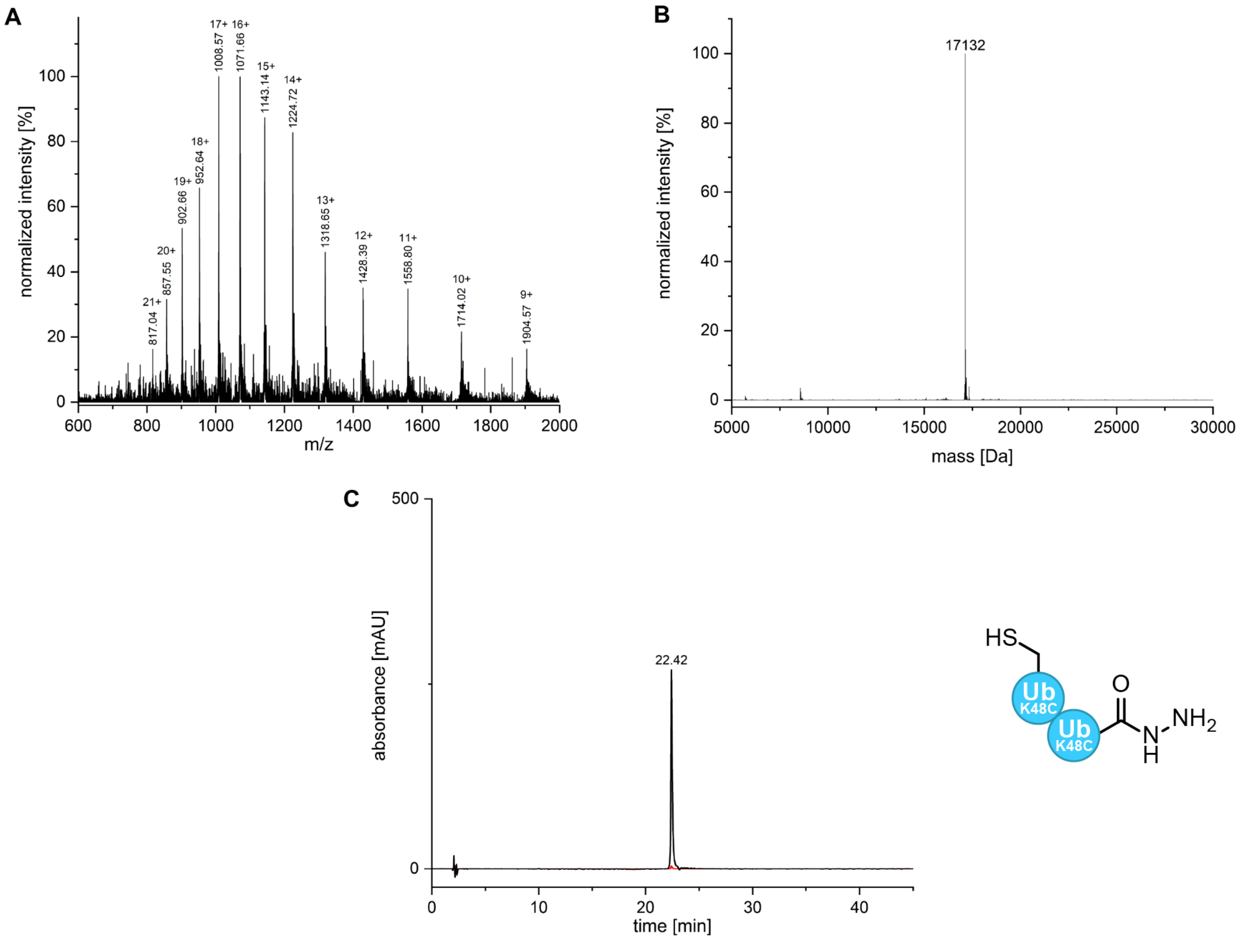

Figure S36. *Characterization of diUb(48-48)-NHNH$_2$ by ESI (pos.) mass spectrometry (A) with deconvoluted mass spectrum B and analytical RP-HPLC (C; black: 214 nm, red: 280 nm). m_{calc} = 17132.79 Da.*

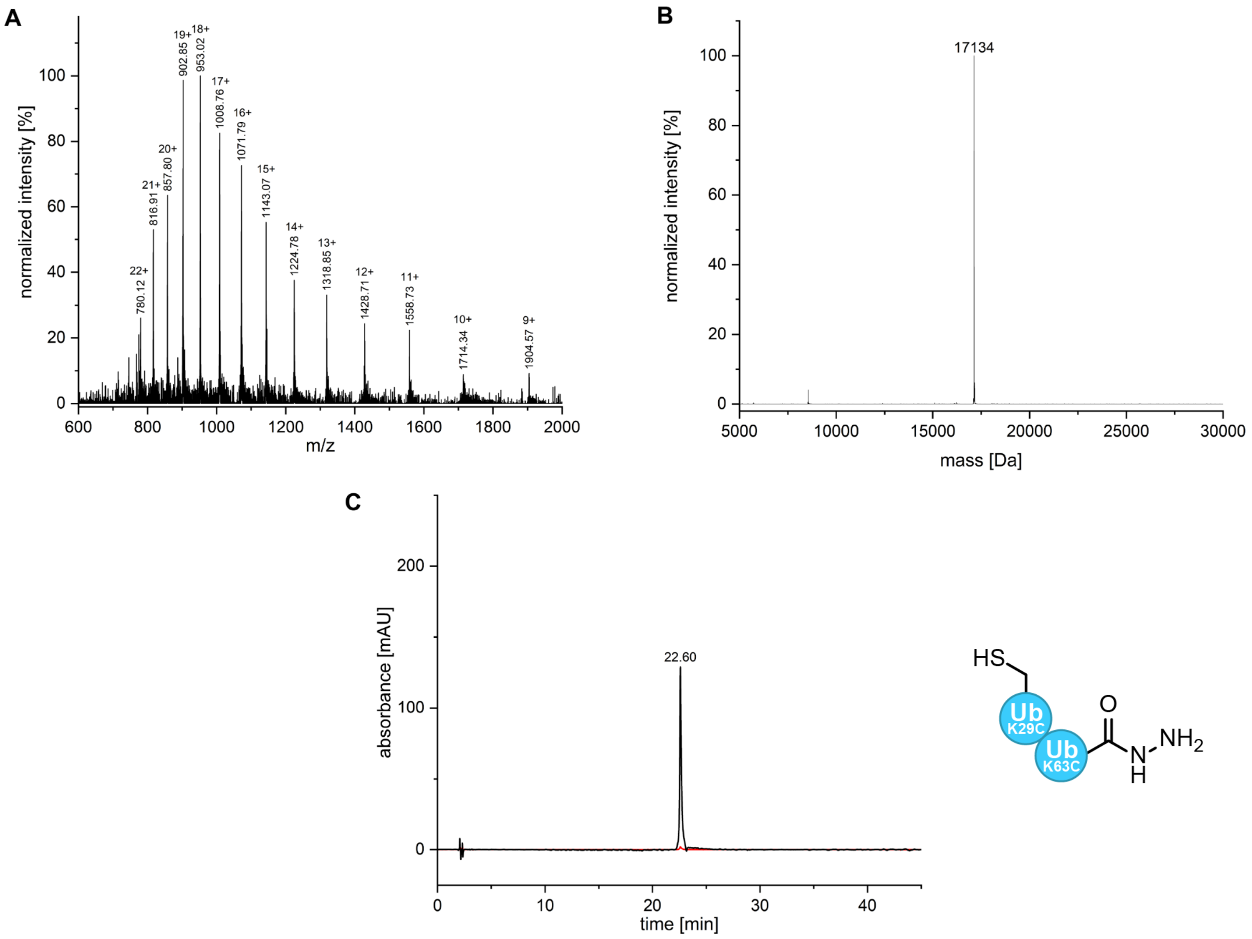

Figure S37. *Characterization of diUb(29-63)-NHNH$_2$ by ESI (pos.) mass spectrometry (A) with deconvoluted mass spectrum B and analytical RP-HPLC (C; black: 214 nm, red: 280 nm). m$_{calc}$ = 17132.79 Da.*

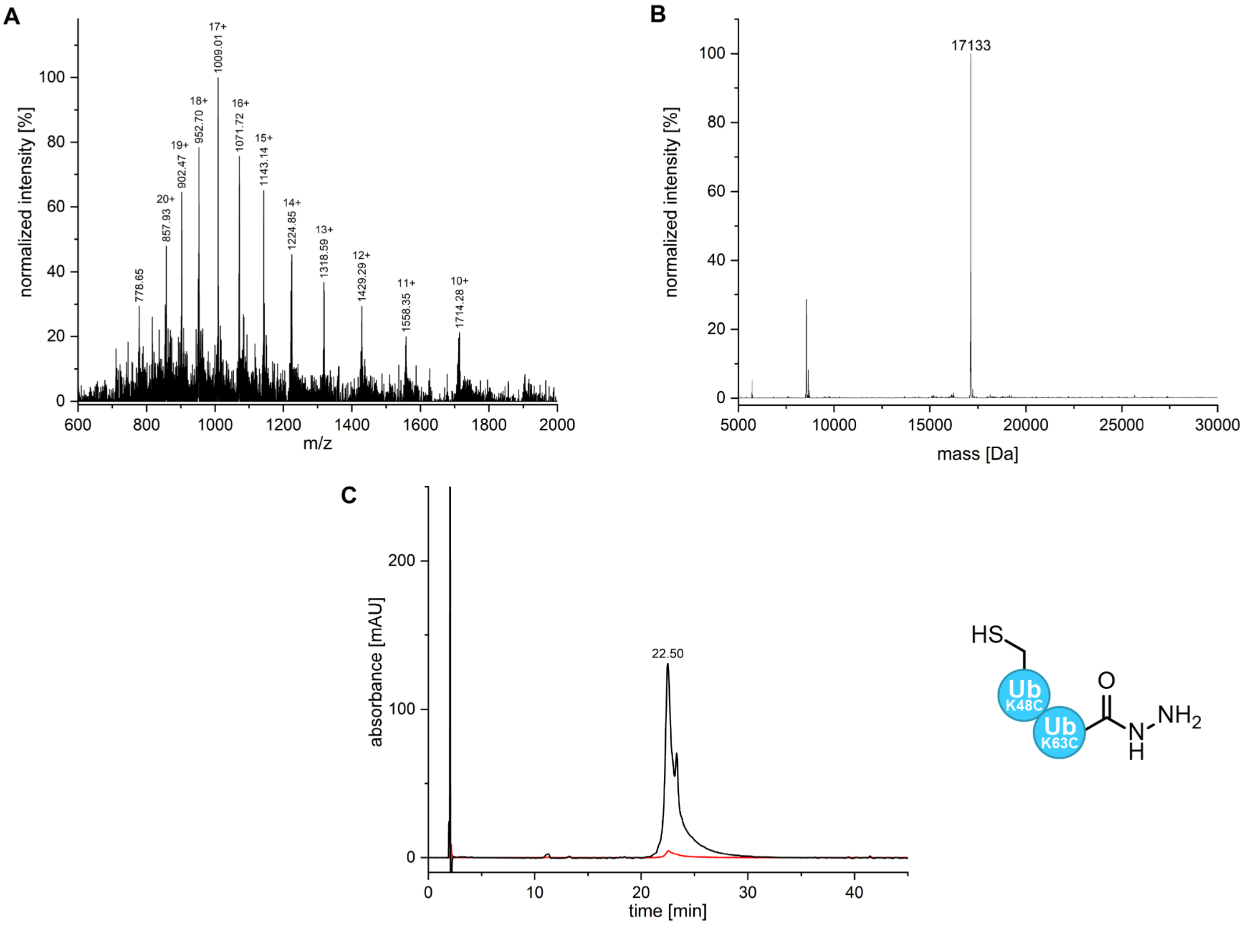

Figure S38. *Characterization of diUb(48-63)-NHNH$_2$ by ESI (pos.) mass spectrometry (A) with deconvoluted mass spectrum B and analytical RP-HPLC (C; black: 214 nm, red: 280 nm). m_{calc} = 17132.79 Da.*

Characterization of tri-ubiquitin motifs

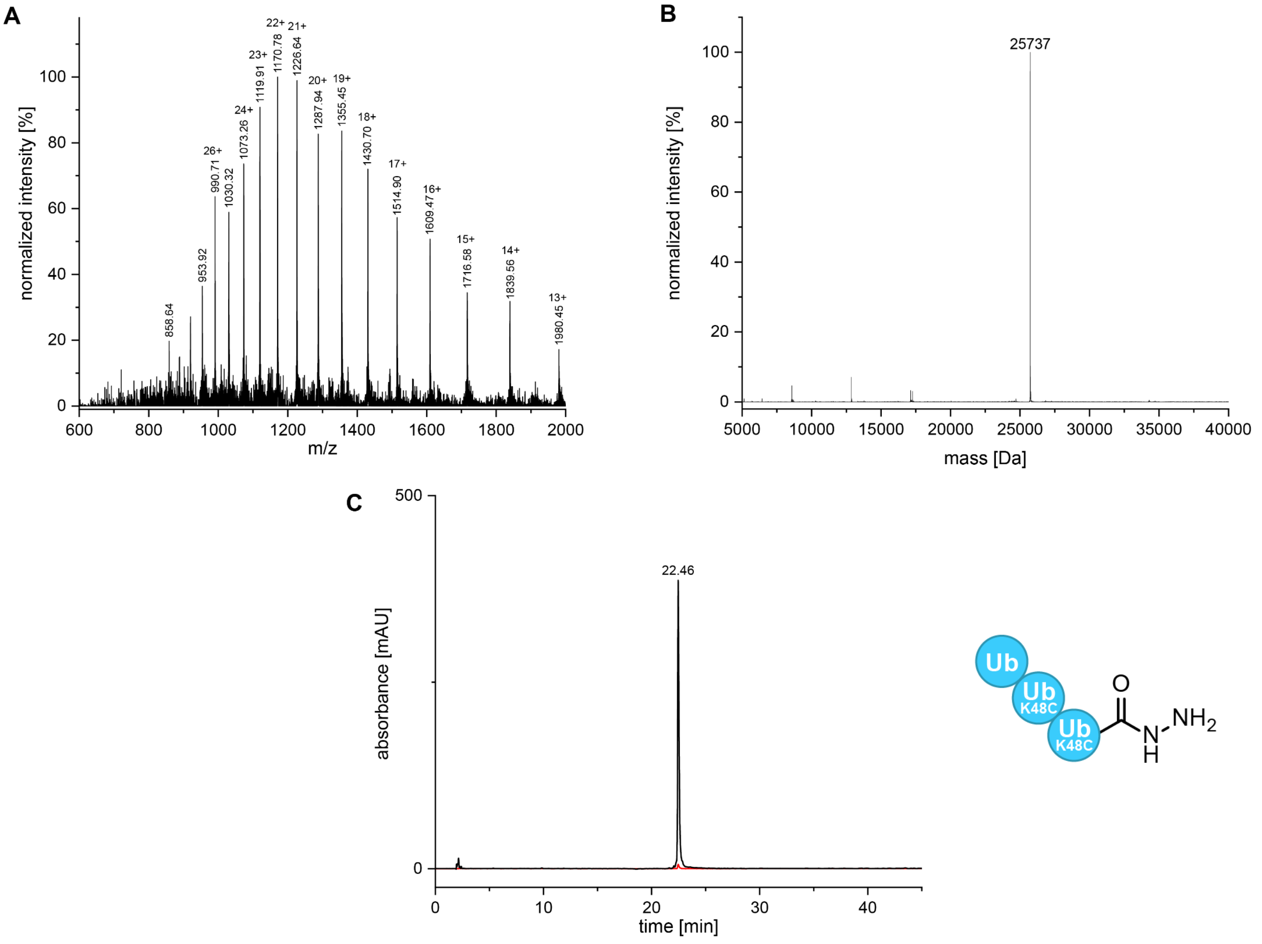

Figure S39. *Characterization of triUb(48/48)-NHNH$_2$ by ESI (pos.) mass spectrometry (A) with deconvoluted mass spectrum B and analytical RP-HPLC (C; black: 214 nm, red: 280 nm). m_{calc} = 25736.71 Da.*

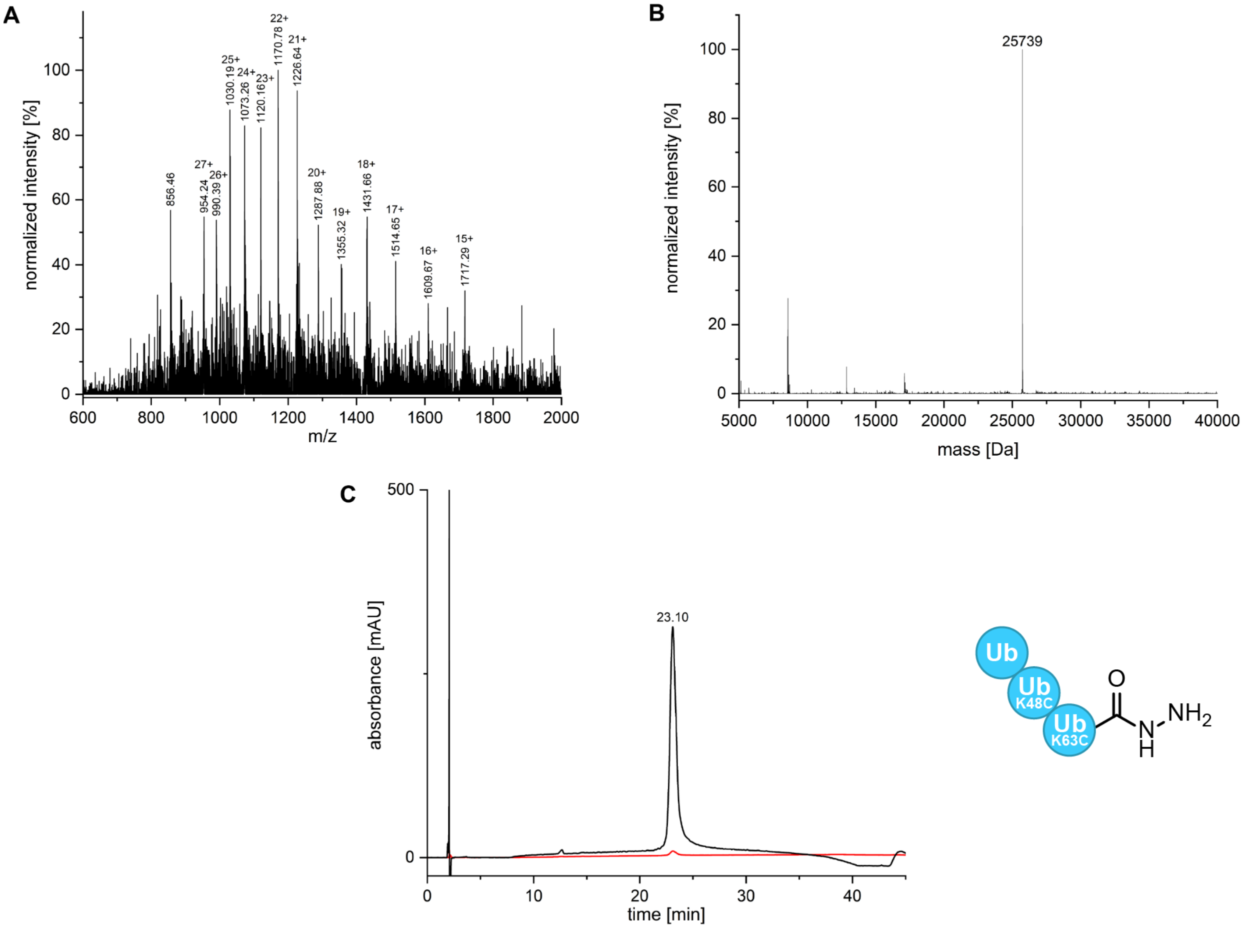

Figure S40. *Characterization of triUb(48/63)-NHNH$_2$ by ESI (pos.) mass spectrometry (A) with deconvoluted mass spectrum B and analytical RP-HPLC (C; black: 214 nm, red: 280 nm). m$_{calc}$ = 25736.71 Da.*

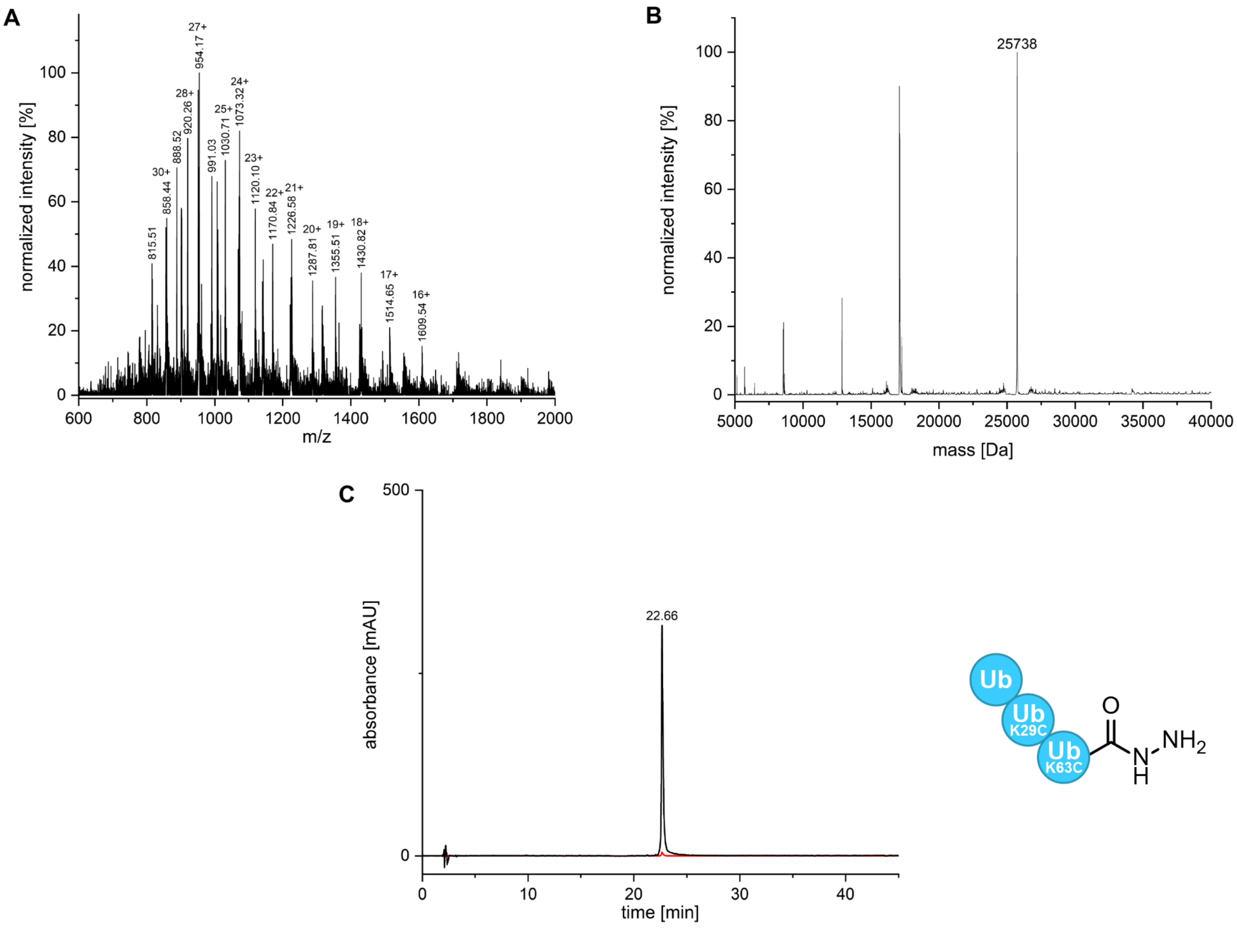

Figure S41. *Characterization of triUb(29/63)-$NHNH_2$ by ESI (pos.) mass spectrometry (A) with deconvoluted mass spectrum B and analytical RP-HPLC (C; black: 214 nm, red: 280 nm). m_{calc} = 25736.71 Da.*

Charactzerization of tetra-ubiquitin

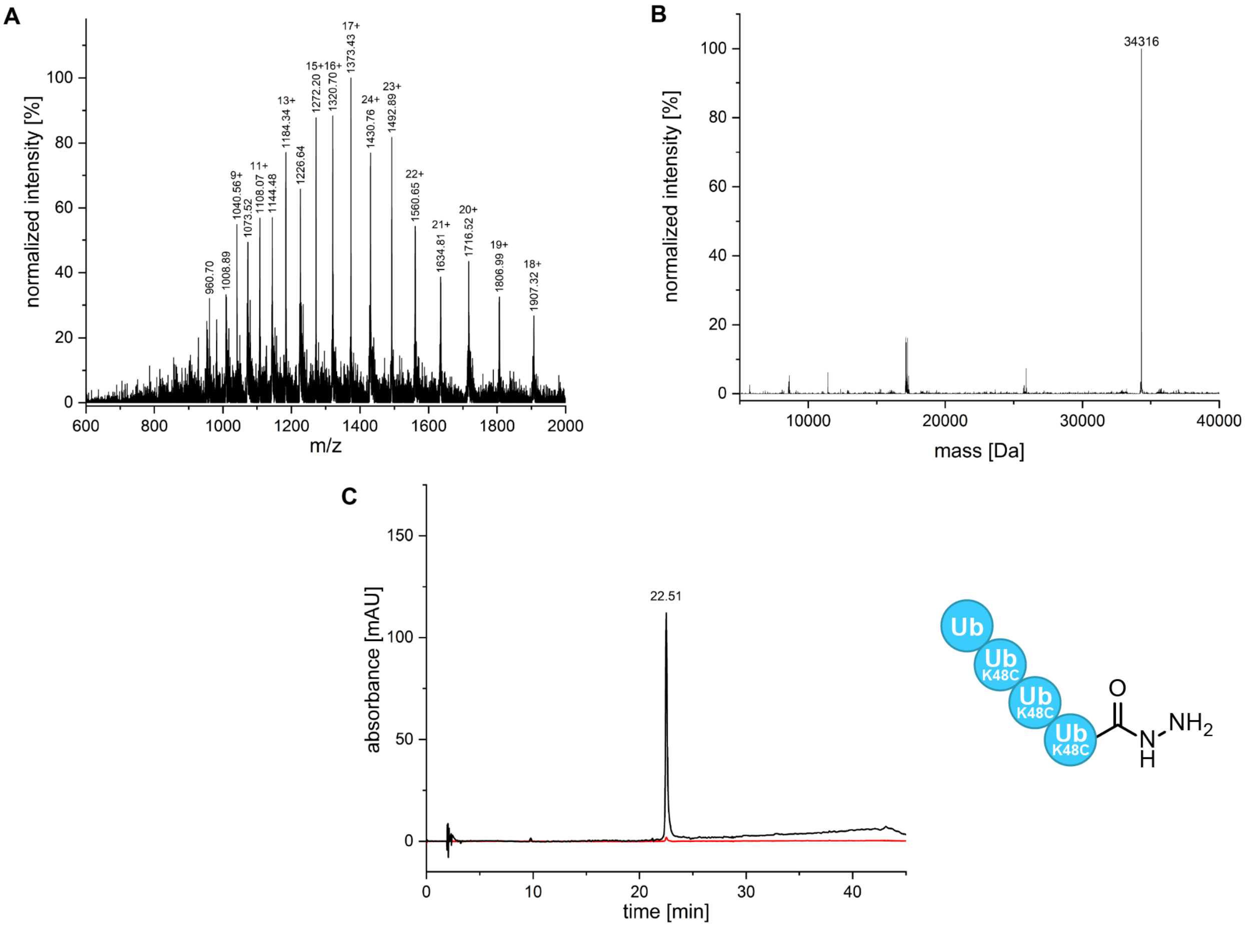

Figure S42. *Characterization of tetraUb(48/48/48)-NHNH$_2$ by ESI (pos.) mass spectrometry (A) with deconvoluted mass spectrum B and analytical RP-HPLC (C; black: 214 nm, red: 280 nm). m_{calc} = 34315.58 Da.*

Ubiquitylated tau peptides

Peptide sequences:

Tau4(292-326): GSKDNIKHVPGGGSVQIVYCPVDLSKVTSKCGSLG

Tau4(M+266-274+306-321): MLKHQPGGGKVQIVYCPVDLSKVTSK-NHNH$_2$

Tau4(322-391): CGSLGNIHHKPGGGQVEVKSEKLDFKDRVQSKIGSLDNITHV PGGGNKKIETHKLTFRENAKAKTDHGAE

Tau4(M+266-274+306-391): MLKHQPGGGKVQIVYCPVDLSKVTSKCGSLGNIHHKPGGG QVEVKSEKLDFKDRVQSKIGSLDNITHV PGGGNKKIETHKLTFRENAKAKTDHGAE

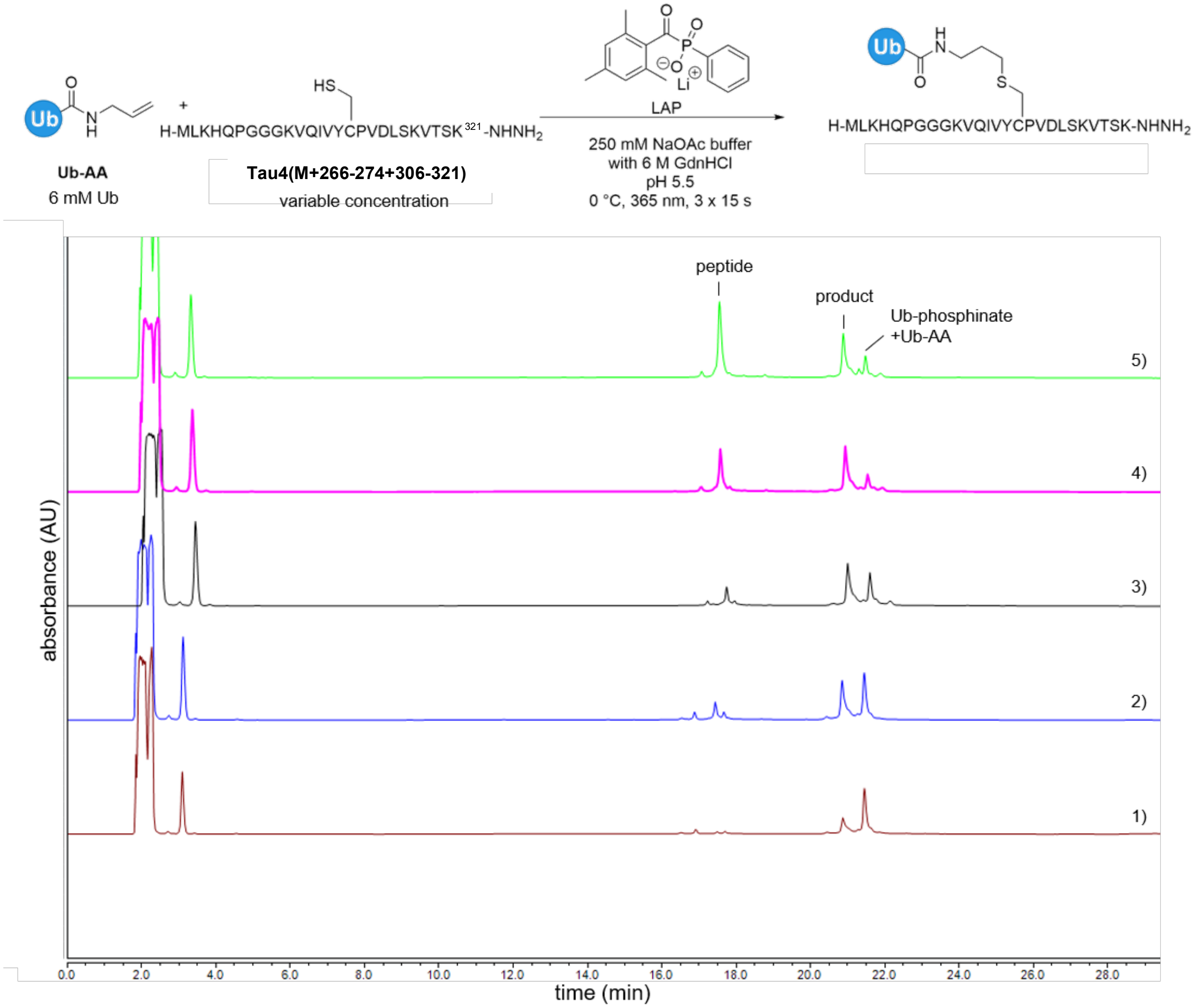

Figure S43. Ubiquitylation of tau-derived peptide at various concentrations. *Analytical RP-HPLC (C4, 214 nm) of reaction mixtures after reaction of 6 mM Ub-AA with 7.5 mM (1), 10.5 mM (2), 15 mM (3), 22 mM (4) and 30 mM (5).*

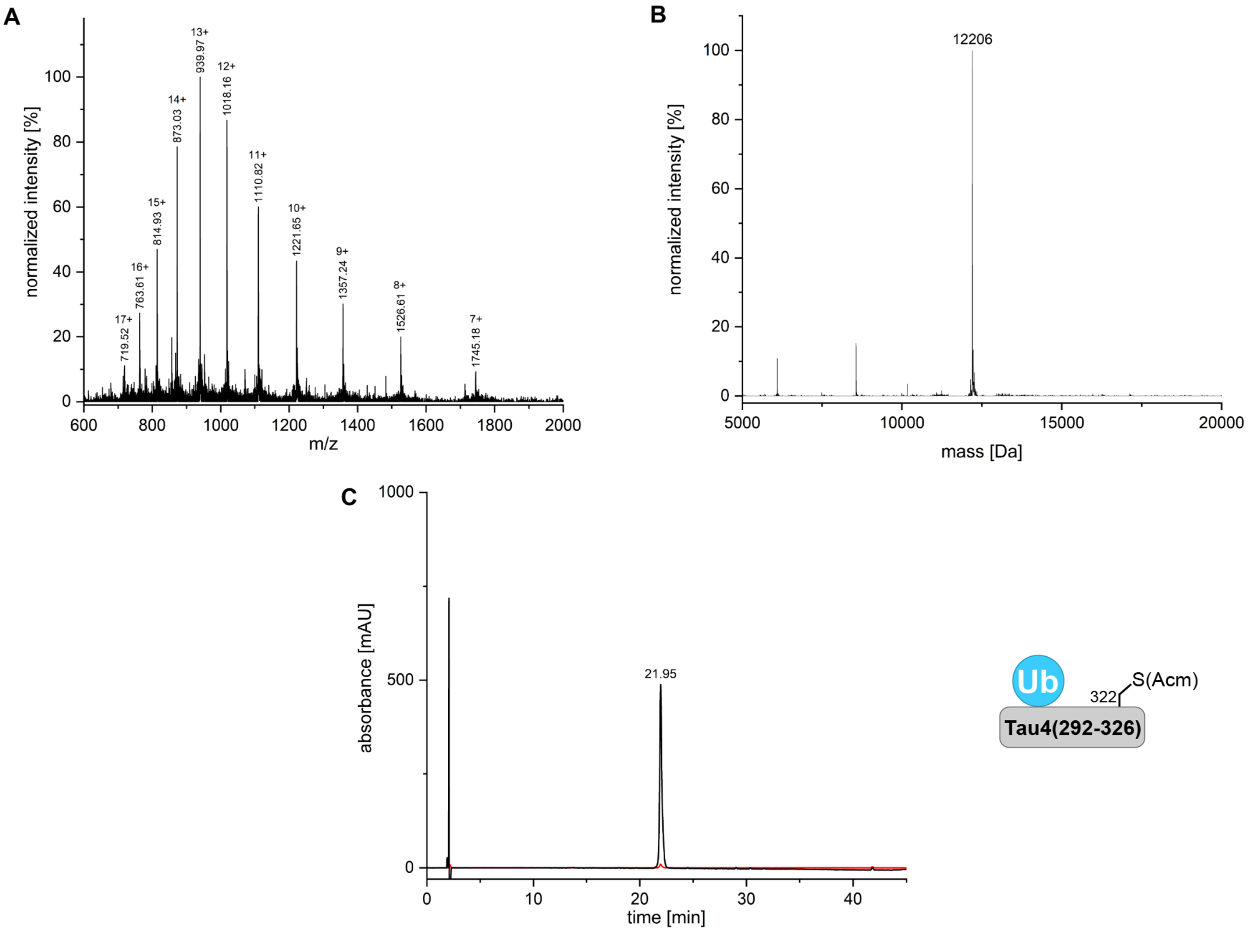

Figure S44. *Characterization of Ub-Tau4(292-326, C322Acm) by ESI (pos.) mass spectrometry (A) with deconvoluted mass spectrum B and analytical RP-HPLC (C; black: 214 nm, red: 280 nm).* m_{calc} *= 12206 Da.*

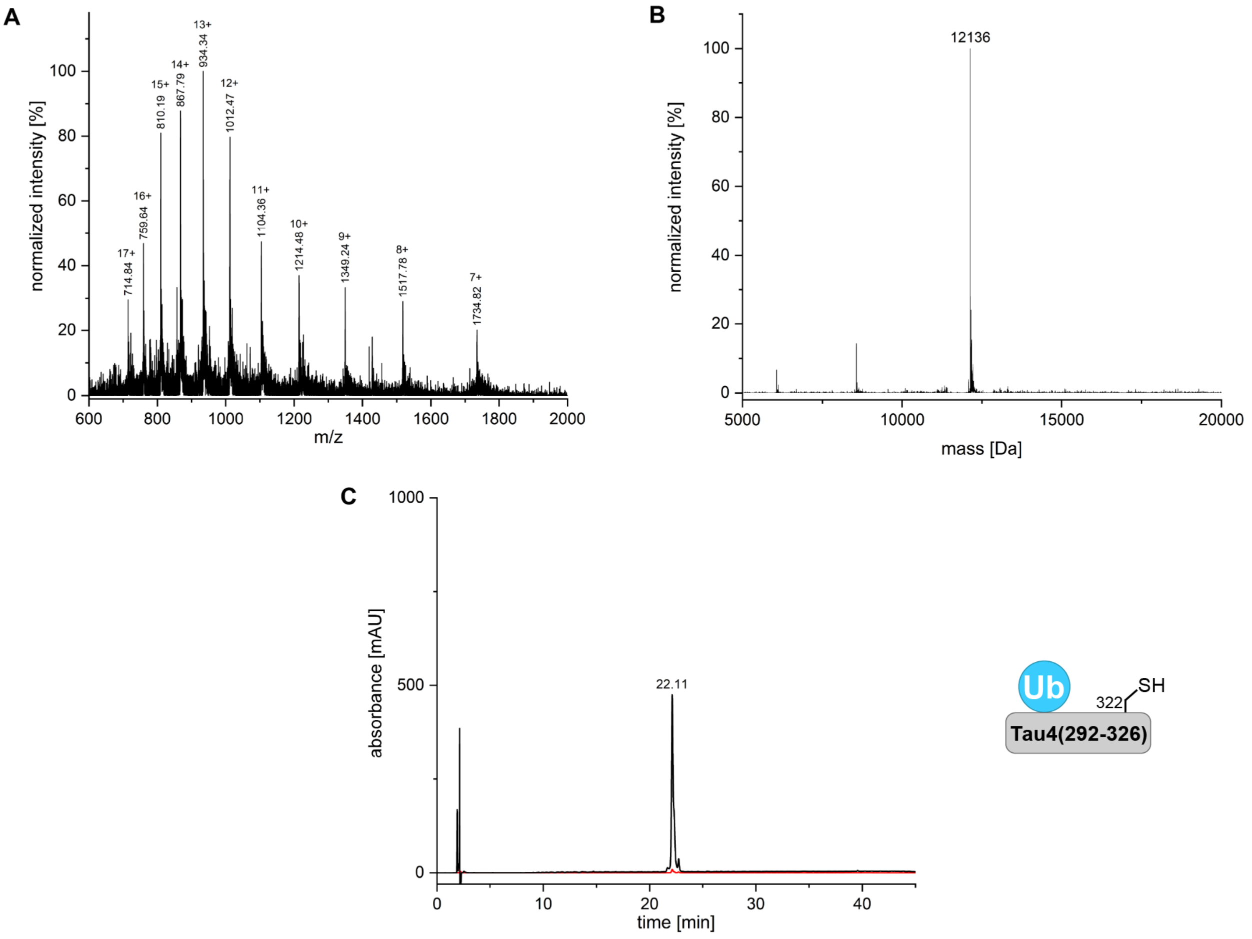

Figure S45. *Characterization of Ub-Tau4(292-326) by ESI (pos.) mass spectrometry (A) with deconvoluted mass spectrum B and analytical RP-HPLC (C; black: 214 nm, red: 280 nm).* m_{calc} *= 12138 Da.*

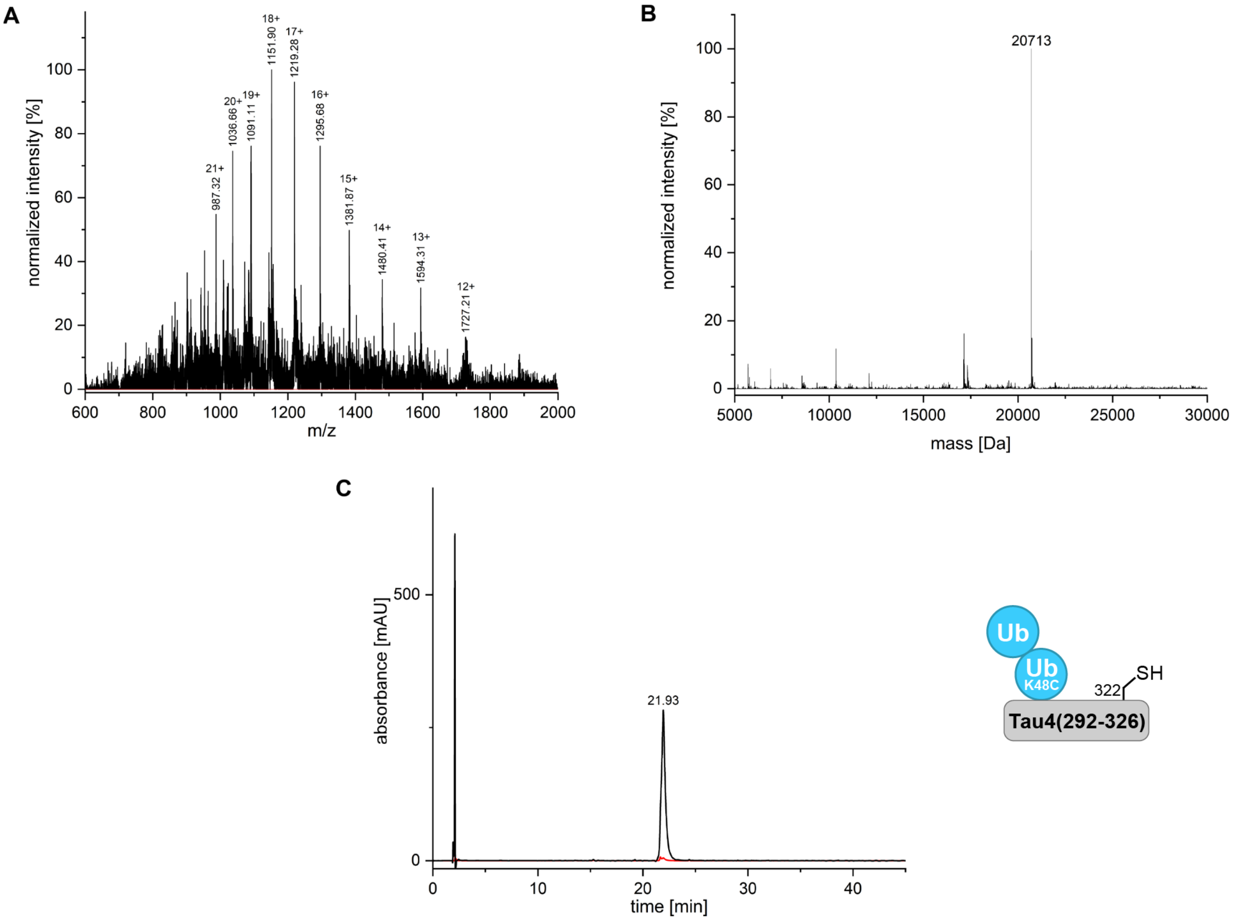

Figure S46. *Characterization of diUb(48)-Tau4(292-326) by ESI (pos.) mass spectrometry (A) with deconvoluted mass spectrum B and analytical RP-HPLC (C; black: 214 nm, red: 280 nm).* m_{calc} *= 20713Da.*

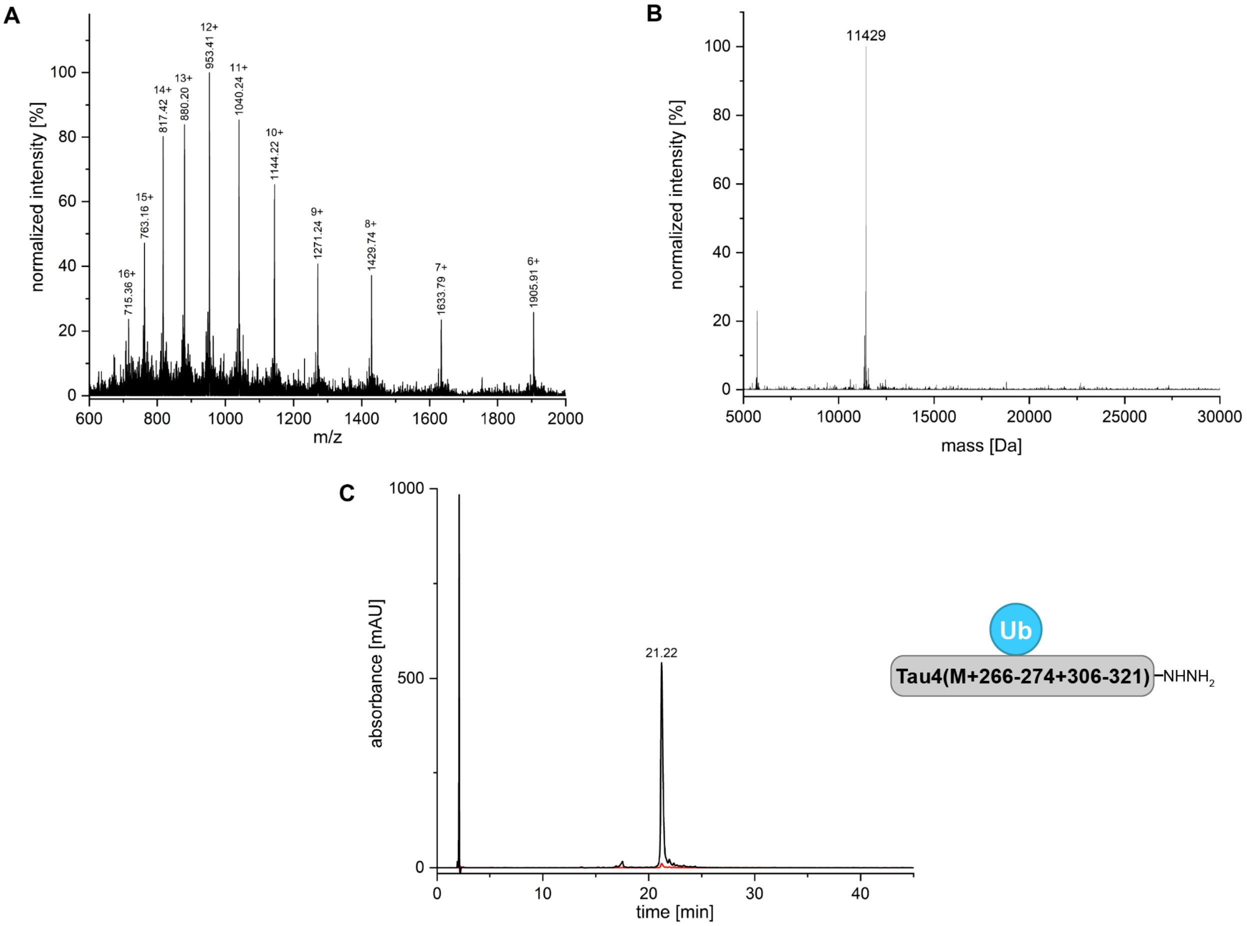

Figure S47. *Characterization of Ub-Tau4(M+266-274+306-321)-$NHNH_2$ by ESI (pos.) mass spectrometry (A) with deconvoluted mass spectrum B and analytical RP-HPLC (C; black: 214 nm, red: 280 nm).* m_{calc} *= 11431 Da.*

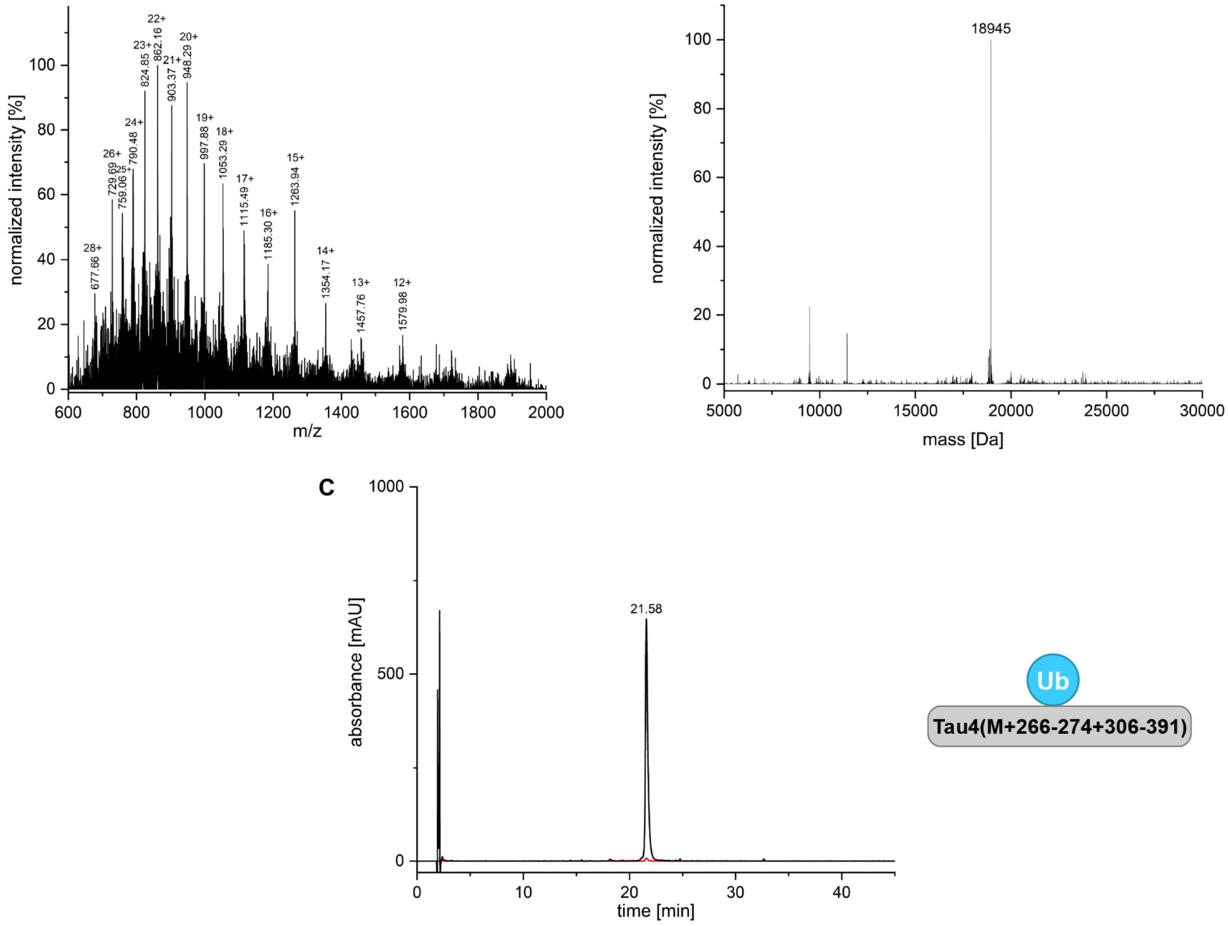

Figure S48. *Characterization of Ub-Tau4(M+266-274+306-391) by ESI (pos.) mass spectrometry (A) with deconvoluted mass spectrum B and analytical RP-HPLC (C; black: 214 nm, red: 280 nm).* m_{calc} *= 18946 Da.*

DUB-Assay:

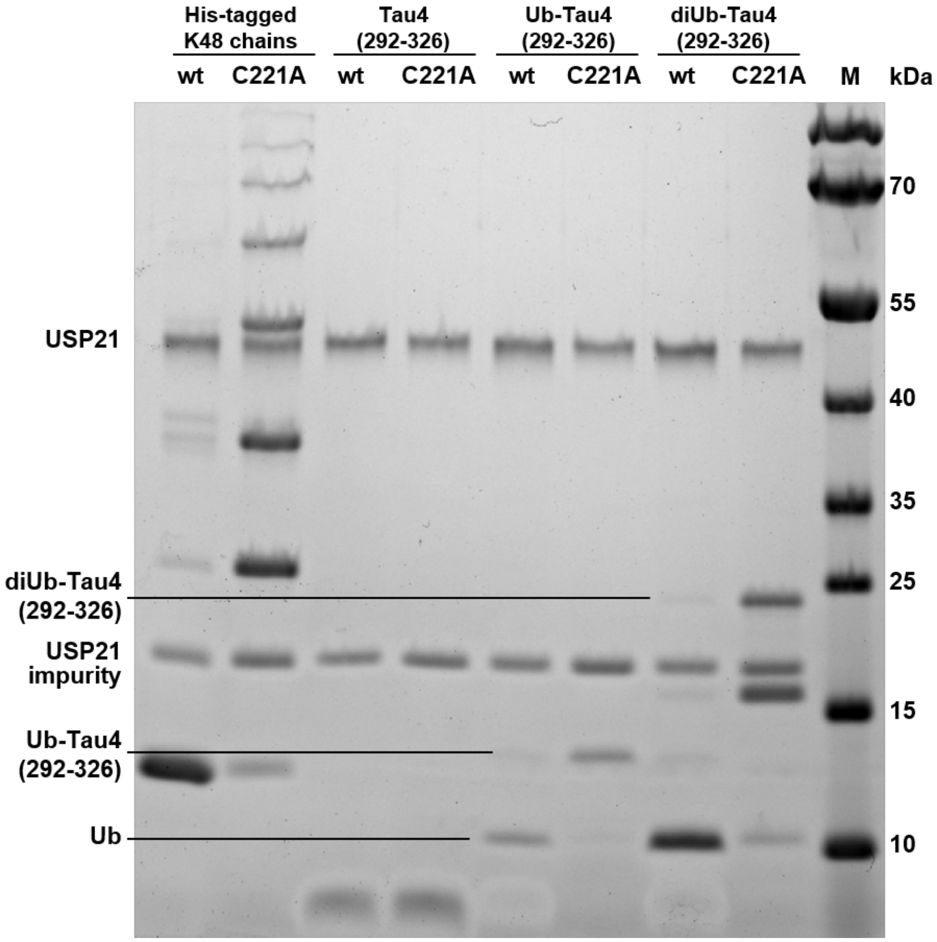

Figure S49. *Processing of ubiquitylated Tau4(292-326) by USP21. SDS-PAGE of reaction mixtures incubated 60 min with functional (wt) USP21 and non-functional (C221A) USP21 mutant. Lane 1+2: control with His-tagged K48 chain mixture; lane 3+4: unmodified Tau4(292-326); lane 5+6: Ub-Tau4(292-326); lane 7+8: diUb(48)-Tau4(292-326).*

Synthesis and Characterization of Ub-Tau4(2-441)

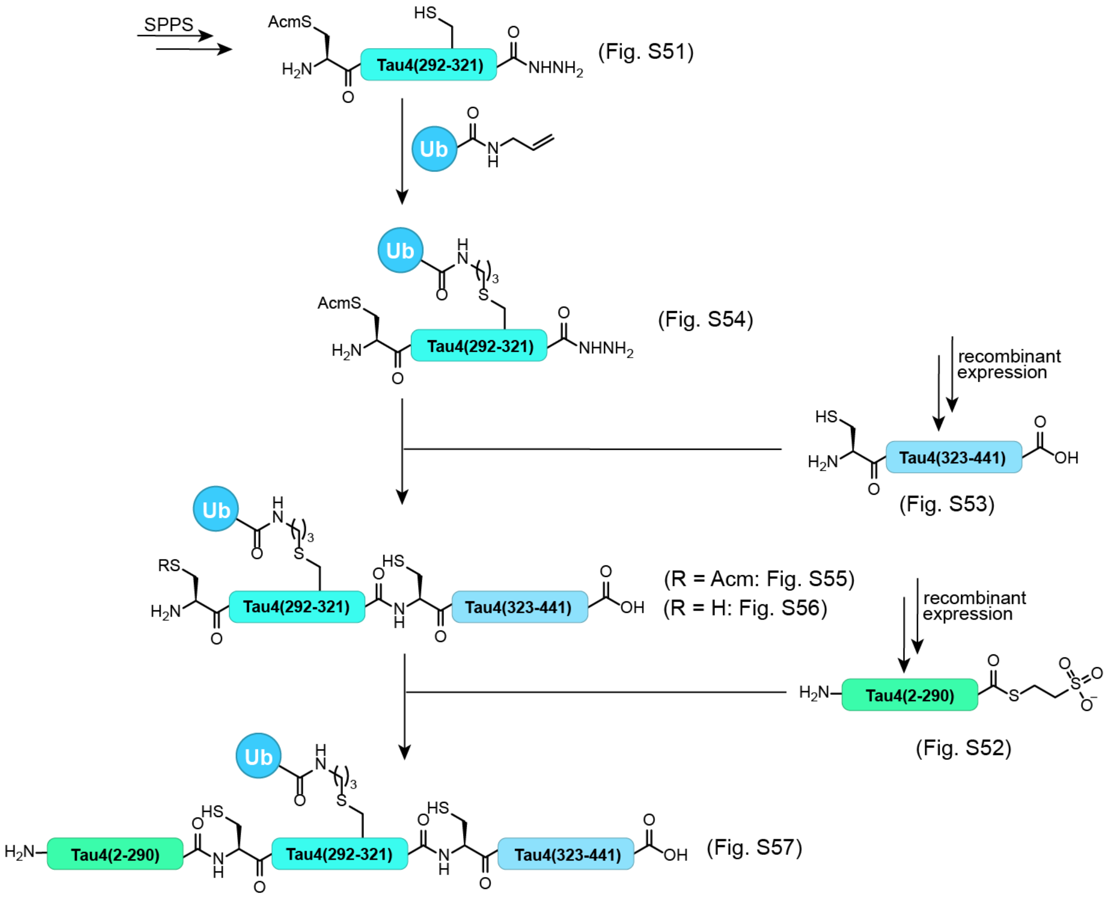

Figure S50. *Overview of the synthesis of site-specifically ubiquitylated Tau4.*

Generation of ubiquitylated Ub-Tau4(291-321,K311C,C291Acm)-$NHNH_2$

Tau4(291-321,K311C,C291Acm)-$NHNH_2$ was synthesized using standard Fmoc-SPPS. (Figure S51)

Tau4(291-321,K311C,C291Acm)-$NHNH_2$ (15.06 mg, 3.56 µmol) was dissolved in 230 µL of 250 mM NaOAc buffer with 6 M Gdn-HCl (pH 5.5) and Ub(wt)-AA (31.15 mg, 2.85 µmol) was dissolved in 214.5 µL of the same buffer and the solutions were mixed in a Schlenk tube. 30.1 µL of a 75 mM LAP stock solution were added to reach final concentrations of 6 mM Ub(wt)-AA, 7.5 mM Tau4(291-321,K311C,C291Acm)$NHNH_2$ and 4.75 mM LAP in 250 mM NaOAc buffer with 6 M Gdn-HCl. The pH of the reaction mixture was pH 4.4. The Schlenk tube was equipped with a magnetic stirrer, placed on ice and the reaction mixture was irradiated from above with 365 nm light (83 mW/cm^2) for 1 min total (30 sec on + 30 sec off, 2×). The reaction mixture was diluted with 2 mL 6 M Gdn-HCl, freeze-dried and subjected to semipreparative RP-HPLC (C4, 60 °C,

3 mL/min; 5-25%B in 10 min, 25-40%B in 40 min). Product containing fractions were pooled and lyophilized (Figure S54).

Ligation of Ub-Tau4(291-321,C291Acm)-$NHNH_2$ with the C-terminal Tau4(322-441):

Tau4(322-441) was generated by recombinant expression as reported previously.[44] (Figure S53) Buffers were degassed via argon or nitrogen purging for at least 5 min before usage. Ub-Tau4(291-321,C291Acm)-$NHNH_2$ was dissolved in 6 M Gdn-HCl, 200 mM Sodium Phosphate Buffer (pH 3.0) and cooled to -15 °C for 5 min (final concentration: 6 mM). A stock solution of $NaNO_2$ (0.5 M) in 6 M Gdn-HCl, 200 mM Sodium Phosphate Buffer, pH 3.0) was prepared and cooled to -15 °C for 5 min. An aliquot of the $NaNO_2$ stock was added to the reaction mixture to a reach a final concentration of 50 mM $NaNO_2$ and the mixture was stirred at -15 °C for 20 min.
An MPAA stock solution (0.6 M) in 6 M Gdn-HCl, 200 mM Sodium Phosphate Buffer, pH 9.0 was prepared and kept at room temperature. The reaction mixture was diluted with MPAA stock 1:1 (v/v) and the pH was adjusted carefully to 6.8 and kept at room temperature for 5 min at a peptide concentration of 3 mM before proceeding. The reaction mixture was transferred to a new 2 mL Eppendorf reaction vessel containing lyophilized Tau4(322-441) (1.5 mM). The ligation solution was vortexed briefly and the pH was readjusted carefully to 6.8. The ligation reaction was performed at 30 °C for 3h–4h under constant agitation and argon atmosphere. Prior to reaction monitoring and purification, the reaction was quenched using a quenching buffer (10% (v/v) β-mercaptoethanol, 0.1 M TCEP, 6 M Gdn-HCl, 0.2 M Sodium Phosphate Buffer (pH 3)). Quenching was followed by centrifugation at 14000 rpm for 5 min and subsequent analytical characterization or RP-HPLC purification. Samples for analytical gel electrophoresis additionally were precipitated using ethanol. The product was isolated using semipreparative RP-HPLC (Kromasil C18, 60 °C; 10 mL/min; linear gradient 5%–60%B in 50 min). Product containing fractions were pooled and lyophilized (Figure S55).

Acm removal to generate Ub-Tau4(291-441):

Cystein deprotection was performed as described by Wang et al.[52] Ub-Tau4(291-441,C291Acm) was dissolved in previously degassed 85% (v/v) acetic acid (1 mg/100 µL). AgOAc (1 mg/100 µL) was added and the reaction mixture was stirred for 60 min at 30 °C under argon. The reaction was monitored via LC-MS. Therefore, reaction aliquots and the reaction mixture were quenched by dilution (1:1 v/v) with 1 M DTT, 6 M Gdn-HCl, 0.2 M Sodium Phosphate Buffer (pH 3) and stirred for 15 min at 30 °C under argon. Quenching was followed by centrifugation at 14000 rpm for 5 min. The supernatant was then analyzed by LC-MS and purification occurred by semipreparative RP-HPLC (Kromasil C18, 60 °C, 10 mL/min, linear gradient 5%–95%B in 30 min. Product containing fractions were pooled and lyophilized (Figure S56)

Generation of Ub-Tau4(2-441)

Tau4(2-290)-MesNa thioester was generated by recombinant expression as reported previously. [44] (Figure S52)

Tau4(2-290)-MesNa thioester (4 mM) and Ub-Tau4(291-441) (0.75 mM) were dissolved in degassed ligation buffer (200 mM MPAA, 100 mM TCEP, 6 M Gdn-HCl, 200 mM Sodium Phosphate Buffer, pH 7.1). The solutions were mixed, vortexed briefly and the pH was readjusted carefully to 7.1. The ligation reaction was performed at 37 °C for 3h–4h under constant agitation and inert atmosphere. Prior to reaction monitoring and purification, the reaction was quenched using a quenching buffer (10% (v/v) β-mercaptoethanol, 0.1 M TCEP, 6 M Gdn-HCl, 0.2 M Sodium Phosphate Buffer (pH 3)). Quenching was followed by centrifugation at 14000 rpm for 5 min and subsequent analytical characterization by LC-MS or RP-HPLC purification. The product was isolated using semipreparative RP-HPLC (Kromasil C18, 60 °C; 10 mL/min; linear gradient 5%–60%B in 50 min). Product containing fractions were pooled and lyophilized (Figure S57).

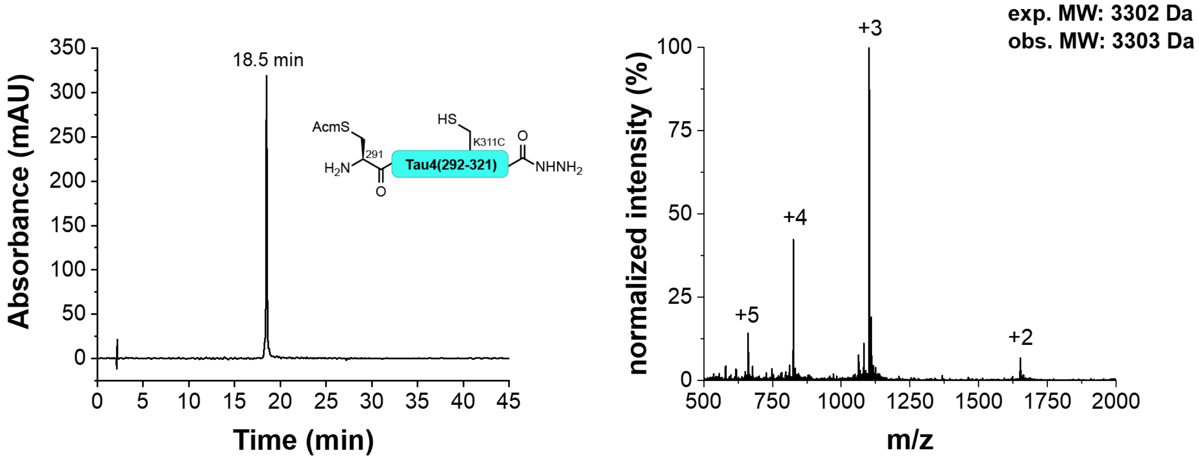

Figure S51. *Analytical RP-HPLC (C18) chromatogram and ESI-MS spectrum (exp. M.W.: 3302 Da; obs. M.W.: 3303 Da) of purified Tau4(291-321, C291Acm)-NHNH2.*

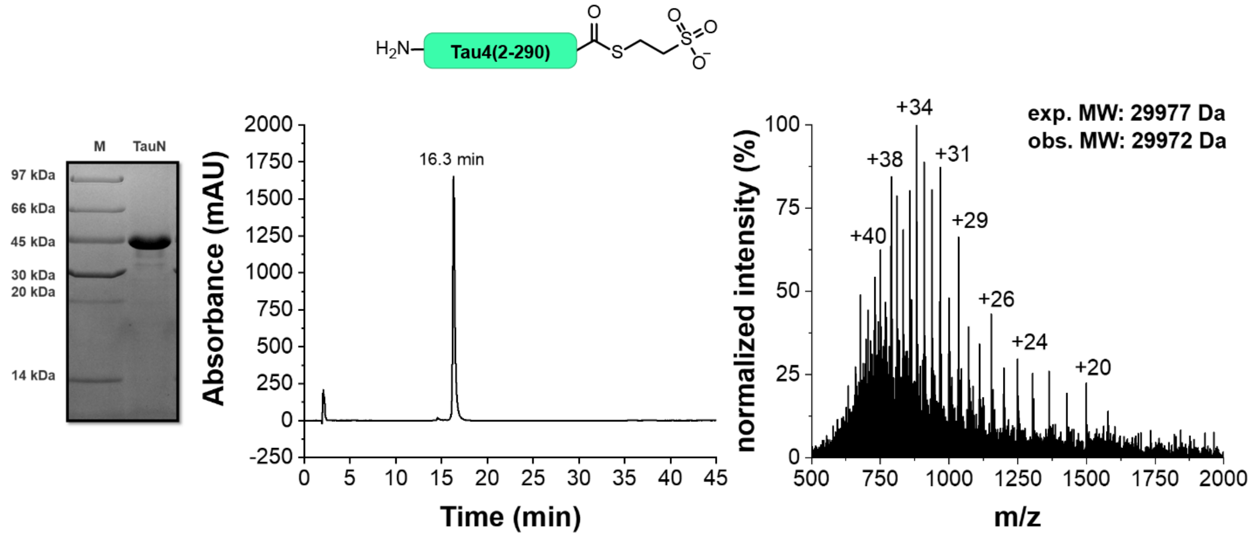

Figure S52. *Characterization of purified Tau4(2-290)-MesNa by SDS-PAGE, RP-HPLC (C4) and ESI-MS (exp. M.W.: 29977 Da; obs. M.W.: 29972 Da).*

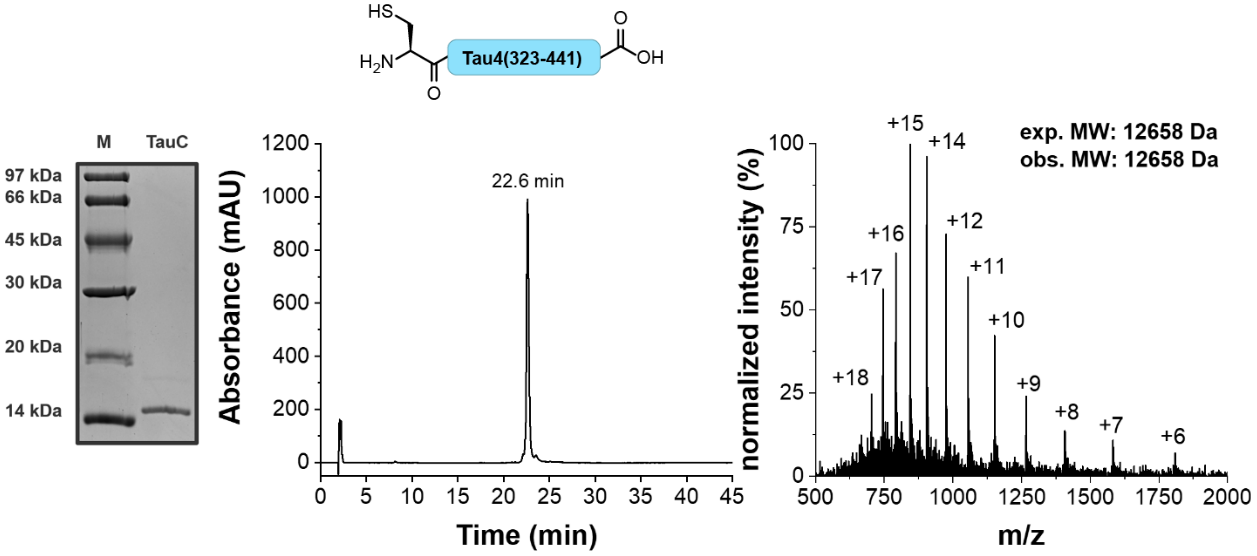

Figure S53. *Characterization of purified Tau4(322-441) by SDS-PAGE, RP-HPLC (C4) and ESI-MS (exp. M.W.: 12658 Da; obs. M.W.: 12658 Da).*

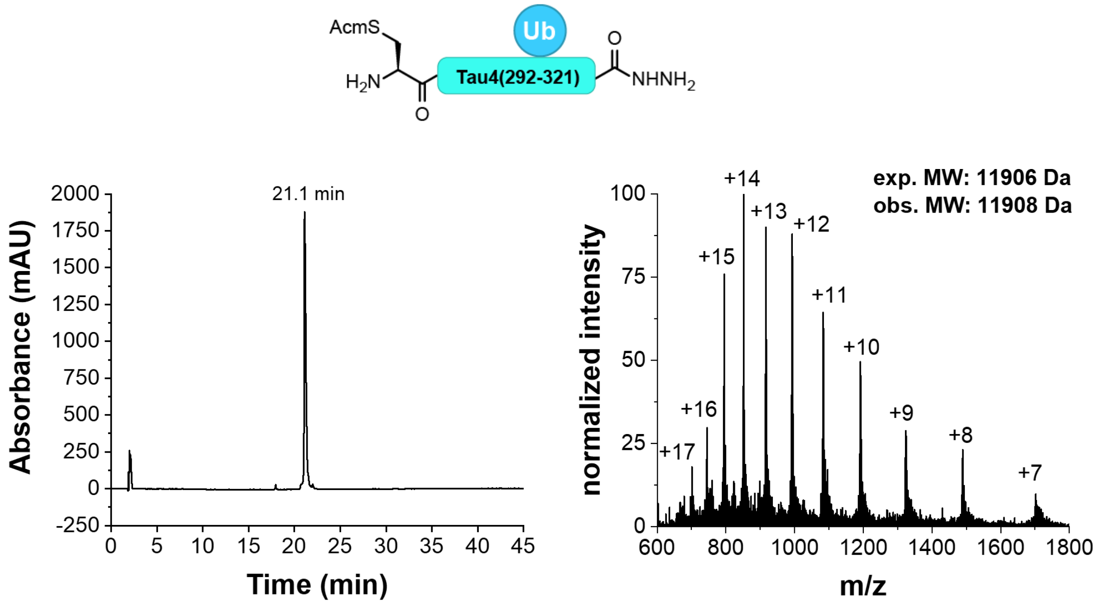

Figure S54. Characterization of Ub-Tau4(291-321, C291Acm)-NHNH₂. *Left: analytical RP-HPLC (C4) chromatogram (214 nm); right: ESI (pos.)-mass spectrum.*

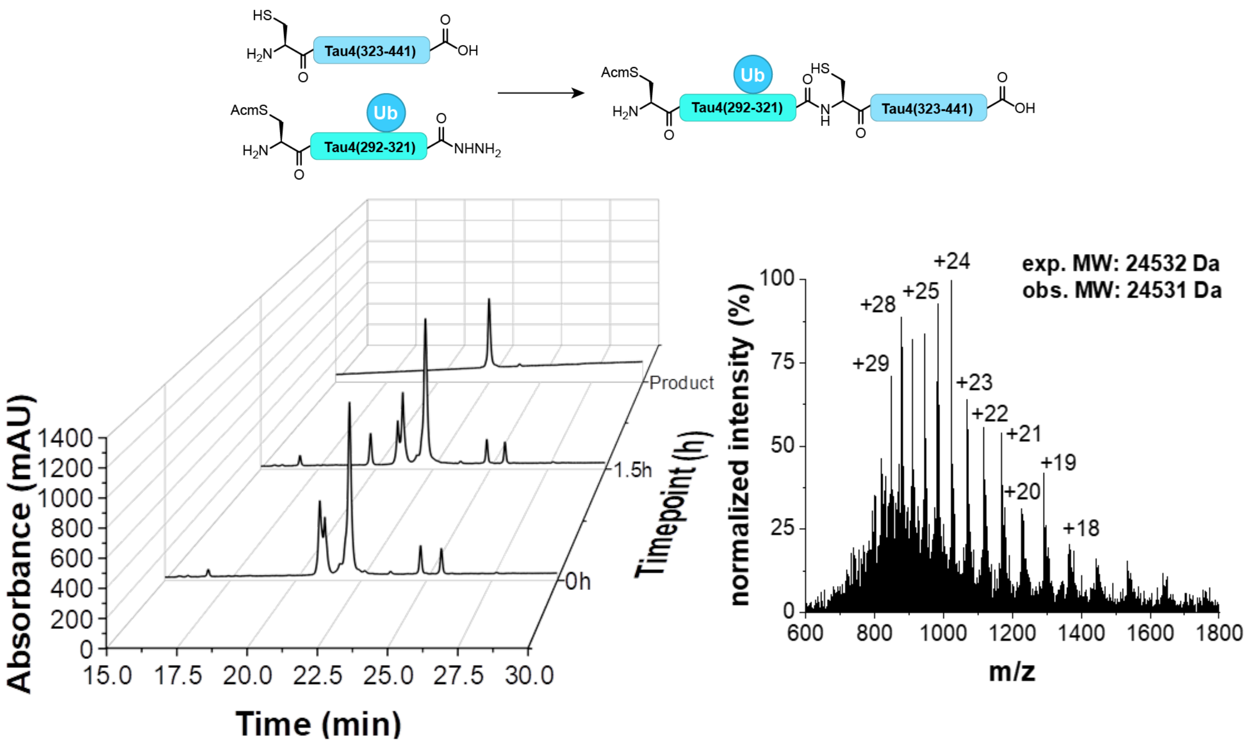

Figure S55. Ligation of Ub-Tau4(292-321, C291Acm)-NHNH₂ and Tau4(322-441). *Left: analytical RP-HPLC (C4) chromatogram (214 nm) of the reaction mixture at 0 h and 1.5 h and of the isolated product; right: ESI (pos.)-mass spectrum of the isolated Acm-protected Ub-Tau4(292-441,C291Acm).*

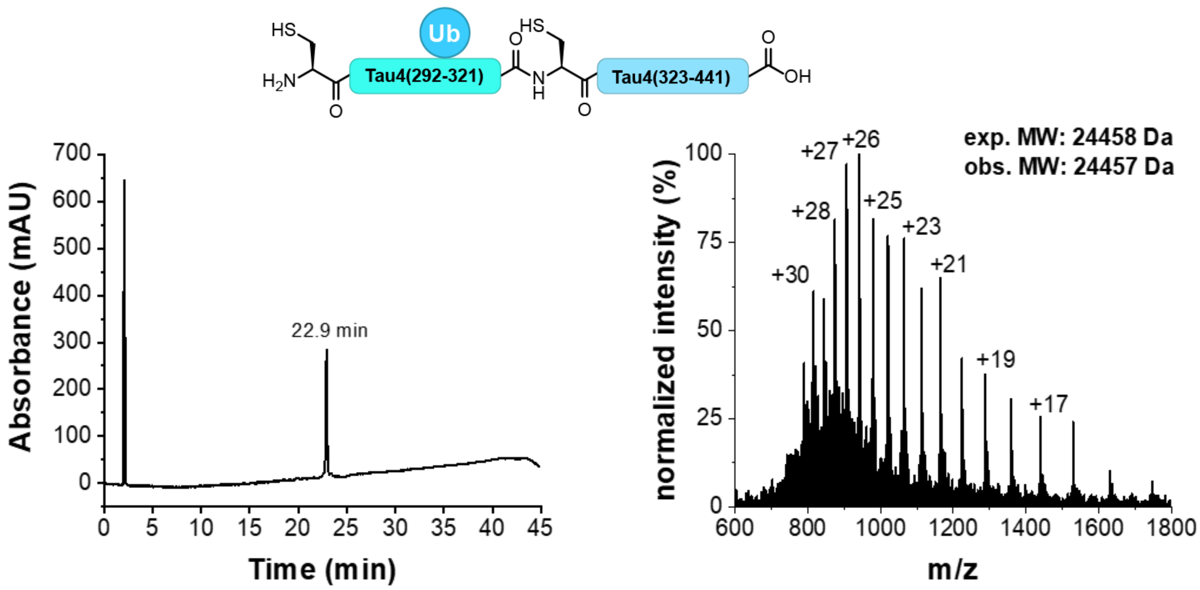

Figure S56. Characterization of Ub-Tau4(292-441) after Acm deprotection. *Left: analytical RP-HPLC (C4) chromatogram (214 nm); right: ESI (pos.)-mass spectrum.*

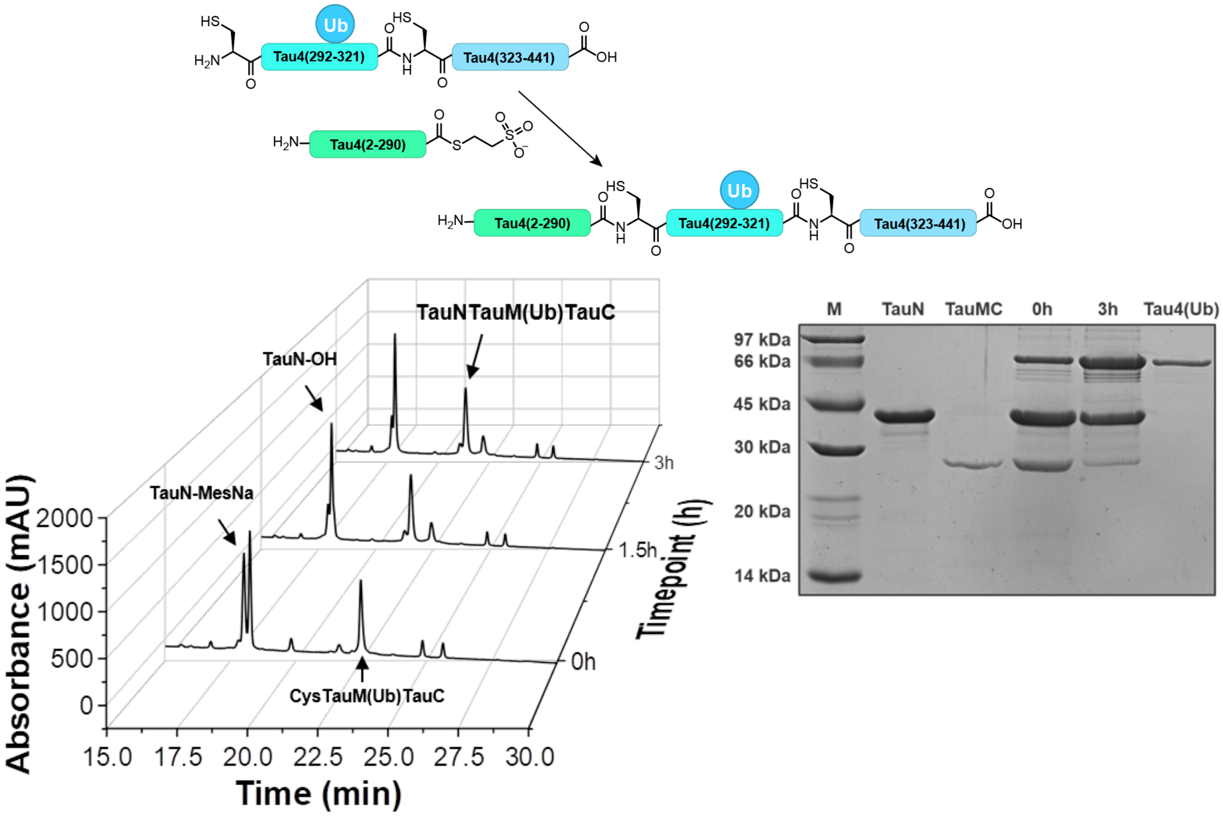

Figure S57. Figure 1: Ligation of Ub-Tau4(292-441) and Tau4(2-291)-MesNa. *Left: analytical RP-HPLC (C4) chromatogram (214 nm) of the reaction mixture at 0 h and 1.5 h and 3 h with indicated components; right: SDS-PAGE (Coomassie stain) of the starting materials, the reaction mixture after 0 h, and 3 h and the isolated product Ub-Tau4(2-441).*

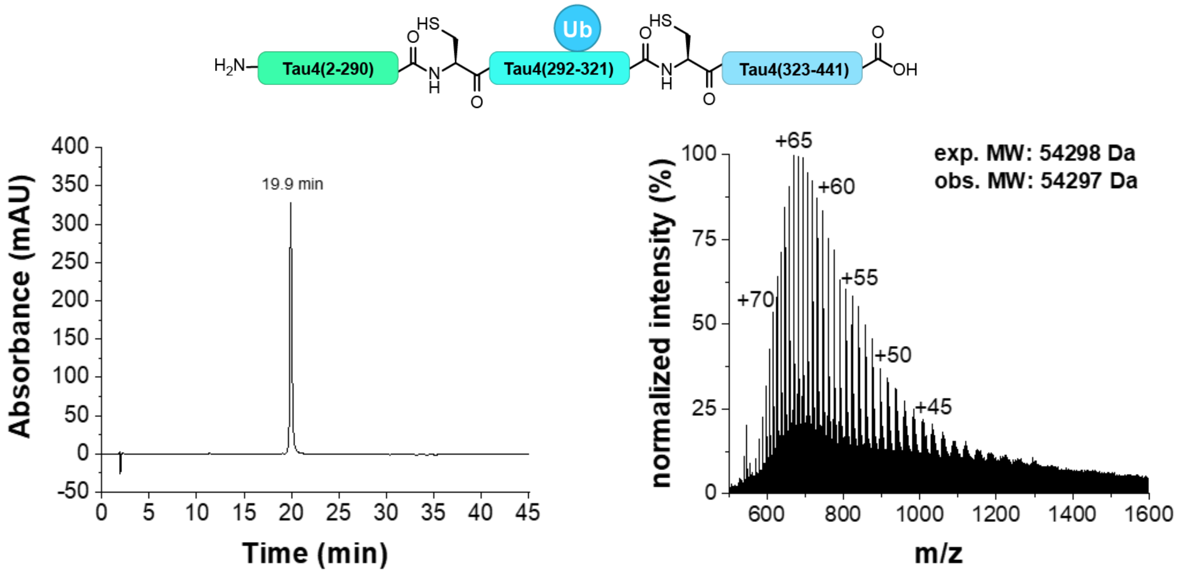

Figure S58. Characterization of Ub-Tau4(2-441). *Left: analytical RP-HPLC (C4) chromatogram (214 nm); right: ESI (pos.)-mass spectrum.*

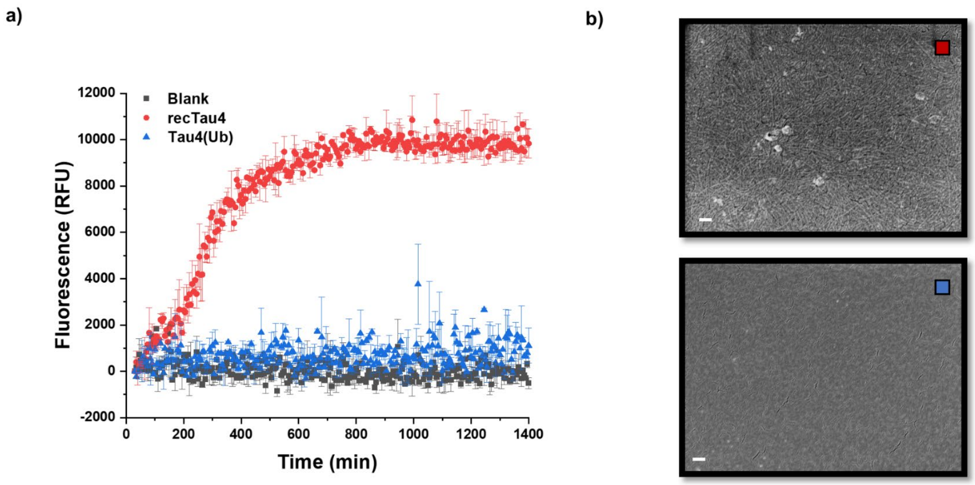

Figure S59. *a) ThT aggregation assay of tau ubiquitylated at position 311 (Tau4(Ub), blue) and unmodified recombinant tau (recTau4, red) (mean ± SEM, n = 2) and blank without Tau4 containing ThT dye (grey). b) SEM images of recombinant (red square) and ubiquitinated Tau4 (blue square) after ThT assays (scale bar: 200 nm).*

Amino acid and gene sequences

Ubiquitin wildtype:

Amino acid sequence:

MQIFVKTLTGKTITLEVEPSDTIENVKAKIQDKEGIPPDQQRLIFAGKQLEDGRTLSDYNIQKESTLHLVLRLRGG

Gene sequence for the expression of fusion construct Ub-Mxe-H7-CBD (codon optimized):

TCTAGAATAATTTTGTTTAACTTTAAGAAGGAGATATACATATGCAGATCTTCGTGAAGACTCTGACTGGTAAGAC
CATCACTCTCGAAGTGGAGCCGAGTGACACCATTGAGAATGTCAAGGCAAAGATCCAAGACAAGGAAGGCATCC
CTCCTGACCAGCAGAGGTTGATCTTTGCTGGGAAACAGCTGGAAGATGGACGCACCCTGTCTGACTACAACATC
CAGAAAGAGTCCACCCTGCACCTGGTACTCCGTCTCAGAGGTGGTTGCATCACGGGAGATGCACTAGTTGCCCT
ACCCGAGGGCGAGTCGGTACGCATCGCCGACATCGTGCCGGGTGCGCGGCCCAACAGTGACAACGCCATCGA
CCTGAAAGTCCTTGACCGGCATGGCAATCCCGTGCTCGCCGACCGGCTGTTCCACTCCGGCGAGCATCCGGTG
TACACGGTGCGTACGGTCGAAGGTCTGCGTGTGACGGGCACCGCGAACCACCCGTTGTTGTGTTTGGTCGACG
TCGCCGGGGTGCCGACCCTGCTGTGGAAGCTGATCGACGAAATCAAGCCGGGCGATTACGCGGTGATTCAACG
CAGCGCATTCAGCGTCGACTGTGCAGGTTTTGCCCGCGGGAAACCCGAATTTGCGCCCACAACCTACACAGTC
GGCGTCCCTGGACTGGTGCGTTTCTTGGAAGCACACCACCGAGACCCGGACGCCCAAGCTATCGCCGACGAGC
TGACCGACGGGCGGTTCTACTACGCGAAAGTCGCCAGTGTCACCGACGCCGGCGTGCAGCCGGTGTATAGCCT
TCGTGTCGACACGGCAGACCACGCGTTTATCACGAACGGGTTCGTCAGCCACGCTACTGGCCTCACCGGAATT
CACCACCACCACCACCACCACTCCGGTCTGAACTCAGGCCTCACGACAAATCCTGGTGTATCCGCTTGGCAGGT
CAACACAGCTTATACTGCGGGACAATTGGTCACATATAACGGCAAGACGTATAAATGTTTGCAGCCCCACACCTC
CTTGGCAGGATGGGAACCATCCAACGTTCCTGCCTTGTGGCAGCTTCAATGACTGCAGGAAGGGGATCCGGCT
GCTAACAAAGCCCGAAAGGAAGCTGAGTTGGCTGCTGCCACCGCTGAACAATAACTAGCATAACCCCTTGGGGC
CYYTAAACGGGTCTTGAGGGGTTTTTTGCTGAAAGGAGGAACTW
(green = beginning of Ub)

Translated amino acid sequence of the fusion construct Ub-Mxe-H7-CBD:

MQIFVKTLTGKTITLEVEPSDTIENVKAKIQDKEGIPPDQQRLIFAGKQLEDGRTLSDYNIQKESTLHLVLRLRGG
CITGDALVALPEGESVRIADIVPGARPNSDNAIDLKVLDRHGNPVLADRLFHSGEHPVYTVRTVEGLRVTGTANHPLLC
LVDVAGVPTLLWKLIDEIKPGDYAVIQRSAFSVDCAGFARGKPEFAPTTYTVGVPGLVRFLEAHHRDPDAQAIADELTD
GRFYYAKVASVTDAGVQPVYSLRVDTADHAFITNGFVSHATGLTGIHHHHHHHSGLNSGLTTNPGVSAWQVNTAYTA
GQLVTYNGKTYKCLQPHTSLAGWEPSNVPALWQLQ

Ubiquitin(K11C):

Amino acid sequence:

MQIFVKTLTGCTITLEVEPSDTIENVKAKIQDKEGIPPDQQRLIFAGKQLEDGRTLSDYNIQKESTLHLVLRLRGG

Gene sequence for the expression of fusion construct Ub(K11C)-Mxe-H7-CBD:

TCTAGAATAATTTTGTTTAACTTTAAGAAGGAGATATACATATGCAGATCTTCGTGAAGACTCTGACTGGTTGCAC
CATCACTCTCGAAGTGGAGCCGAGTGACACCATTGAGAATGTCAAGGCAAAGATCCAAGACAAGGAAGGCATCC
CTCCTGACCAGCAGAGGTTGATCTTTGCTGGGAAACAGCTGGAAGATGGACGCACCCTGTCTGACTACAACATC
CAGAAAGAGTCCACCCTGCACCTGGTACTCCGTCTCAGAGGTGGTTGCATCACGGGAGATGCACTAGTTGCCCT

ACCCGAGGGCGAGTCGGTACGCATCGCCGACATCGTGCCGGGTGCGCGGCCCAACAGTGACAACGCCATCGA
CCTGAAAGTCCTTGACCGGCATGGCAATCCCGTGCTCGCCGACCGGCTGTTCCACTCCGGCGAGCATCCGGTG
TACACGGTGCGTACGGTCGAAGGTCTGCGTGTGACGGGCACCGCGAACCACCCGTTGTTGTGTTTGGTCGACG
TCGCCGGGGTGCCGACCCTGCTGTGGAAGCTGATCGACGAAATCAAGCCGGGCGATTACGCGGTGATTCAACG
CAGCGCATTCAGCGTCGACTGTGCAGGTTTTGCCCGCGGGAAACCCGAATTTGCGCCCACAACCTACACAGTC
GGCGTCCCTGGACTGGTGCGTTTCTTGGAAGCACACCACCGAGACCCGGACGCCCAAGCTATCGCCGACGAGC
TGACCGACGGGCGGTTCTACTACGCGAAAGTCGCCAGTGTCACCGACGCCGGCGTGCAGCCGGTGTATAGCCT
TCGTGTCGACACGGCAGACCACGCGTTTATCACGAACGGGTTCGTCAGCCACGCTACTGGCCTCACCGGAATT
CACCACCACCACCACCACCACTCCGGTCTGAACTCAGGCCTCACGACAAATCCTGGTGTATCCGCTTGGCAGGT
CAACACAGCTTATACTGCGGGACAATTGGTCACATATAACGGCAAGACGTATAAATGTTTGCAGCCCCACACCTC
CTTGGCAGGATGGGAACCATCCAACGTTCCTGCCTTGTGGCAGCTTCAATGACTGCAGGAAGGGGATCCGGCT
GCTAACAAAGCCCGAAAGGAAGCTGAGTTGGCTGCTGCCACCGCTGAACAATAACTAGCATAACCCCTTGGGGC
CYYTAAACGGGTCTTGAGGGGTTTTTTGCTGAAAGGAGGAACTW
(green = beginning of Ub; yellow = Cys mutation)

Translated amino acid sequence of the fusion construct Ub(K11C)-Mxe-H7-CBD:

MQIFVKTLTGCTITLEVEPSDTIENVKAKIQDKEGIPPDQQRLIFAGKQLEDGRTLSDYNIQKESTLHLVLRLRGG
CITGDALVALPEGESVRIADIVPGARPNSDNAIDLKVLDRHGNPVLADRLFHSGEHPVYTVRTVEGLRVTGTANHPLLC
LVDVAGVPTLLWKLIDEIKPGDYAVIQRSAFSVDCAGFARGKPEFAPTTYTVGVPGLVRFLEAHHRDPDAQAIADELTD
GRFYYAKVASVTDAGVQPVYSLRVDTADHAFITNGFVSHATGLTGIHHHHHHHSGLNSGLTTNPGVSAWQVNTAYTA
GQLVTYNGKTYKCLQPHTSLAGWEPSNVPALWQLQ

Ubiquitin(K27C):

Amino acid sequence:

MQIFVKTLTGKTITLEVEPSDTIENVCAKIQDKEGIPPDQQRLIFAGKQLEDGRTLSDYNIQKESTLHLVLRLRGG

Gene sequence for the expression of fusion construct Ub(K27C)-Mxe-H7-CBD:

TCTAGAATAATTTTGTTTAACTTTAAGAAGGAGATATACATATGCAGATCTTCGTGAAGACTCTGACTGGTAAGAC
CATCACTCTCGAAGTGGAGCCGAGTGACACCATTGAGAATGTCTGCGCAAAGATCCAAGACAAGGAAGGCATCC
CTCCTGACCAGCAGAGGTTGATCTTTGCTGGGAAACAGCTGGAAGATGGACGCACCCTGTCTGACTACAACATC
CAGAAAGAGTCCACCCTGCACCTGGTACTCCGTCTCAGAGGTGGTTGCATCACGGGAGATGCACTAGTTGCCCT
ACCCGAGGGCGAGTCGGTACGCATCGCCGACATCGTGCCGGGTGCGCGGCCCAACAGTGACAACGCCATCGA
CCTGAAAGTCCTTGACCGGCATGGCAATCCCGTGCTCGCCGACCGGCTGTTCCACTCCGGCGAGCATCCGGTG
TACACGGTGCGTACGGTCGAAGGTCTGCGTGTGACGGGCACCGCGAACCACCCGTTGTTGTGTTTGGTCGACG
TCGCCGGGGTGCCGACCCTGCTGTGGAAGCTGATCGACGAAATCAAGCCGGGCGATTACGCGGTGATTCAACG
CAGCGCATTCAGCGTCGACTGTGCAGGTTTTGCCCGCGGGAAACCCGAATTTGCGCCCACAACCTACACAGTC
GGCGTCCCTGGACTGGTGCGTTTCTTGGAAGCACACCACCGAGACCCGGACGCCCAAGCTATCGCCGACGAGC
TGACCGACGGGCGGTTCTACTACGCGAAAGTCGCCAGTGTCACCGACGCCGGCGTGCAGCCGGTGTATAGCCT
TCGTGTCGACACGGCAGACCACGCGTTTATCACGAACGGGTTCGTCAGCCACGCTACTGGCCTCACCGGAATT
CACCACCACCACCACCACCACTCCGGTCTGAACTCAGGCCTCACGACAAATCCTGGTGTATCCGCTTGGCAGGT
CAACACAGCTTATACTGCGGGACAATTGGTCACATATAACGGCAAGACGTATAAATGTTTGCAGCCCCACACCTC
CTTGGCAGGATGGGAACCATCCAACGTTCCTGCCTTGTGGCAGCTTCAATGACTGCAGGAAGGGGATCCGGCT
GCTAACAAAGCCCGAAAGGAAGCTGAGTTGGCTGCTGCCACCGCTGAACAATAACTAGCATAACCCCTTGGGGC
CYYTAAACGGGTCTTGAGGGGTTTTTTGCTGAAAGGAGGAACTW
(green = beginning of Ub; yellow = Cys mutation)

Translated amino acid sequence of the fusion construct Ub(K27C)-Mxe-H7-CBD:

MQIFVKTLTGKTITLEVEPSDTIENVCAKIQDKEGIPPDQQRLIFAGKQLEDGRTLSDYNIQKESTLHLVLRLRGG
CITGDALVALPEGESVRIADIVPGARPNSDNAIDLKVLDRHGNPVLADRLFHSGEHPVYTVRTVEGLRVTGTANHPLLC
LVDVAGVPTLLWKLIDEIKPGDYAVIQRSAFSVDCAGFARGKPEFAPTTYTVGVPGLVRFLEAHHRDPDAQAIADELTD
GRFYYAKVASVTDAGVQPVYSLRVDTADHAFITNGFVSHATGLTGIHHHHHHHSGLNSGLTTNPGVSAWQVNTAYTA
GQLVTYNGKTYKCLQPHTSLAGWEPSNVPALWQLQ

Ubiquitin(K29C):

Amino acid sequence:

MQIFVKTLTGKTITLEVEPSDTIENVKACIQDKEGIPPDQQRLIFAGKQLEDGRTLSDYNIQKESTLHLVLRLRGG

Gene sequence for the expression of fusion construct Ub(K29C)-Mxe-H7-CBD:

TCTAGAATAATTTTGTTTAACTTTAAGAAGGAGATATACATATGCAGATCTTCGTGAAGACTCTGACTGGTAAGAC
CATCACTCTCGAAGTGGAGCCGAGTGACACCATTGAGAATGTCAAGGCATGCATCCAAGACAAGGAAGGCATCC
CTCCTGACCAGCAGAGGTTGATCTTTGCTGGGAAACAGCTGGAAGATGGACGCACCCTGTCTGACTACAACATC
CAGAAAGAGTCCACCCTGCACCTGGTACTCCGTCTCAGAGGTGGTTGCATCACGGGAGATGCACTAGTTGCCCT
ACCCGAGGGCGAGTCGGTACGCATCGCCGACATCGTGCCGGGTGCGCGGCCCAACAGTGACAACGCCATCGA
CCTGAAAGTCCTTGACCGGCATGGCAATCCCGTGCTCGCCGACCGGCTGTTCCACTCCGGCGAGCATCCGGTG
TACACGGTGCGTACGGTCGAAGGTCTGCGTGTGACGGGCACCGCGAACCACCCGTTGTTGTGTTTGGTCGACG
TCGCCGGGGTGCCGACCCTGCTGTGGAAGCTGATCGACGAAATCAAGCCGGGCGATTACGCGGTGATTCAACG
CAGCGCATTCAGCGTCGACTGTGCAGGTTTTGCCCGCGGGAAACCCGAATTTGCGCCCACAACCTACACAGTC
GGCGTCCCTGGACTGGTGCGTTTCTTGGAAGCACACCACCGAGACCCGGACGCCCAAGCTATCGCCGACGAGC
TGACCGACGGGCGGTTCTACTACGCGAAAGTCGCCAGTGTCACCGACGCCGGCGTGCAGCCGGTGTATAGCCT
TCGTGTCGACACGGCAGACCACGCGTTTATCACGAACGGGTTCGTCAGCCACGCTACTGGCCTCACCGGAATT
CACCACCACCACCACCACCACTCCGGTCTGAACTCAGGCCTCACGACAAATCCTGGTGTATCCGCTTGGCAGGT
CAACACAGCTTATACTGCGGGACAATTGGTCACATATAACGGCAAGACGTATAAATGTTTGCAGCCCCACACCTC
CTTGGCAGGATGGGAACCATCCAACGTTCCTGCCTTGTGGCAGCTTCAATGACTGCAGGAAGGGGATCCGGCT
GCTAACAAAGCCCGAAAGGAAGCTGAGTTGGCTGCTGCCACCGCTGAACAATAACTAGCATAACCCCTTGGGGC
CYYTAAACGGGTCTTGAGGGGTTTTTTGCTGAAAGGAGGAACTW
(green = beginning of Ub; yellow = Cys mutation)

Translated amino acid sequence of the fusion construct Ub(K29C)-Mxe-H7-CBD:

MQIFVKTLTGKTITLEVEPSDTIENVKACIQDKEGIPPDQQRLIFAGKQLEDGRTLSDYNIQKESTLHLVLRLRGG
CITGDALVALPEGESVRIADIVPGARPNSDNAIDLKVLDRHGNPVLADRLFHSGEHPVYTVRTVEGLRVTGTANHPLLC
LVDVAGVPTLLWKLIDEIKPGDYAVIQRSAFSVDCAGFARGKPEFAPTTYTVGVPGLVRFLEAHHRDPDAQAIADELTD
GRFYYAKVASVTDAGVQPVYSLRVDTADHAFITNGFVSHATGLTGIHHHHHHHSGLNSGLTTNPGVSAWQVNTAYTA
GQLVTYNGKTYKCLQPHTSLAGWEPSNVPALWQLQ

Ubiquitin(K48C):

Amino acid sequence:

MQIFVKTLTGKTITLEVEPSDTIENVKAKIQDKEGIPPDQQRLIFAGCQLEDGRTLSDYNIQKESTLHLVLRLRGG

Gene sequence for the expression of fusion construct Ub(K48C)-Mxe-H7-CBD:

TCTAGAATAATTTTGTTTAACTTTAAGAAGGAGATATACATATGCAGATCTTCGTGAAGACTCTGACTGGTAAGAC
CATCACTCTCGAAGTGGAGCCGAGTGACACCATTGAGAATGTCAAGGCAAAGATCCAAGACAAGGAAGGCATCC
CTCCTGACCAGCAGAGGTTGATCTTTGCTGGGTGCCAGCTGGAAGATGGACGCACCCTGTCTGACTACAACATC
CAGAAAGAGTCCACCCTGCACCTGGTACTCCGTCTCAGAGGTGGTTGCATCACGGGAGATGCACTAGTTGCCCT
ACCCGAGGGCGAGTCGGTACGCATCGCCGACATCGTGCCGGGTGCGCGGCCCAACAGTGACAACGCCATCGA
CCTGAAAGTCCTTGACCGGCATGGCAATCCCGTGCTCGCCGACCGGCTGTTCCACTCCGGCGAGCATCCGGTG
TACACGGTGCGTACGGTCGAAGGTCTGCGTGTGACGGGCACCGCGAACCACCCGTTGTTGTGTTTGGTCGACG
TCGCCGGGGTGCCGACCCTGCTGTGGAAGCTGATCGACGAAATCAAGCCGGGCGATTACGCGGTGATTCAACG
CAGCGCATTCAGCGTCGACTGTGCAGGTTTTGCCCGCGGGAAACCCGAATTTGCGCCCACAACCTACACAGTC

GGCGTCCCTGGACTGGTGCGTTTCTTGGAAGCACACCACCGAGACCCGGACGCCCAAGCTATCGCCGACGAGC
TGACCGACGGGCGGTTCTACTACGCGAAAGTCGCCAGTGTCACCGACGCCGGCGTGCAGCCGGTGTATAGCCT
TCGTGTCGACACGGCAGACCACGCGTTTATCACGAACGGGTTCGTCAGCCACGCTACTGGCCTCACCGGAATT
CACCACCACCACCACCACCACTCCGGTCTGAACTCAGGCCTCACGACAAATCCTGGTGTATCCGCTTGGCAGGT
CAACACAGCTTATACTGCGGGACAATTGGTCACATATAACGGCAAGACGTATAAATGTTTGCAGCCCCACACCTC
CTTGGCAGGATGGGAACCATCCAACGTTCCTGCCTTGTGGCAGCTTCAATGACTGCAGGAAGGGGATCCGGCT
GCTAACAAAGCCCGAAAGGAAGCTGAGTTGGCTGCTGCCACCGCTGAACAATAACTAGCATAACCCCTTGGGGC
CYYTAAACGGGTCTTGAGGGGTTTTTTGCTGAAAGGAGGAACTW
(green = beginning of Ub; yellow = Cys mutation)

Translated amino acid sequence of the fusion construct Ub(K48C)-Mxe-H7-CBD:

MQIFVKTLTGKTITLEVEPSDTIENVKAKIQDKEGIPPDQQRLIFAGCQLEDGRTLSDYNIQKESTLHLVLRLRGG
CITGDALVALPEGESVRIADIVPGARPNSDNAIDLKVLDRHGNPVLADRLFHSGEHPVYTVRTVEGLRVTGTANHPLLC
LVDVAGVPTLLWKLIDEIKPGDYAVIQRSAFSVDCAGFARGKPEFAPTTYTVGVPGLVRFLEAHHRDPDAQAIADELTD
GRFYYAKVASVTDAGVQPVYSLRVDTADHAFITNGFVSHATGLTGIHHHHHHHSGLNSGLTTNPGVSAWQVNTAYTA
GQLVTYNGKTYKCLQPHTSLAGWEPSNVPALWQLQ

Ubiquitin(K63C):

Amino acid sequence:

MQIFVKTLTGKTITLEVEPSDTIENVKAKIQDKEGIPPDQQRLIFAGCQLEDGRTLSDYNIQCESTLHLVLRLRGG

Gene sequence for the expression of fusion construct Ub(K63C)-Mxe-H7-CBD:

TCTAGAATAATTTTGTTTAACTTTAAGAAGGAGATATACATATGCAGATCTTCGTGAAGACTCTGACTGGTAAGAC
CATCACTCTCGAAGTGGAGCCGAGTGACACCATTGAGAATGTCAAGGCAAAGATCCAAGACAAGGAAGGCATCC
CTCCTGACCAGCAGAGGTTGATCTTTGCTGGGAAACAGCTGGAAGATGGACGCACCCTGTCTGACTACAACATC
CAGTGCGAGTCCACCCTGCACCTGGTACTCCGTCTCAGAGGTGGTTGCATCACGGGAGATGCACTAGTTGCCCT
ACCCGAGGGCGAGTCGGTACGCATCGCCGACATCGTGCCGGGTGCGCGGCCCAACAGTGACAACGCCATCGA
CCTGAAAGTCCTTGACCGGCATGGCAATCCCGTGCTCGCCGACCGGCTGTTCCACTCCGGCGAGCATCCGGTG
TACACGGTGCGTACGGTCGAAGGTCTGCGTGTGACGGGCACCGCGAACCACCCGTTGTTGTGTTTGGTCGACG
TCGCCGGGGTGCCGACCCTGCTGTGGAAGCTGATCGACGAAATCAAGCCGGGCGATTACGCGGTGATTCAACG
CAGCGCATTCAGCGTCGACTGTGCAGGTTTTGCCCGCGGGAAACCCGAATTTGCGCCCACAACCTACACAGTC
GGCGTCCCTGGACTGGTGCGTTTCTTGGAAGCACACCACCGAGACCCGGACGCCCAAGCTATCGCCGACGAGC
TGACCGACGGGCGGTTCTACTACGCGAAAGTCGCCAGTGTCACCGACGCCGGCGTGCAGCCGGTGTATAGCCT
TCGTGTCGACACGGCAGACCACGCGTTTATCACGAACGGGTTCGTCAGCCACGCTACTGGCCTCACCGGAATT
CACCACCACCACCACCACCACTCCGGTCTGAACTCAGGCCTCACGACAAATCCTGGTGTATCCGCTTGGCAGGT
CAACACAGCTTATACTGCGGGACAATTGGTCACATATAACGGCAAGACGTATAAATGTTTGCAGCCCCACACCTC
CTTGGCAGGATGGGAACCATCCAACGTTCCTGCCTTGTGGCAGCTTCAATGACTGCAGGAAGGGGATCCGGCT
GCTAACAAAGCCCGAAAGGAAGCTGAGTTGGCTGCTGCCACCGCTGAACAATAACTAGCATAACCCCTTGGGGC
CYYTAAACGGGTCTTGAGGGGTTTTTTGCTGAAAGGAGGAACTW
(green = beginning of Ub; yellow = Cys mutation)

Translated amino acid sequence of the fusion construct Ub(K63C)-Mxe-H7-CBD:

MQIFVKTLTGKTITLEVEPSDTIENVKAKIQDKEGIPPDQQRLIFAGCQLEDGRTLSDYNIQCESTLHLVLRLRGG
CITGDALVALPEGESVRIADIVPGARPNSDNAIDLKVLDRHGNPVLADRLFHSGEHPVYTVRTVEGLRVTGTANHPLLC
LVDVAGVPTLLWKLIDEIKPGDYAVIQRSAFSVDCAGFARGKPEFAPTTYTVGVPGLVRFLEAHHRDPDAQAIADELTD
GRFYYAKVASVTDAGVQPVYSLRVDTADHAFITNGFVSHATGLTGIHHHHHHHSGLNSGLTTNPGVSAWQVNTAYTA
GQLVTYNGKTYKCLQPHTSLAGWEPSNVPALWQLQ

Tau(2-290):

Amino acid sequence:

CGSLGNIHHKPGGGQVEVKSEKLDFKDRVQSKIGSLDNITHVPGGGNKKIETHKLTFRENAKAKTDHGAEIVYKSPVV
SGDTSPRHLSNVSSTGSIDMVDSPQLATLADEVSASLAKQGL

Translated amino acid sequence of the fusion construct Tau(1-290)-Mxe-H7-CBD:

MAEPRQEFEVMEDHAGTYGLGDRKDQGGYTMHQDQEGDTDAGLKESPLQTPTEDGSEEPGSETSDAKSTPTAEDVT
APLVDEGAPGKQAAAQPHTEIPEGTTAEEAGIGDTPSLEDEAAGHVTQARMVSKSKDGTGSDDKKAKGADGKTKIATP
RGAAPPGQKGQANATRIPAKTPPAPKTPPSSGEPPKSGDRSGYSSPGSPGTPGSRSRTPSLPTPPTREPKKVAVVRTPPK
SPSSAKSRLQTAPVPMPDLKNVKSKIGSTENLKHQPGGGKVQIINKKLDLSNVQSKCICGDALVALPEGESVRIADIVPGA
RPNSDNAIDLKVLDRHGNPVLADRLFHSGEHPVYTVRTVEGLRVTGTANHPLLCLVDVAGVPTLLWKLIDEIKPGDYAVI
QRSAFSVDCAGFARGKPEFAPTTYTVGVPGLVRFLEAHHRDPDAQAIADELTDGRFYYAKVASVTDAGVQPVYSLRVD
TADHAFITNGFVSHATGLTGIHHHHHHHSGLNSGLTTNPGVSAWQVNTAYTAGQLVTYNGKTYKCLQPHTSLAGWEP
SNVPALWQLQ

(green = Tau4(1–290); black = Mxe GyrA intein; orange = His-tag; blue = chitin binding domain)

Tau(322-441):

Amino acid sequence:

CGSLGNIHHKPGGGQVEVKSEKLDFKDRVQSKIGSLDNITHVPGGGNKKIETHKLTFRENAKAKTDHGAEIVYKSPVV
SGDTSPRHLSNVSSTGSIDMVDSPQLATLADEVSASLAKQGL

Translated amino acid sequence of the fusion construct H6-TEV-Tau(322-441):

HHHHHHSSGLVPRGSHSSYENLYFQCGSLGNIHHKPGGGQVEVKSEKLDFKDRVQSKIGSLDNITHVPGGGNKKIET
HKLTFRENAKAKTDHGAEIVYKSPVVSGDTSPRHLSNVSSTGSIDMVDSPQLATLADEVSASLAKQGL

(green = His-tag, purple = TEV-cleavage sequence; blue = Tau4(322–441)

References

[35] S. Mukherjee, M. Matveenko, C. F. W. Becker, in *Expressed Protein Ligation: Methods and Protocols* (Ed.: M. Vila-Perelló), Springer, **2020**, pp. 343-358.

[44] D. Ellmer, M. Brehs, M. Haj-Yahya, H. A. Lashuel, C. F. W. Becker, *Angew Chem Int Ed Engl* **2019**, *58*, 1616-1620.

[52] P. Wang, S. Dong, J. H. Shieh, E. Peguero, R. Hendrickson, M. A. S. Moore, S. J. Danishefsky, *Science* **2013**, *342*, 1357-1360.
